# Single cell polarity in liquid phase facilitates tumour metastasis

**DOI:** 10.1038/s41467-018-03139-6

**Published:** 2018-02-28

**Authors:** Anna Lorentzen, Paul F. Becker, Jan Kosla, Massimo Saini, Kathrin Weidele, Paolo Ronchi, Corinna Klein, Monika J. Wolf, Felix Geist, Bastian Seubert, Marc Ringelhan, Daniela Mihic-Probst, Knud Esser, Marko Roblek, Felix Kuehne, Gaia Bianco, Tracy O’Connor, Quentin Müller, Kathleen Schuck, Sebastian Lange, Daniel Hartmann, Saskia Spaich, Olaf Groß, Jochen Utikal, Sebastian Haferkamp, Martin R. Sprick, Amruta Damle-Vartak, Alexander Hapfelmeier, Norbert Hüser, Ulrike Protzer, Andreas Trumpp, Dieter Saur, Nachiket Vartak, Christoph A. Klein, Bernhard Polzer, Lubor Borsig, Mathias Heikenwalder

**Affiliations:** 1 Institute of Virology, Technische Universität München, Helmholtz Center Munich (HMGU), 81675 Munich, Germany; 2 Department of Molecular Biology and Genetics, Aarhus University, 8000 Aarhus, Denmark; 3 Institute of Molecular Genetics of the Academy of Sciences of the Czech Republic, 14220 Prague, Czech Republic; 4 Divison of Chronic Inflammation and Cancer, German Cancer Research Center (DKFZ), 69120 Heidelberg, Germany; 5 Division of Stem Cells and Cancer, Deutsches Krebsforschungszentrum (DKFZ), 69120 Heidelberg, Germany; 6 Heidelberg Institute for Stem Cell Technology and Experimental Medicine (HI-STEM) gGmbH, 69120 Heidelberg, Germany; 7 Project Group Personalized Tumour Therapy, Fraunhofer-Institut für Toxikologie und Experimentelle Medizin, 93053 Regensburg, Germany; 8 Electron Microscopy Core Facility, European Molecular Biology Laboratory (EMBL), 69117 Heidelberg, Germany; 9 Department of Surgical Pathology, University Hospital Zurich, 8091 Zurich, Switzerland; 10 Department of Internal Medicine II, Klinikum rechts der Isar, Technische Universität München, 81675 Munich, Germany; 11 Institute of Physiology, Zurich Center for Integrative Human Physiology, University of Zurich, 8057 Zurich, Switzerland; 12 Department of Surgery, Klinikum rechts der Isar, Technische Universität München, 81675 Munich, Germany; 13 Frauenklinik, University Medical Centre Mannheim, 68167 Mannheim, Germany; 14 Institut für Klinische Chemie und Pathobiochemie, Klinikum rechts der Isar, Technische Universität München, 81675 Munich, Germany; 15 Skin Cancer Unit, Deutsches Krebsforschungszentrum (DKFZ), 69120 Heidelberg, Germany; 16 Department of Dermatology, Venereology and Allergology, University Medical Center Mannheim, Ruprecht-Karl University of Heidelberg, 68167 Mannheim, Germany; 17 Department of Dermatology, University Hospital Regensburg, 93042 Regensburg, Germany; 18 German Cancer Consortium (DKTK) and German Cancer Research Center (DKFZ), 69120 Heidelberg, Germany; 19 Department of Systems Toxicology, Leibnitz Research Cenrtre for Working Environment and Human Factors (IfADo), 44139 Dortmund, Germany; 20 Institut für Medizinische Statistik und Epidemiologie, Klinikum rechts der Isar, Technische Universität München, 81675 Munich, Germany; 21 Chair of Experimental Medicine and Therapy Research, University of Regensburg, 93053 Regensburg, Germany

## Abstract

Dynamic polarisation of tumour cells is essential for metastasis. While the role of polarisation during dedifferentiation and migration is well established, polarisation of metastasising tumour cells during phases of detachment has not been investigated. Here we identify and characterise a type of polarisation maintained by single cells in liquid phase termed single-cell (sc) polarity and investigate its role during metastasis. We demonstrate that sc polarity is an inherent feature of cells from different tumour entities that is observed in circulating tumour cells in patients. Functionally, we propose that the sc pole is directly involved in early attachment, thereby affecting adhesion, transmigration and metastasis. In vivo, the metastatic capacity of cell lines correlates with the extent of sc polarisation. By manipulating sc polarity regulators and by generic depolarisation, we show that sc polarity prior to migration affects transmigration and metastasis in vitro and in vivo.

## Abstract (web summary)

Polarisation of metastasising cancer cells in circulation has not been investigated before. Here the authors identify single cell polarity as a distinct polarisation state of single cells in liquid phase, and show that perturbing single cell polarity affects attachment, adhesion, transmigration and metastasis in vitro and in vivo.

## Introduction

Metastases are the major cause of cancer-related deaths^1,2^. Despite novel promising targeted cancer therapies, patients diagnosed with systemic metastatic disease are no longer eligible for curative treatment options in many cancer subtypes^3–5^ necessitating research on additional, broadly applicable strategies for metastasis intervention. Metastasis is a multistep process comprising dedifferentiation, dissociation and local invasion of primary tumour cells, intravasation into blood or lymph vessels, survival and transport in circulation, arrest in microvessels of distant organs and extravasation and metastatic outgrowth^6^.

Throughout the metastatic process, solid tumour cells establish distinct types of polarity, such as apical–basal polarity in the tissue context of established primary or metastatic tumours or front–back polarity during migratory phases^7,8^. The metastatic cascade thus involves dynamic depolarisation and repolarisation of metastasising cells, reflecting their high plasticity. However, the polarisation of cells during liquid or detached phases and the relevance of such polarisation for metastasis have remained unclear.

Here we identify a distinct type of polarity termed single-cell (sc) polarity that tumour cells maintain in liquid phase. Sc polarity is defined by the intrinsic presence of an ezrin- and actin-rich pole in absence of an extracellular stimulus in non-adhering, non-migrating cells. We characterise sc polarity in tumour cell lines and human tumour specimens from biopsies collected in liquid phase and investigate the role of sc polarity in human tumour cells, mouse models of metastasis and ex vivo. We find that sc polarity affects attachment, adhesion, transmigration and metastasis.

## Results

### Tumour cells maintain their polarity in liquid phase

**Fig. 1:**
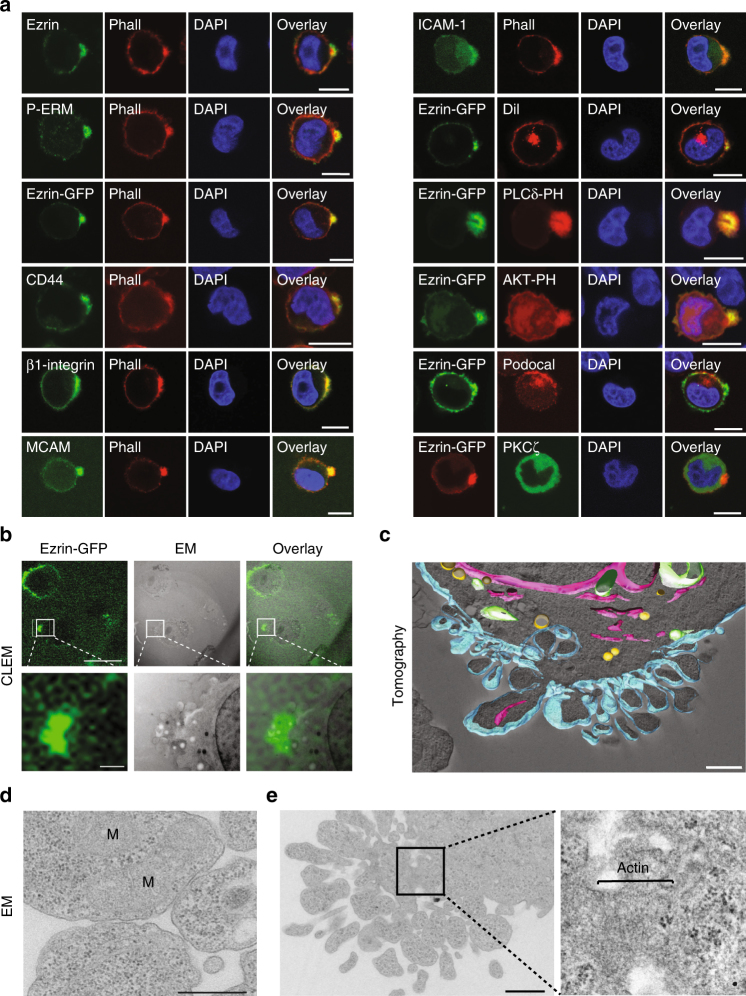
Characterization of sc polarity. **a** SkMel2 cells in suspension transfected with the indicated plasmids (PLCδ-PH, Akt-PH: RFP-labelled PIP_2_-binding PH domains of PLCδ and Akt/PKB), stained with phalloidin or DiI and DAPI and the indicated antibodies (P-ERM: phosphorylated ezrin/radixin/moesin, podocal: podocalyxin). Further PH domains are shown in Supplementary Fig. 1a. Scale bars: 10 µm. **b** Correlative light and electron microscopy (CLEM) of SkMel2 cells expressing ezrin-GFP. The same areas from 300 nm sections were imaged by fluorescence microscopy (GFP) and EM. Scale bars: 20 µm (top) and 2 µm (bottom). **c** 3D reconstruction of a 300 nm section through the pole of an SkMel2 cell in suspension. Plasma membrane is shown in cyan, ER and nuclear envelope in pink, mitochondria in green and lipid droplets in yellow. The raw tomography stack as well as the 3D rendering are shown in Supplementary Movie 1. Scale bar: 1 µm. **d** Transmission EM of the pole of an SkMel2 cell (70 nm section). M indicates mitochondria. Scale bar: 1 µm. **e** Transmission EM of the pole of an SkMel2 cell and in detail the neck of this pole, which displays fibrillar structures compatible in size and organisation with the actin cytoskeleton. Scale bar: 1 µm

To investigate sc polarity in tumour cells in liquid phase, polarity markers of different polar structures of single cells^9–13^ were imaged in human SkMel2 melanoma cells in suspension (Fig. 1a). Ezrin-green fluorescent protein (GFP) as well as endogenous ezrin, moesin, Radixin-GFP and phosphorylated ezrin/radixin/moesin proteins accumulated at one pole of single cells in suspension (Fig. 1a and Supplementary Fig. 1a). Additionally, polar accumulation of F-actin and the plasma membrane (PM) receptors CD44, β1-Integrin, melanoma cell adhesion molecule (MCAM) and intercellular adhesion molecule-1 (ICAM-1) was observed (Fig. 1a). The PM itself was accumulated at the pole and enriched with phosphatidylinositol 4,5-bisphosphate (PIP_2_, Fig. 1a and Supplementary Fig. 1a) while the polarity regulator Protein Kinase C ζ did not co-localize with the ezrin pole (Fig. 1a). Interestingly, the apical marker podocalyxin was polarised in detached cells, however, independently of the ezrin pole, localising to a PM area located distal to the nucleus (Fig. 1a), demonstrating that sc polarity is distinct from apical–basal polarity. Furthermore, in suspension the ezrin pole was not aligned with the nuclear–centrosomal axis, distinguishing it from uropod-like structures of amoeboid migrating cells^12,13^ (Supplementary Fig. 1b).

The PM is convoluted at the site of ezrin accumulation as demonstrated by correlative light and electron microscopy (CLEM) of SkMel2 cells expressing ezrin-GFP in suspension (Fig. 1b). The PM folds were larger than microvilli or shedded microvesicles and connected to the cell body, as confirmed by serial tomography (Fig. 1c, Supplementary Movie 1). Ribosomes, endoplasmic reticulum (ER) and mitochondria were identified and visualised inside the PM folds (Fig. 1c, d). Furthermore, an actin-rim was observed at the base of the pole structure (Fig. 1e), corroborating the phalloidin staining (Fig. 1a). Analysis of pole morphologies revealed two subpopulations of polarised cells, with cap-like or spot-like, sometimes protruding poles (Supplementary Fig. 1c, Supplementary Movies 2 and 3).

**Fig. 2:**
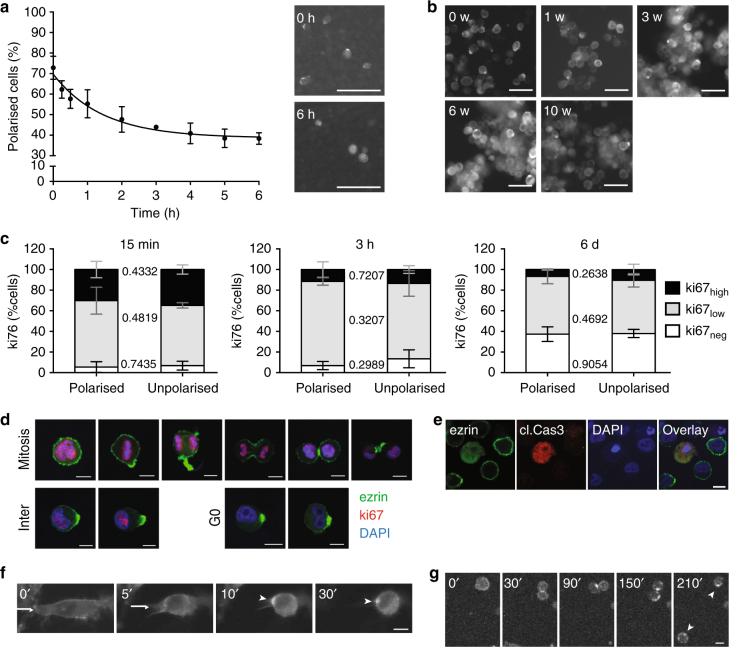
Kinetics and generation of the sc pole. **a** Polarity assays of SkMel2 cells in suspension expressing ezrin-GFP showing the decrease in fraction of polarised cells over time (6 h) in suspension (*n* = 6, mean ± SD). The line shows an exponential decay fit. **b** Fluorescence images of SkMel2 cells expressing ezrin-GFP maintained in suspension on poly-HEMA-coated plates for 10 weeks. Scale bars: 50 µm. **c** SkMel2 cells expressing ezrin-GFP maintained in suspension for 15 min, 3 h or 6 days were stained against ki67. Polarised and unpolarised cells within each population were graded by intensities into high, low and negative ki67expression. In all, 17–90 single cells were analysed per measurement (*n* = 3, mean ± SD, unpaired *t*-tests). **d** Representative images of SkMel2 cells expressing ezrin-GFP stained for ki67 and DAPI at different cell-cycle stages. Scale bars: 10 µm. **e** Representative images of SkMel2 cells expressing ezrin-GFP stained for cleaved Caspase 3 and DAPI. All cleaved Caspase 3-positive cells displayed cytoplasmic ezrin. Scale bar: 10 µm. **f**,** g** Floursecence timelapse imaging (minutes) of SkMel2 cells expressing ezrin-GFP during detachment (**f**) or cell division (**g**). The full sequences are shown in Supplementary Movies 5 and 9, respectively. Supplementary Movies 7 and 8 show further detaching cells. Arrows indicate retracting protrusions; arrowheads indicate polar ezrin accumulation. Scale bars: 10 µm

To assess the dynamics of sc polarisation, we performed polarity assays with detached SkMel2 cells maintained in suspension (Fig. 2a). The fraction of polarised cells, which is defined as the percentage of cells forming an ezrin pole, decreased with a *T*_1/2_ of 1 h. Even after 6 h of depolarisation, 39% of cells remained polarised (Fig. 2a). When maintained in suspension on a non-adhesive (poly-HEMA) substrate, most cells maintained their poles over hours, while only few cells displayed dynamic poles (Supplementary Movie 4). Polarised cells were detected throughout a period of 10 weeks (Fig. 2b). After 4 h in suspension, cells started to aggregate (Supplementary Fig. 1d). The distribution of cell-cycle stages as measured by ki67-positivity^14,15^ remained equal for polarised and unpolarised cells in suspension (Fig. 2c). Polarisation was observed among ki67^high^, ki67^low^ and ki67^neg^ cells (Fig. 2c, d), indicating that sc polarity is not specifically associated with a certain cell-cycle stage. Apoptotic cells always displayed cytoplasmic ezrin distribution (Fig. 2e). Imaging of detaching SkMel2 cells showed that the pole was frequently generated from a retracting protrusion where ezrin accumulated and remained as a stable spot upon cell rounding (Fig. 2f, Supplementary Movie 5), similar to ezrin accumulation in physiological processes such as morphological switch to amoeboid shape (Supplementary Movie 6) or tail retraction^11^. In cells with more than one retracting protrusion, multiple ezrin spots fused into one pole (Supplementary Movie 7). A minor population of cells developed the pole from a preformed ezrin-rich site at the flank of the cell (Supplementary Movie 8). In dividing cells, ezrin accumulated at the cleavage furrow^10^ (Fig. 2d, g) and remained polarised in daughter cells after division (Fig. 2g and Supplementary Movie 9). These data indicate that single tumour cells in liquid phase maintain a polarisation of ezrin, actin and the PM that constitutes a 'memory' of a previous polarised state, irrespective of the type of initial polarisation event.

### Sc polarity is observed in tumour cells from patients

**Fig. 3:**
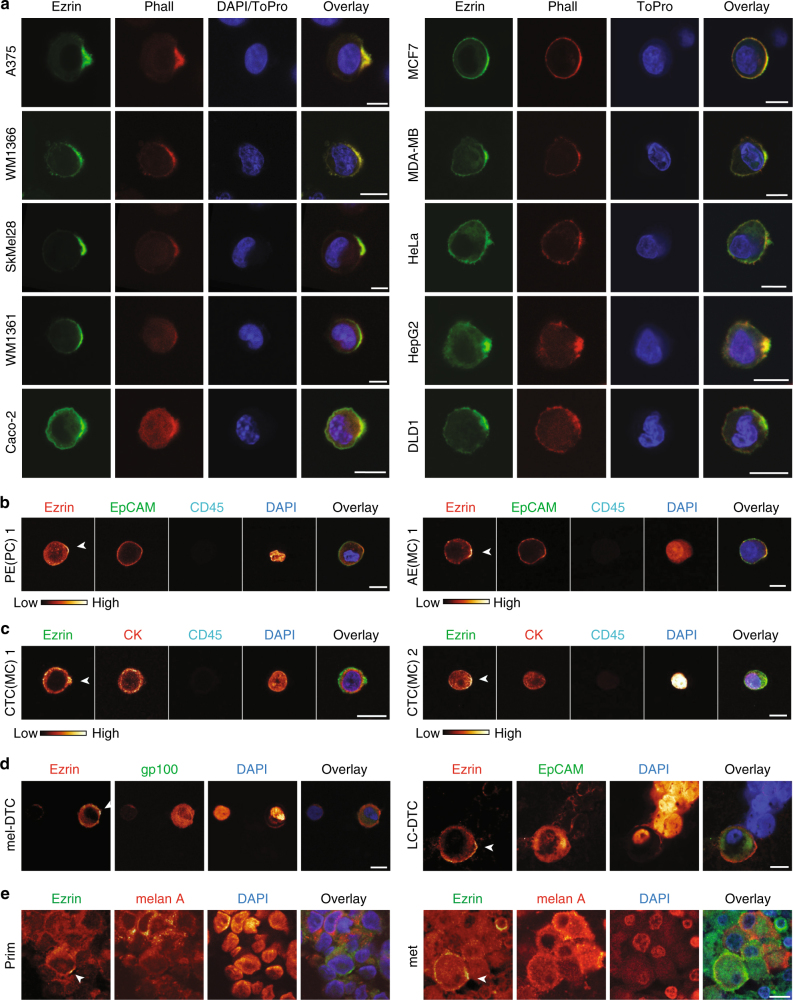
Sc polarity is observed in tumour cells from patients. **a** Representative images of tumour cells expressing ezrin-GFP (ezrin) stained with phalloidin and DAPI or TO-PRO3. Quantifications of polarisation in suspension are shown in Supplementary Table 1. **b** Cells from pleural effusion (PE) of a pancreatic carcinoma (PC) and from ascitic effusion (AE) of a breast cancer (MC mammary carcinoma) patient stained for ezrin, EpCAM, CD45 and DAPI. Control stainings for PE(PC) are shown in Supplementary Fig. 3a. Additional PE cells from MC patients are shown in Supplementary Fig. 2. **c** Circulating tumour cells (CTC) from blood of two MC patients stained for cytokeratin (CK), ezrin and DAPI. Control stainings for CTC(MC) 2 are shown in Supplementary Fig. 3b. Further CTCs are shown in Supplementary Fig. 2. Images of CTCs during Cell Search isolation and confirmation of CTC identity by single-cell array comparative hybridisation (aCGH) are shown in Supplementary Figs. 4 and 5. **d** Single, disseminated tumour cells from lymph nodes of melanoma (mel-DTC, left) or lung cancer (LC-DTC) patients stained for gp100 or EpCAM, ezrin and DAPI. Scale bars: 10 µm. Controls are shown in Supplementary Fig. 3c. **e** Paraffin samples of established human primary (left) or metastatic (right) melanoma stained for ezrin, melan A and DAPI. Scale bars: 10 µm. Arrowheads indicate sc poles

Polarisation of ezrin-GFP and F-actin was also observed in detached cells from different tumour entities, including melanoma, colorectal adenocarcinoma, cervical adenocarcinoma, hepatocellular carcinoma and breast invasive ductal carcinoma (Fig. 3a and Supplementary Table 1), demonstrating that sc polarity constitutes a general mechanism by which detached tumour cells of different entities maintain their polarity.

To ascertain whether our observations in cell lines also hold true for human specimens, we investigated tumour cells from liquid-phase biopsies of cancer patients for the presence of ezrin caps or spots (Supplementary Table 2). We observed polarisation of ezrin and CD44 in cells from pleural effusion (PE) or ascitic effusion (AE) of pancreatic and breast cancer patients (Fig. 3b, Supplementary Fig. 2 and Supplementary Movie 10) showing that sc polarity occurs in human patients. Additionally, we detected sc polarity in single circulating tumour cells (CTCs) isolated from peripheral blood of metastatic breast cancer patients (Fig. 3c, Supplementary Fig. 2 and Supplementary Movie 11) and in a cluster of CTCs from a small cell lung cancer (SCLC) patient (Supplementary Fig. 2c). Polarisation of ezrin, phalloidin, β1-Integrin and MCAM was observed in CTCs (Supplementary Fig. 2d–f). Malignant origin of CTCs was confirmed by single-cell array comparative hybridization^16^ (Supplementary Figs. 4 and 5). Ezrin polarisation was also confirmed in single, disseminated melanoma or non-SCLC cells in lymph node specimens of patients (Fig. 3d) and even observed in single, round melanoma cells within the tumour mass of established primary and metastatic melanomas (Fig. 3e), however, at a very low frequency (<0.1%). The fraction of polarised cells was increased (~1–20%) in cell lines derived from these tumours (Supplementary Fig. 3d) when cells were singularised in suspension, indicating that detachment favours sc polarity. Altogether, our data show that sc polarity occurs in different tumour entities in the liquid phase and in single cells at different steps of the metastatic cascade.

### Sc polarity is correlated with tumour metastasis

**Fig. 4:**
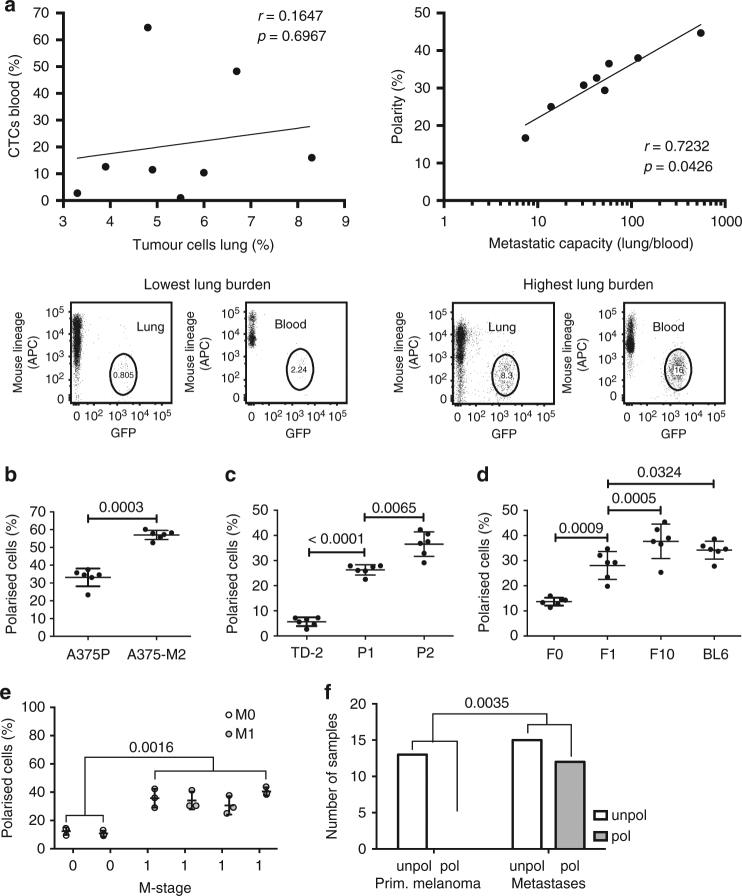
Sc polarity correlates with metastatic potential. **a** GFP+ CTCs isolated from MDA-MB-231-GFP tumour-bearing mice (Supplementary Fig. 6), quantified (as the percentage of circulating PBMCs) and analysed for sc polarity by phalloidin staining and imaging of actin polarisation. Lung tumour burden was assessed by the number of GFP+ cells/total lung cells. Number of CTCs is plotted against number of tumour cells in the lung (left) or fraction of polarised CTCs against logarithmised metastatic capacity (tumour cells in the lung normalised by tumour cells in the blood, middle) (Pearson’s correlation). Lower panel: representative density plots of mice with the lowest (left) and highest (right) tumour burden in the lung, showing 8000 events gated as intact and viable single cells by DAPI exclusion. **b** Fraction of polarised cells in suspension measured in parental A375P and the more metastatic daughter cell line A375-M2 expressing ezrin-GFP (*n* = 6, mean ± SD, paired *t*-test). **c** Fraction of polarised cells in suspension measured in parental TD and the more metastatic daughter cell lines P1 and P2 expressing ezrin-GFP (*n* = 6, mean ± SD, paired *t*-tests). **d** Fraction of polarised cells in suspension measured in parental B16-F0 and the more metastatic daughter cell lines B16-F1, B16-F10 and B16-BL6 expressing ezrin-GFP (*n* = 6, mean ± SD, paired *t*-tests). **e** Patient-derived ccRCC cell lines from M0 or M1 stage at early passages analysed in suspension for sc polarity by staining and imaging of actin polarisation. (*n* = 3, mean ± SD, repeated-measures ANOVA). **f** Patient-derived melanoma cell lines isolated either from primary melanoma (left) or metastases (right) analysed in suspension (spotted on a TMA) for fraction of polarised cells by staining and imaging of actin. Samples containing ≥3 polarised melanoma cells in a spot were graded as polarised (grey); samples containing 0–2 polarised melanoma cells were graded as unpolarised (white) (*n* = 13 for primary melanoma, *n* = 27 for metastases, Fisher’s exact test)

In order to investigate the role of sc polarity in metastasis in vivo, an MDA-MB-231 xenograft mouse model^17^ was used to isolate GFP-labelled CTCs, assess their polarisation state and measure the metastatic burden in the lung (Fig. 4a). The number of CTCs in the blood showed no clear correlation with the number of tumour cells in the lung. However, the log metastatic capacity of the CTCs, defined as number of tumour cells in the lung normalised by number of CTCs in the blood of each mouse, showed clear correlation with the fraction of polarised CTCs (Fig. 4a), indicating that increased sc polarity is associated with a higher metastatic activity of CTCs. Furthermore, sc polarity was significantly lower in parental A375 human melanoma (Fig. 4b), TD-2 mouse pancreatic ductal adenocarcinoma (Fig. 4c)^18^ or B16-F0 mouse melanoma (Fig. 4d) than in more metastatic daughter cell lines derived from A375^19^, TD-2^18^ or B16-F0^20^. Consistently, clear cell renal cell carcinoma (ccRCC) cell lines from patients with distant metastasis (M1) displayed an increased fraction of polarised cells as compared to cell lines from patients without distant metastasis (M0) (Fig. 4e). Moreover, melanoma cell lines derived from metastases also showed increased sc polarity as compared to cell lines derived from primary melanoma^21^ (Fig. 4f). Altogether, these data show a correlation between sc polarity and the cell-intrinsic metastatic potential of tumour cells in mouse models and human tumour cells.

### The sc pole is involved in early cell attachment

**Fig. 5:**
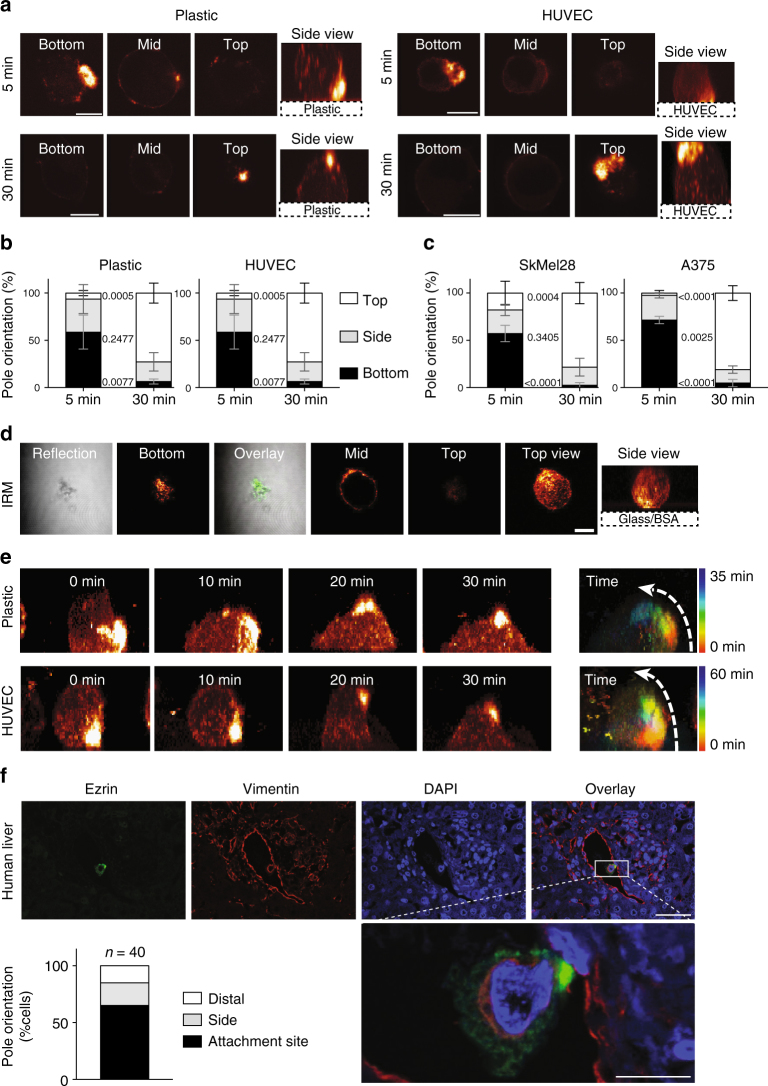
Orientation of the pole during attachment. **a** SkMel2 cells expressing ezrin-GFP, on top of plastic or HUVEC. Confocal sections through the bottom, centre (mid) and top of the cell and reconstructed side views (side) are shown. Scale bars: 10 µm. **b** Quantifications of the percentage of cells with their pole oriented towards the bottom (black), the side (grey) or the top (white) for cells treated as in **a**. More than 20 cells were analysed for each measurement (*n* = 3, mean ± SD, unpaired *t*-test). **c** Quantification of orientation of SkMel28 and A375 cells at 5 and 30 or 45 min after seeding on plastic as in **a**. More than 20 cells were analysed for each measurement (*n* = 4, mean ± SD, unpaired *t*-test). **d** Representative interference reflection microscopy (IRM) image of an SkMel2 cell expressing ezrin-GFP attached for 5 min to BSA-coated glass. The reflection image and confocal sections through the bottom, centre (mid) and top of the cell and reconstructed top and side views are shown. The overlay of the reflection image and the bottom section show co-localisation. Scale bar: 10 µm. **e** Live-cell imaging of SkMel2 cells settling onto a plastic surface (upper panel) or a layer of HUVEC (lower panel). Full series are shown in Supplementary Movies 13 and 14. Images show the side view reconstructed from confocal stacks. Rainbow colour-coding (right) shows the reorientation of the pole over time, indicated by a dashed arrow. **f** SkMel2 cell expressing ezrin-GFP attached to a vessel wall on a paraffin-section through a liver resectate stained for GFP (ezrin), vimentin and DAPI. Immunohistochemical stainings and further cells are shown in Supplementary Fig. 7. Scale bars: 50 µm (overview) and 10 µm (detail). In 12 different sections, the poles of 40 cells attached to vessels oriented towards the attachment site (black), the side (grey) or the distal side (white) were quantified

To measure the orientation of the pole during attachment, SkMel2 cells expressing ezrin-GFP were seeded onto plastic or a layer of human umbilical vein endothelial cells (HUVEC). Cells were fixed after 5 or 30 min (Fig. 5a) and non-attached cells were removed by gentle washing. The ezrin pole was predominantly oriented towards the substrate (plastic or HUVEC) at 5 min suggesting an active role of the pole in attachment. At 30 min after seeding, the pole shifted towards the distal side (Fig. 5a, b). This change in orientation was also confirmed in SkMel28 and A375 melanoma cells (Fig. 5c). Interference reflection microscopy (IRM) demonstrated close contact of the pole to the substrate at 5 min (Fig. 5d). Attachment with the pole and reorientation was also observed by confocal live-cell imaging (Fig. 5e, Supplementary Movies 12–14), showing persistence of the ezrin pole throughout the process. However, rapid reorientation of the pole towards the substrate upon attachment cannot be excluded. Unpolarised cells accumulated ezrin at the distal side upon attachment (Supplementary Movie 15), similar to a previously described moesin cap^9^. The finding that the early orientation towards the substrate was also observed on plastic or glass surfaces suggests that these initial interactions between sc pole and substrate are unspecific and not receptor-mediated.

We next investigated the polarisation of human melanoma cells during seeding in the human liver, one of the target sites for melanoma metastasis^22^, using an ex vivo liver perfusion system (Fig. 5f and Supplementary Fig. 7, K.Esser et al., manuscript in preparation). SkMel2 cells expressing ezrin-GFP were introduced into a human liver resectate maintained under intravenous perfusion. After injection of tumour cells, the liver was perfused for 5 min in order to remove non-attached cells before fixation. Distribution, attachment and orientation of retained melanoma cells were assessed by immunofluorescence and immunohistochemistry (Fig. 5f and Supplementary Fig. 7). Most melanoma cells were mechanically arrested in liver sinusoids and deformed (Supplementary Fig. 7b, c). In addition, single, round cells predominantly oriented with the pole towards the attachment site were identified in larger vessels (Fig. 5f and Supplementary Fig. 7d). Altogether, these experiments show that melanoma cells are initially oriented with the pole towards their attachment site in vitro as well as in the human liver ex vivo and that the pole is in close contact with the substrate, indicating that the pole is directly involved in early attachment.

### MCAM, Merlin and MLC regulate sc polarity and transmigration

**Fig. 6:**
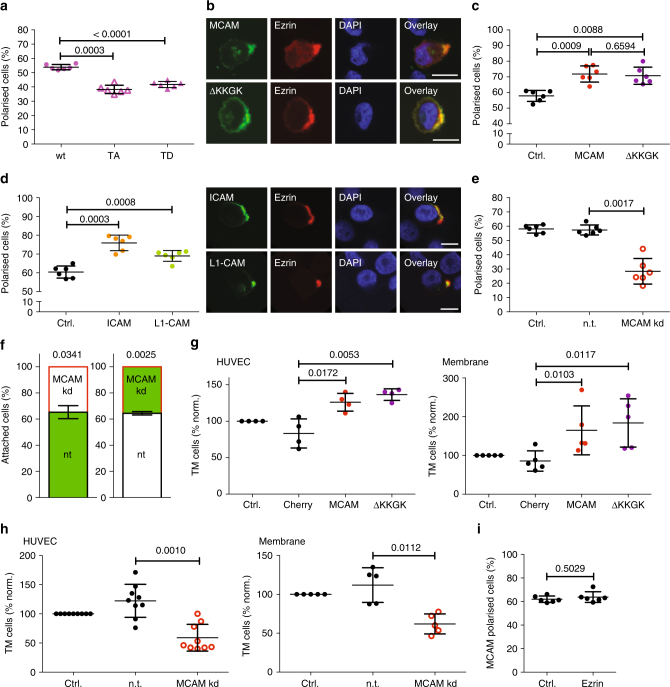
MCAM regulates sc polarity and transmigration. **a** Polarity assays of SkMel2 cells expressing GFP-labelled wild-type (wt) ezrin or ezrin TA or TD mutants (*n* = 6, paired *t*-test). Representative images are shown in Supplementary Fig. 8c. **b** Representative images of SkMel2 cells in suspension expressing MCAM-GFP (MCAM) or MCAMΔKKGK-GFP (ΔKKGK) and ezrin-mCherry (ezrin) stained with DAPI. Scale bars: 10 µm. **c** Polarity assays of SkMel2 cells expressing ezrin-GFP (ctrl.) together with MCAM-mCherry (MCAM) or MCAMΔKKGK-mCherry (ΔKKGK) (*n* = 6, paired *t*-tests). MCAM expression was evaluated by mCherry fluorescence. **d** Polarity assays and representative images of SkMel2 cells expressing ezrin-mCherry (ctrl.) with GFP-ICAM (ICAM) or YFP-L1-CAM (L1-CAM) (*n* = 6, paired *t*-test). Scale bars: 10 µm. **e** Polarity assays of SkMel2 cells expressing ezrin-GFP (ctrl.) with non-targeting (n.t.) or *MCAM*-targeting (MCAM kd) siRNAs (*n* = 6, paired *t*-test). MCAM expression is shown in Supplementary Fig. 8d. **f** Adhesion of SkMel2 cells transfected with non-targeting (n.t.) or *MCAM*-targeting (MCAM kd) siRNAs was measured simultaneously in flow chambers. Cells were either unlabelled (white) or labelled with CellTracker green (green). The number of attached cells was quantified after 30 min. In all, 70–290 cells were analysed per measurement (*n* = 3, one-sample *t*-test compared to 50). **g** Transmigration assays of SkMel2 cells expressing no construct (ctrl.), mCherry (Cherry), MCAM-mCherry (MCAM) or MCAMΔKKGK-mCherry (ΔKKGK) through HUVEC (left) or through membrane only (right) showing the number of transmigrated cells after 24 h normalised to ctrl. (*n* = 4 (left), *n* = 5 (right), paired *t*-tests). **h** Transmigration assays of SkMel2 cells expressing no siRNA (ctrl.), n.t. or *MCAM*-targeting (MCAM kd) siRNAs as described in **g**. (*n* = 9 (left), *n* = 5 (right), paired *t*-tests). **i** Polarity assays of SkMel2 cells expressing MCAM-mCherry (ctrl.) with ezrin-GFP showing the fraction of MCAM polarised cells (*n* = 6, mean ± SD, paired *t*-test). Ezrin expression was evaluated by GFP fluorescence. All data represented as mean ± SD

To investigate the regulation of sc polarity, we first analysed the effects of ezrin overexpression, knockdown (kd) or mutant ezrin on sc polarity. Kd or overexpression of ezrin showed no relevant effect on the fraction of polarised SkMel2 cells (Supplementary Fig. 8a, b). The phospho-mimetic ezrin-TD and the non-phosphorylable ezrin-TA mutants both displayed decreased fractions of polarised cells (Fig. 6a), indicating that ezrin phosphorylation turnover but not ezrin expression levels play a role in the regulation of the polar localisation of ezrin. However, secondary effects due to overexpression of ezrin or mutants cannot be excluded^23^. We therefore identified other cellular factors that affect sc polarity and investigated their effect on transmigration and experimental metastasis.

One of the proteins that co-localised with the ezrin pole was MCAM (Figs. 1a and 6b), which is a melanoma progression marker^24^ involved in adhesion, intracellular signaling and regulation of the rear of migrating cells^25–30^. In SkMel2 cells, overexpression of MCAM increased the fraction of polarised cells (Fig. 6c). This effect is not limited to MCAM, as overexpression of the cell adhesion molecules (CAMs) L1-CAM or ICAM also increased the fraction of polarised cells (Fig. 6d), although to a lesser extent. Consistently, kd of *MCAM* using small interfering RNAs (siRNAs) reduced the fraction of polarised cells (Fig. 6e). Ezrin was evenly localised and not decreased at the PM of *MCAM* kd cells (Supplementary Fig. 8e). *MCAM* kd also reduced adhesion of cells under flow conditions (Fig. 6f). In transwell assays, overexpression of MCAM enhanced transmigration through a layer of HUVEC (Fig. 6g), whereas *MCAM* kd reduced transmigration (Fig. 6h). Interestingly, these effects were also observed without HUVEC, indicating a cell-autonomous mechanism (Fig. 6g, h). A positive feedback mechanism between MCAM and ezrin has been proposed involving direct MCAM–ezrin interaction^26^. However, deletion of the ezrin-interaction motif KKGK in MCAM (MCAMΔKKGK)^26^ did not alter the subcellular distribution of MCAM (Fig. 6b) or revert the effect of MCAM overexpression on ezrin polarisation (Fig. 6c) and transmigration (Fig. 6g). Moreover, ezrin overexpression did not decisively increase the fraction of cells displaying polarised MCAM (Fig. 6i), indicating that the regulation of sc polarity by MCAM is independent of positive feedback or direct interactions between MCAM and ezrin.

**Fig. 7:**
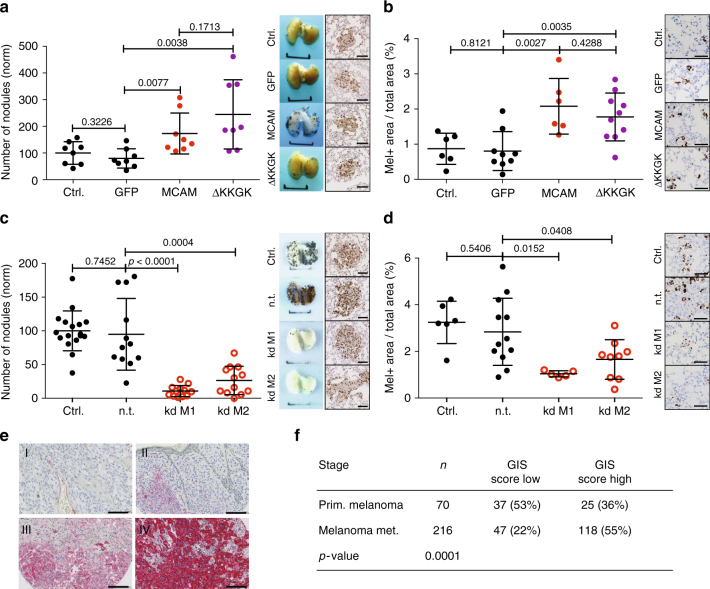
MCAM regulates metastatic seeding in vivo. **a** Experimental metastasis assays of B16-F0 cells expressing no construct (ctrl.), GFP, Mcam or McamΔKKGK (ΔKKGK). The number of nodules on the lung was quantified after 2 weeks (all values normalised to mean of control, *n* = 8, mean ± SD, unpaired *t*-tests). Representative macroscopies (scale bar: 1 cm) and ki67-stained histological images of tumours (scale bar: 100 µm) are shown to the right. Mcam expression, polarity, transmigration and growth assays are shown in Supplementary Fig. 9a–d. **b** Metastatic seeding assays of B16-F0 cells expressing no construct (ctrl.), GFP, Mcam or McamΔKKGK (ΔKKGK). Seeding to the lung after 30 min was assessed by quantification of melan A+area per total area on histological stainings from mouse lungs. Representative gp100 stainings are shown to the right. Scale bar: 50 µm. (*n* = 6–10, mean ± SD, unpaired *t*-tests). **c** Experimental metastasis assays of B16-F1 cells expressing no shRNA (ctrl.), n.t. or *Mcam*-targeting (MCAM kd M1, MCAM kd M2) shRNAs quantified as in **a** (all values normalised to mean of control, *n* = 12–17, mean ± SD, unpaired *t*-tests). Representative macroscopies (scale bar: 1 cm) and ki67-stained histological images of tumours (scale bar: 100 µm) are shown to the right. Mcam expression, polarity, transmigration and growth assays are shown in Supplementary Fig. 9e–h. **d **Metastatic seeding assays of B16-F1 cells expressing no shRNA (ctrl.), n.t. or *Mcam*-targeting (MCAM kd M1, MCAM kd M2) shRNAs performed and quantified as described in **b** (*n* = 5–11, mean ± SD, unpaired *t*-tests). Representative gp100 stainings are shown to the right. Scale bar: 50 µm. (mean ± SD, unpaired *t*-tests). **e** Representative images of MCAM-stained primary melanoma samples showing absence (I) or MCAM expression in <50% of tumour cells (II), melanoma lymph node metastasis showing MCAM expression in >50% (III) and brain metastases (IV) showing MCAM expression in >90% of melanoma cells. Scale bars: 100 µm. **f** Evaluation of MCAM expression in samples of human primary and metastatic melanoma described in Supplementary Table 3 (Pearson’s Chi-squared test)

In vivo, overexpression of wild-type (wt) Mcam as well as McamΔKKGK increased the number of experimental metastases (Fig. 7a) as well as early metastatic seeding (Fig. 7b) of B16-F0 mouse melanoma cells to mouse lungs. While overexpression of wt Mcam had no effect on tumour size, overexpression of McamΔKKGK reduced the size of experimental metastases (Supplementary Fig. 9c), indicating that the interaction between Mcam and ezrin plays no role in tumour seeding but in tumour growth. Consistently, kd of *Mcam* decreased experimental metastasis (Fig. 7c) as well as early metastatic seeding (Fig. 7d) of B16-F1 mouse melanoma cells, corroborating our in vitro observations. The effect of *Mcam* kd on long-term experimental metastasis (Fig. 7c) was more pronounced than the effect on metastatic seeding (Fig. 7d), suggesting that MCAM also plays a role during later steps of metastasis following metastatic seeding. As in SkMel2 cells, polarisation and transmigration were induced by Mcam or McamΔKKGK overexpression and reduced by *Mcam* kd in the mouse cell lines (Supplementary Fig. 9a, b, e, f). The size of tumour nodules was not affected by *Mcam* kd (Supplementary Fig. 9g). Analysis of MCAM expression in human melanoma tissue microarrays (TMAs) revealed that MCAM was more frequently expressed in metastases as compared to primary melanomas (*p* = 0.0001, Fig. 7e, f and Supplementary Table 3) in agreement with previous findings^31^. Altogether, these results demonstrate that MCAM positively regulates sc polarity, transmigration and metastasis and reveal an additional, cell-autonomous mechanism by which overexpression of CAMs can favour metastasis.

**Fig. 8:**
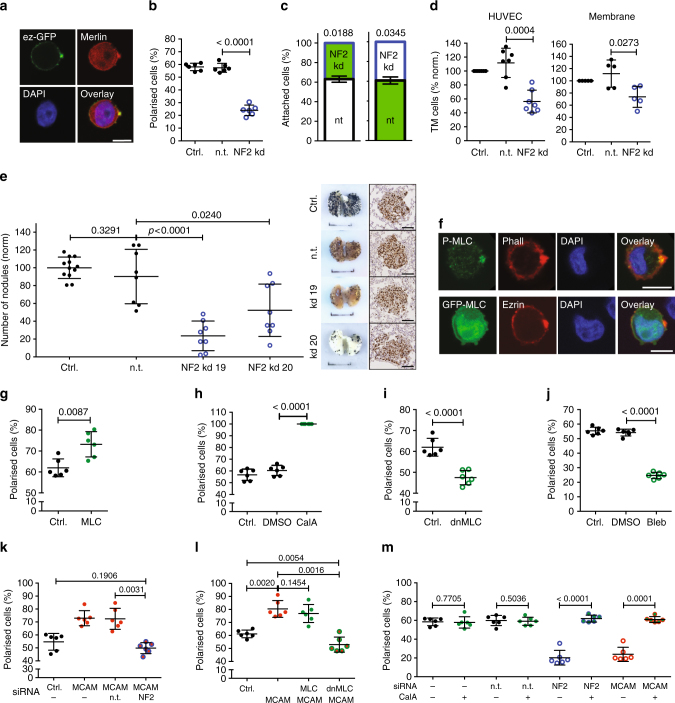
Regulation of sc polarity by Merlin and MLC. **a** Representative image of an SkMel2 cell expressing ezrin-GFP stained for merlin and DAPI. Scale bar: 10 µm. **b** Polarity assays of SkMel2 cells expressing ezrin-GFP (ctrl.) and non-targeting (n.t.) or *NF2*-targeting (NF2 kd) siRNAs (*n* = 6, mean ± SD, paired *t*-test). Merlin expression and additional NF2 kd cell lines are shown in Supplementary Fig. 11. **c** Adhesion of n.t. or NF2 kd SkMel2 cells was measured simultaneously in flow chambers. Cells were either unlabelled (white) or labelled with CellTracker green (green). The number of attached cells was measured after 30 min. In all, 60–260 cells were analysed per measurement (*n* = 3, mean ± SD, one-sample *t*-test compared to 50). **d** Transmigration assays of SkMel2 kd cells as in **b** through HUVEC or membrane showing the number of transmigrated cells after 24 h normalised to control (*n* = 7 (left), *n* = 5 (right), mean ± SD, paired *t*-tests). Additional NF2 kd cell lines are shown in Supplementary Fig. 11. **e** Experimental metastasis assays of B16-F1 cells expressing no shRNA (ctrl.), n.t. or *Nf2*-targeting (NF2 kd19, NF2 kd20) shRNAs quantified by the number of nodules on the lung after 2 weeks (values normalised to mean of control, *n* = 8–12, mean ± SD, unpaired *t*-tests) and representative macroscopies (scale bars: 1 cm) and ki67-stained histological images of tumours (scale bars: 100 µm) (right). Polarity, transmigration, Merlin expression and growth assays are shown in Supplementary Fig. 11. **f** Representative images of SkMel2 cells stained for P-MLC and phalloidin or transfected with GFP-MLC and ezrin-mCherry stained with DAPI. Scale bars: 10 µm. **g**–**m** Polarity assays of SkMel2 cells, expressing ezrin-mCherry (ctrl.) and GFP-MLC (**g**), expressing ezrin-GFP treated with 50 nM calyculin A (**h**), expressing ezrin-mCherry (ctrl.) with GFP-dnMLC (**i**), expressing ezrin-GFP treated with 2.5 µM blebbistatin (Bleb, **j**), coexpressing ezrin-GFP (ctrl.), MCAM-mCherry (MCAM) and n.t. or *NF2*-targeting (NF kd) siRNAs (**k**), coexpressing ezrin-mCherry (ctrl.) with MCAM-CFP (MCAM) and MLC-GFP or dnMLC-GFP (**l**) or expressing ezrin-GFP (ctrl.) with n.t., NF kd or MCAM kd siRNAs untreated or treated with 10 nM calyculin A (CalA) (**m**) (*n* = 6, mean ± SD, paired *t*-tests). Supplementary Fig. 13 shows representative images and expression controls

Merlin, the product of the tumour--suppressor gene neurofibromatosis type II (*NF2*), is a regulator of sc polarity during cell division^10^. In detached SkMel2 cells, endogenous merlin was mostly cytoplasmic, but weak staining at the PM and pole was also observed (Fig. 8a). In polarity assays, kd of *NF2* decreased the fraction of polarised cells (Fig. 8b). Ezrin was evenly distributed and not decreased at the PM in *NF2* kd cells (Supplementary Fig. 11b). Consistently, *NF2* kd also reduced adhesion under flow (Fig. 8c) and transmigration of SkMel2 cells through HUVEC or membrane (Fig. 8d). This result contrasts previous findings in cell lines, where kd of *NF2* resulted in increased transmigration^32^. We therefore performed additional polarity and transmigration assays in three different melanoma cell lines A375, SkMel28 and WM1366 with *NF2* kd (Supplementary Fig. 11c), demonstrating that the effect of loss of merlin is cell type-dependent. Importantly, the effect on transmigration in each of the cell lines was correlated with the effect on sc polarity (Supplementary Fig. 11c). B16-F1 mouse melanoma cells with kd of *Nf2* displayed significantly decreased experimental metastasis in vivo (Fig. 8e), correlating with reduced sc polarity and transmigration (Supplementary Fig. 11d, e). *Nf2* kd had no effect on tumour size (Supplementary Fig. 11f). Our data indicate that merlin, while being a negative regulator of tumourigenesis^33^, can be a positive regulator of transmigration and metastasis of tumour cells.

Myosin light chain (MLC) was previously shown to regulate polarisation of amoeboid migrating cells^34,35^. In SkMel2 cells, phosphorylated MLC (P-MLC) strongly accumulated at the pole (Fig. 8f), while GFP-MLC was distributed cytoplasmic, demonstrating local activation of MLC at the pole. Overexpression of wt MLC (Fig. 8g) or the increase of phosphorylated MLC by 50 nM calyculin A (Fig. 8h and Supplementary Fig. 13a) enhanced sc polarity, whereas overexpression of a dominant-negative (dn) MLC mutant (dnMLC, Fig. 8i) or the decrease of MLC activity by blebbistatin (Fig. 8j and Supplementary Fig. 13b) reduced sc polarity in SkMel2 cells.

We next investigated the connections between merlin, MCAM and MLC (Fig. 8k–m). Reduction of merlin levels in SkMel2 cells overexpressing MCAM reverted the fraction of polarised cells to control levels (Fig. 8k), indicating that merlin is required for the polarity-inducing effect of MCAM. Co-expression of wt MLC together with MCAM did not further increase sc polarity as compared to MCAM expression alone, but co-expression of dnMLC decreased the fraction of polarised cells to control levels (Fig. 8l), showing that MLC activity is required for the polarity-inducing effect of MCAM. Furthermore, activation of MLC by 10 nM calyculin A had no relevant effect on control cells but reverted sc polarity in *NF2* and *MCAM* kd cells to control levels (Fig. 8m and Supplementary Fig. 13c). We therefore conclude that phosphorylation of MLC is a major regulatory mechanism for sc polarity in liquid phase.

### Generic depolarisation decreases transmigration and seeding in vivo

**Fig. 9:**
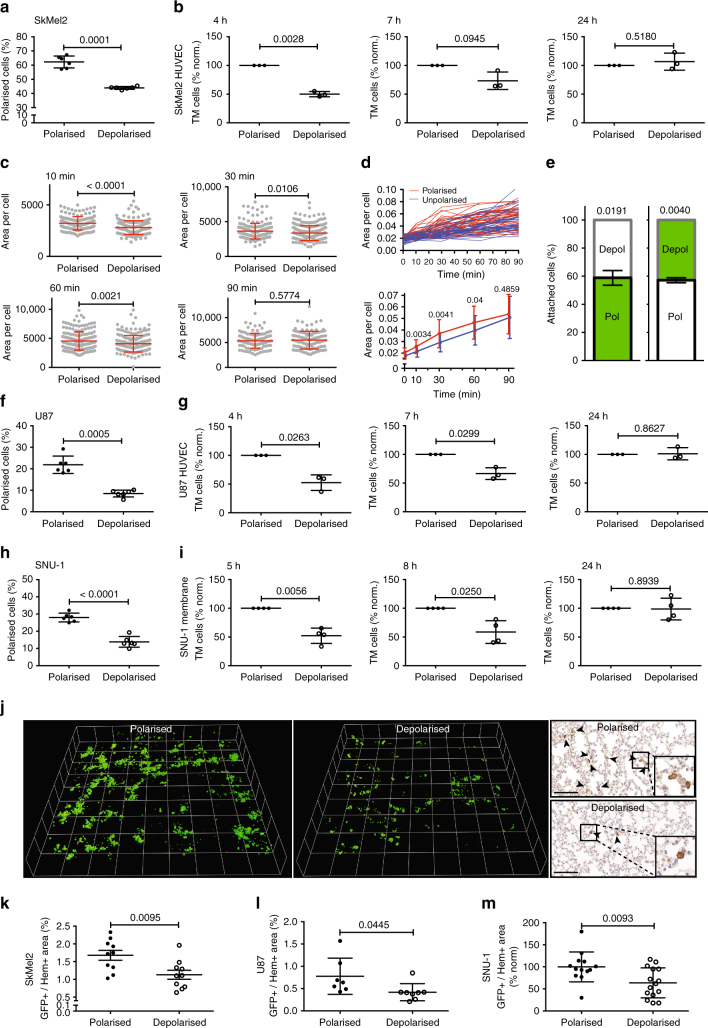
Generic depolarisation decreases transmigration and seeding. **a** Polarity assays from 30 min (polarised) and 3 h (depolarised) time points from Fig. 2a (*n* = 6, paired *t*-test). **b** Transmigration assays of polarised (30 min) and depolarised (3 h) SkMel2 cells through HUVEC after 4, 7 or 24 h (*n* = 3, one-sample *t*-tests compared to 100). Further transmigration assays are shown in Supplementary Fig. 15f, g. **c** Adhesion assays of polarised (30 min) and depolarised (3 h) ezrin-GFP-expressing SkMel2 cells settling onto plastic. The area per cell was measured at the indicated times in >110 cells from at least 3 independent experiments (unpaired *t*-tests). **d** Individual (top) and averaged (bottom) adhesion kinetics of single polarised (*n* = 48, red) and unpolarised (*n* = 30, blue) ezrin-GFP-expressing SkMel2 cells within one, untreated population. Polarisation prior to adhesion was assessed at time point 0. The area per cell was measured at the indicated times (unpaired *t*-tests). **e** Adhesion of polarised and depolarised SkMel2 cells measured simultaneously in flow chambers after 30 min. Cells were unlabelled (white) or labelled with CellTracker green (green). In all, 70–380 cells were analysed per measurement (*n* = 4, one-sample *t*-test compared to 50). **f**, **h** Polarity assays of ezrin-GFP-expressing U87 (**f**) and SNU-1 (**h**) cells at 30 min (polarised) and 3 h (depolarised) time points (*n* = 6, paired *t*-test). **g**, **i** Transmigration assays of polarised (30 min) and depolarised (3 h) U87 cells (**g**) through HUVEC after 4, 7 or 24 h and of SNU-1 cells (**i**) after 5, 8 or 24 h through membrane (*n* = 3 (**g**), *n* = 4 (**i**), one-sample *t*-tests compared to 100). **j** 3D reconstruction of GFP stainings from 30 serial 2 µm sections (right) of mouse lungs 30 min after injection with polarised or depolarised ezrin-GFP-expressing SkMel2 cells (Supplementary Movies 17 and 18). Grid length: 100 µm. Scale bars: 100 µm, arrowheads: GFP-positive cells. **k**–**m** Quantification of GFP-positive SkMel2 (**k**), U87 (**l**) and SNU-1 (**m**) cells from histological slides of mouse lungs as shown in **j** and Supplementary Fig. 17c, f by GFP-positive area per haematoxylin-positive area (*n* = 10 (**k**), *n* = 7–8 (**l**), *n* = 13–15 (**m**), unpaired *t*-tests, normalised to the average of 'polarised' in **m**). Further quantifications are shown in Supplementary Fig. 18. All data are represented as mean ± SD

As additional effects of MCAM or Merlin levels on transmigration and metastasis cannot be excluded^27,32^, we investigated the effect of generic depolarisation (without genetic or chemical manipulation) on transmigration and in vivo seeding of melanoma cells into the lung. SkMel2 cells were depolarised by incubation in suspension for 3 h, which reduced the fraction of polarised cells by 30% compared to 30 min in suspension (Fig. 9a). Depolarisation had no decisive effect on cell viability, apoptosis or growth (Supplementary Fig. 15a–c) or on the expression of PM receptors at the cell surface (Supplementary Fig. 15d). Using polarised and depolarised cells from the same preparation, transmigration assays were performed either through HUVEC (Fig. 9b) or through membranes (Supplementary Fig. 15f, g). Cells depolarised prior to seeding showed strongly reduced transmigration after 4 h and slightly reduced transmigration after 7 h compared to polarised cells (Fig. 9b and Supplementary Fig. 15f), demonstrating that the state of polarisation prior to seeding affects transmigration. The experiment was also extended to 24 h of transmigration time to allow seeded cells to (re-) polarise upon attachment to the substrate^9^ (Supplementary Movie 15). After 24 h, no clear difference between polarised and depolarised cells was observed (Fig. 9c and Supplementary Fig. 15g), showing that generic depolarisation does not relevantly affect the migratory capacity of cells. We therefore investigated whether sc polarity affects adhesion of cells (Fig. 9c, d). Adhesion of depolarised cells was delayed as compared to polarised cells up to 60 min after seeding (Fig. 9c). Furthermore, adhesion of single, unpolarised cells was delayed over the same time course compared to single, polarised cells within the same, untreated population of cells (Fig. 9d and Supplementary Movie 16). However, after 90 min, polarised and depolarised cells had adhered to the same extent. Under flow conditions, the slower adhesion kinetics of depolarised cells led to a reduced number of attached/adhered cells compared to polarised cells (Fig. 9e). The transient delay in adhesion over time (Fig. 9c, d) was consistent with the effect of generic depolarisation on transmigration (Fig. 9b). The transient, transmigration-reducing effect of generic depolarisation was also observed in U87 glioblastoma^36,37^ (Fig. 9f, g) and semi-adherent SNU-1 stomach carcinoma cells^38^ (Fig. 9h, i), which can both grow in adherent as well as in suspension phase. Depolarisation had no relevant effect on cell viability, apoptosis, growth or expression of PM receptors at the cell surface of U87 or SNU-1 cells (Supplementary Fig. 17).

To investigate the effect of generic depolarisation on seeding of cells into the mouse lung in vivo, polarised (30 min in suspension) and depolarised (3 h in suspension) SkMel2 cells from the same preparation expressing ezrin-GFP were injected into the tail vein of mice and the number of GFP^+^ cells was quantified in the lung after 30 min (Fig. 9j, k). Histological images and a three-dimensional (3D) reconstruction using step-cuts from lung tissue (Supplementary Movies 17 and 18) demonstrated that the seeding of cells was higher in the polarised fraction than in the depolarised fraction (Fig. 9j). The amount of GFP^+^ cells was quantified by three different methods (Fig. 9k and Supplementary Fig. 18a) to correct for effects of differences in tissue density. All three quantifications revealed a significantly decreased number of seeded cells from the depolarised fraction as compared to the polarised fraction (Fig. 9k and Supplementary Fig. 18a). The same effect of generic depolarisation on seeding into the lung was observed using U87 or SNU-1 cells (Fig. 9l, m and Supplementary Fig. 18b, c). Altogether, these experiments show that sc polarisation prior to attachment directly advances adhesion, transmigration and in vivo seeding of melanoma, glioblastoma and gastric cancer cells.

### Sc polarity affects attachment, adhesion and metastatic seeding

**Fig. 10:**
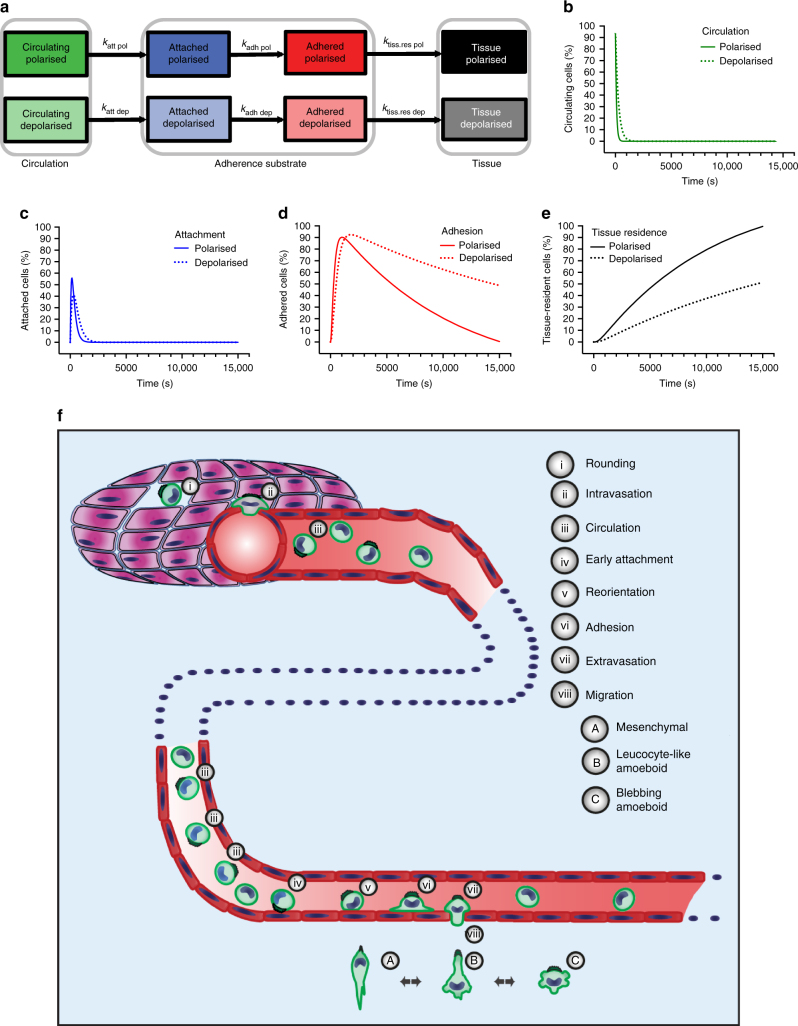
Model of the effects of sc polarity on metastatic seeding. **a** Scheme of the compartmental model generated from data shown in Fig. 9d, e, k and Supplementary Fig. 18a. Transfer rates for polarised (pol) and depolarised (dep) cells defined as attachment rate (*k*_att_), adhesion rate (*k*_adh_) and tissue residence rate (*k*_tiss.res_) are shown in Supplementary Figure 19b. **b** In silico simulation of the decrease of polarised and depolarised cells in the circulation compartment showing that polarised cells leave circulation faster than depolarised cells due to increased attachment and adhesion rates. The number of circulating cells rapidly decreases to 0 as the model does not account for recirculation due to circulatory pressure. **c** Simulation of attachment of polarised and depolarised cells to substrate. The higher attachment rate of polarised cells leads to a higher peak population as compared to depolarised cells. The rapid decrease in the number of attached cells is a result of the cells adhering and thus leaving the attachment compartment and entering the adhesion compartment. **d** Simulation of adhesion of polarised and depolarised cells to substrate, showing faster adhesion of polarised cells as compared to depolarised cells. The decrease in the number of adhered cells is a result of the cells extravasating and thus leaving the adhesion compartment and entering the tissue residence compartment. **e** Simulation of tissue residence of polarised and depolarised cells, showing decreased tissue residence and thus metastatic seeding of depolarised cells mainly due to delayed attachment and adhesion. **f** Model for possible roles of sc polarity during metastasis. Upon dedifferentiation, detachment and rounding, single cells in the primary tumour form an ezrin-rich pole (i). This pre-polarisation may favour intravasation (ii). During the liquid phase (iii) in blood, lymph or ascites fluid, tumour cells maintain their pole, which can facilitate their attachment by unspecific interaction (iv). Pre-polarisation advances reorientation (v) and transition of the pole into a distal cap (vi). During transmigration (vii) and interstitial migration (viii), the pole establishes the cell rear in all migratory phenotypes

In order to investigate which steps of early metastatic seeding are affected by sc polarity, a basic compartmental model was generated (Fig. 10a and Supplementary Fig. 19) comprising circulation, attachment, adherence and tissue residence of unmixed populations of 100% polarised or 100% depolarised cells using parameters for transfer rates between compartments measured for mixed populations of polarised and depolarised cells in Fig. 9d, e, k and Supplementary Fig. 18a.

This model was used to simulate the individual steps of early metastatic seeding and assess the differences in attachment, adhesion and tissue residence between polarised and depolarised cells (Fig. 10c–e and Supplementary Fig. 19). The model predicts an approximately 1.3-fold higher adherence rate for polarised cells (3.95%/s) as compared to depolarised cells (3.02%/s), which is in agreement with our experimental data on adhesion kinetics (Fig. 9d). Furthermore, the model predicts a three-fold attachment rate for polarised cells (10.74%/s) compared to depolarised cells (3.81%/s) as suggested by our data showing attachment of cells via the sc pole (Fig. 5). The predicted tissue residence rate of polarised cells (0.06%/s) is also slightly enhanced as compared to depolarised cells (0.05%/s) but considerably lower than attachment and adhesion rates and therefore contributing to the difference in tissue residence only to a minor extent. Simulation of the full chain of events taking all compartments into account demonstrates that a slight delay in attachment (Fig. 10c) and adhesion (Fig. 10d) of depolarised cells results in considerably decreased tissue residence (Fig. 10e) thus predicting a strong effect of sc polarity on metastatic seeding as observed in our in vivo experiments with mixed populations (Fig. 9k–l).

In summary, the model allows us to confirm our hypothesis that sc polarity increases attachment and adhesion rates (and to a minor extent tissue residence rate), leading to enhanced tissue residence of polarised cells.

## Discussion

We have performed a comprehensive investigation of sc polarity in detached tumour cells and its role in metastasis. We define sc polarity as a type of polarity that cells adopt in liquid phase without directional stimulus in vitro and in vivo. Sc polarity is characterized by a round morphology of cells and formation of a pole containing ezrin, F-actin, P-MLC, PIP_2_, CAMs, integrins and PM folding. Despite distinct functions, morphologies and origins, the molecular composition of the sc pole overlaps with that of uropod-like structures^12,13^, cell capping during cell division^10^, adhesion^9^ or leucocyte capping^11^, invadosomes^39^, the immunological synapse (IS)^40^ or the distal pole of IS-forming cells^41^, which might favour the transition between the sc pole and such structures. While cells forming such structures are fully polarised by cell–cell attachment, cell–matrix attachment or migration and centrosome positioning, cells displaying sc polarity in liquid phase show no alignment of the pole with the nuclear–centrosomal axis. We propose that the sc pole constitutes a basic type of polarity of PM, linker proteins and actin cytoskeleton that cells maintain during phases of detachment independent of the initial polarisation type. In circulation, cells may acquire sc polarity by further mechanisms in addition to detachment or cell division, such as interaction with immune cells or platelets^42^ or confinement^34,35^ by mechanical entrapment. Sc polarity might furthermore reflect the tendency of metastasising cells to adapt their polarity to changing environmental requirements during the metastatic process and thus enhance their polarisation plasticity. Sc polarity of tumour cells was observed not only in cancer patients in the liquid phase (CTCs, PE and AE cells) but also at other steps of the metastatic process and might favour metastasis at different steps during tumour progression by maintaining a generic type of polarity (Fig. 10f).

Our in vitro, in vivo and in silico data suggest that sc polarity affects transmigration and metastasis by two distinct mechanisms, favouring early attachment by unspecific interaction with substrate and advancing adhesion by 'pre-polarisation'. First, local accumulation of PM rich in highly glycosylated proteins may lead to a high tendency for unspecific interaction of the sc pole with any substrate and thereby enhance early attachment as suggested by the preferential orientation of the pole towards the substrate (Fig. 5). Second, pre-polarisation of the PM and actin cytoskeleton might advance the formation of a distal cap^9^ and thereby favour adhesion. During the metastatic process in vivo, these two effects of sc polarity could lead to increased vascular retention and accelerated adhesion of CTCs.

The extent of sc polarity correlated with metastatic properties of human tumour cell lines as well as CTCs in a mouse model. Our findings agree with previous concepts^43,44^ suggesting that not only quantity but rather quality of CTCs and disseminated tumour cells influence the chance of successful metastatic colonisation and that sc polarity may constitute such a metastatic quality of CTCs. The therapeutic potential of inhibition of sc polarity to target cancer metastasis will need to be evaluated in clinical studies. Research into molecular determinants involved in regulation of sc polarity will provide further insight into the underlying cellular mechanisms.

## Methods

### Definition of sc polarisation

Throughout this study, a sc pole was defined as a single, continuous PM area encompassing less than half of the circumference of the cell image enriched in ezrin (or phalloidin) fluorescence by two-fold or higher as compared to the remaining area of the PM.

### Cell culture

Melanoma cell lines were kindly provided by Professor R. Marais (CRUK, Manchester) and Professor C. Marshall (ICR, London) with permission from Professor M. Herlyn (Wistar Institute, Philadelphia), Caco-2 and DLD-1 by Dr. K.-P. Janssen (Klinikum rechts der Isar, Munich), MDA-MB-231 and MCF-7 by Professor R. Faessler (MPI Munich), U87 (ATCC-HTB14)^45^ by Dr. S. Stangl (TU Munich) and SNU-1 by Professor M. Gerhard (TU Munich). Cell lines were not specifically authenticated. U87 and TD-2 cells were maintained in RPMI (Gibco); all other tumour cell lines in Dulbecco’s modified Eagle’s medium (DMEM; Gibco) with Glutamax and 10% foetal calf serum (FCS, Gibco), 100 IU/ml penicillin and 100 µg/ml streptomycin (Gibco). HUVEC were maintained in medium 199 (Sigma), with 20% FCS, 2 mM ultraglutamine (Lonza), 25 IU/ml penicillin and 25 µg/ml streptomycin (Gibco), 62.5 µg/ml Heparin (Sigma) and 30 µg/ml endothelial growth factor supplement (Sigma). SkMel2 cells stably expressing ezrin-GFP were fluorescence-activated cell sorted (FACS) for high green fluorescence and maintained in selection medium with 1 mg/ml G418/geneticin (Sigma). B16-F0 cells stably transfected with pEpi-GFP, pEpi-MCAM or pEpiMCAM-ΔKKGK were maintained in selection medium containing 5 µg/ml blasticidin (Applichem). B16-F1 cells stably transduced with short hairpin RNAs (shRNAs) were maintained in selection medium containing 625 ng/ml puromycin (Calbiochem). To image amoeboid morphology, cells were grown on top of a thick layer of 1.7 mg/ml bovine collagen I (Gibco A10644-01). Cell lines were routinely tested for mycoplasma infection using the PlasmoTest Kit (InvivoGen). All cells were split 1 day before use. Transfections with plasmids and/or siRNAs were performed using Lipofectamine 2000 or 3000 (Thermo Fisher Sci.); transductions with viral particles were performed according to the manufacturer’s protocol. Growth and viability of polarised and depolarised fractions of SkMel2, U87 and SNU-1 cells was assessed by (i) trypan blue staining and counting (ViCell XR, Beckman Coulter), (ii) plating of cells for 24 h, fixation in 4% formaldehyde, staining with 4,6-diamidino-2-phenylindole (DAPI), imaging of nuclei and counting using ImageJ^46^, (iii) AnnexinV staining (BD), (iv) live-dead staining using EMA (Life Technologies) and (v) LDH assay (Cytotoxicity Detection Kit, LDH, Roche) and (vi) soft agar colony-formation assay (CytoSelect 96-Well Cell Transformation Assay, Cell Biolabs, Inc.). For aggregation assays, cells were stained with 1 µM Cell Tracker Green CMFDA (Life Technologies) for 30 min, recovered in DMEM with 10% FCS for 24 h, detached with Versene (EDTA, Lonza) and fixed in suspension after the indicated time. Aggregation (direct contact of 2, 3 or >3 cells) was quantified from 20× widefield fluorescence images of fixed suspension cells. Growth of B16 cells was assessed using the Cell Proliferation Kit II (XTT, Roche). All *n* values represent biological replicates.

### Establishment and detachment of ccRCC cell lines

Primary clear cell renal cancer-derived cell lines were established from tumour resection specimens of ccRCC patients. Primary xenografts were established by implanting tumour pieces of 1–2 mm^3^ into the renal capsule of NOD.Cg-Prkdcscid Il2rgtm1Wjl (NSG) mice bred in the animal facility of the German Cancer Research Center. Primary xenografts were resected at a volume of approximately 1 cm^3^, tumour pieces were minced, dissociated by incubation with 1 μg/ml collagenase IV and DNase for 1 h at 37 °C and filtered through a 100- and 70-µm mesh. In all, 5 × 10^6^ cells were seeded in T75 flasks in serum-free medium. Suspension and adherent cultures were maintained at 37 °C and 5% CO_2_. Established KIKA lines^47^ were authenticated (Multiplexion) and tested for mycoplasma contaminations. After separation with StemPro Accutase (Gibco), cells were resuspended in CO_2_-independent medium (Gibco) supplemented with 1% bovine serum albumin (BSA), incubated 1 h at 37 °C in a tube rotator and fixed in 4% paraformaldehyde (PFA).

### Adhesion assays

SkMel2 cells expressing ezrin-GFP were detached for 10 min using Versene/EDTA, (Lonza) and incubated in DMEM for 30 min. Cells were seeded onto 24-well cell culture dishes, fixed with 4% formaldehyde after 10, 30, 60 or 90 min and imaged using a 20× objective on an Olympus CKX41 with an Olympus XM10 camera using the CellSens Standard software (Olympus). The area per cell was measured using ImageJ^46^. Adhesion under flow was measured using CellASIC ONIX microfluidic plates (EMD Millipore) using the capillary mode according to the manufacturer’s instructions. To compare two conditions, cells were either unstained or stained with 1 µM Cell Tracker Green CMFDA (Life Technologies) for 30 min and recovered in DMEM with 10% FCS for 24 h. A mix of 0.5 × 10^5^ stained and 0.5 × 10^5^ unstained cells were loaded in the flow chamber for 5 min, maintained under flow in DMEM for 30 min and fixed after 30 min in 4% formaldehyde. Stained and unstained cells were quantified from brightfield and fluorescence images. In all, 2 × 10^4^ cells of the mix were seeded on 24-well plates and fixed after 24 h to normalise for exact cell numbers. For adhesion of single cells, cells were seeded on chamber slides (µ-Slide 8 Well, ibiTreat, Ibidi) and images of 30 µm stacks were recorded every minute on a Leica TCS SP8 confocal microscope using a 10× objective and 2× zoom. Polarisation was assessed on the first stack. The area of each cell after 0, 10, 30, 60 and 90 min was quantified using ImageJ^46^ from projections of stacks. All *n* values represent biological replicates.

### Polarity assays

SkMel2, B16 and TD-2 cells were detached using Versene/EDTA; cells in Supplementary Table 1 were detached or singularised in suspension using Trypsin. Resuspended cells were incubated in DMEM for 30 min, fixed in suspension with 4% formaldehyde, washed with phosphate-buffered saline (PBS) and imaged in 96-well plates using a 10× or 20× objective on an Olympus CKX41 with an Olympus XM10 camera using the CellSens Standard software (Olympus). B16 cells in Supplementary Fig. 9a, 9e and 11d were fixed in suspension, mounted on a glass slide and imaged on a Leica SP5 confocal microscope using a 20× objective. The fraction of cells with a polarised cap or spot per total round cells was counted manually, blinded. For each experiment, images of at least five random areas were acquired and at least 100 cells were analysed. All *n* values represent biological replicates.

### Transmigration assays

Transmigration was measured in transwell dishes (Corning-Costar, Lowell MA, 8 µm pore size, 6.5 mm diameter). Inserts were either used uncoated or coated with 0.1% gelatine (Sigma) for 30 min and 3.5 × 10^4^ HUVEC (Cellworks, ZHC2301) for 3 days. For transmigration through HUVEC, cells were stained with 1 µM (SkMel2) or 10 µM (B16) Cell Tracker Green CMFDA (Life Technologies) for 30 min and recovered in DMEM with 10% FCS for 3 h (SkMel2) or 12 h (B16). Cells were detached using Versene and incubated in DMEM or HUVEC medium for 30 min. In all, 5 × 10^4^ SkMel2 or B16 or 2 × 10^5^ U87 cells were seeded on top of HUVEC, 10^6^ (SkMel) or 5 × 10^5^ (SNU-1) on the transwell insert, incubated as indicated and fixed with 4% formaldehyde. Cells on the top of the membrane were removed, transmigrated cells were stained with DAPI and membranes were mounted on a glass slide using Mowiol (Merck). Seven random images were obtained of DAPI (for transmigration through membrane only) or Cell Tracker Green (for transmigration through HUVEC) using a 10× objective on an Olympus CKX41 with an Olympus XM10 camera using the CellSens Standard software (Olympus). Cell numbers were quantified using ImageJ^46^. All *n* values represent biological replicates performed as technical duplicates and averaged; all experiments were normalised to an untransfected control.

### Histology and immunohistochemistry

Paraffin sections were stained manually with haematoxylin and eosin or on a Bond MAX immunohistochemistry robot (Leica) with antibodies at concentrations listed in Supplementary Table 4. For immunohistochemical detection, the Bond MAX DAB-Kit (Bond Polymer Refine Detection, DS9800) was used for double staining of melan A and MCAM in combination with the Bond MAX Fast Red-Kit (Bond Polymer Refine Red Detection, DS9390). Slides were scanned using a SCN400 slide scanner (Leica) and analysed using the Tissue IA image analysis software (Slidepath, Leica).

### Immunostaining of cells

Cells were detached using Versene/EDTA, incubated in DMEM for 30 min, fixed in suspension in 4% formaldehyde, washed with PBS and Tris buffer (100 mM Tris, pH = 7.4, 50 mM NaCl), permeabilised with 0.5% Triton X-100 in PBS for 10 min, incubated in blocking solution (PBS, pH = 7.4, 1% BSA, 2% FCS) for 20 min, incubated with primary antibodies at concentrations listed in Supplementary Table 4 for 12–18 h at 4 °C, washed twice with PBS, incubated with secondary antibody and phalloidin as listed under 'Antibodies and reagents' for 1 h, washed with PBS, incubated with DAPI or ToPro3 for 5–15 min and washed 4 times with PBS. Cells were mounted on glass slides with Vectashield (Vector Laboratories) and imaged on a Leica SP5 confocal microscope with a 63× oil objective using the LAS software (Leica Microsystems, Wetzlar, Germany).

### Pole orientation in vitro

SkMel2, SkMel28 or A375 cells expressing ezrin-GFP were seeded on chamber slides (µ-Slide 8-well, ibiTreat, Ibidi) uncoated or coated with 0.1% gelatine and HUVEC grown to confluence for 3 days. Cells were fixed with 4% formaldehyde 5, 30 or 45 min after seeding, washed with PBS and imaged. Stacks of 25 or 50 images of random round cells with a pole were acquired on a Leica SP5 confocal microscope with a 63× oil objective using the LAS software (Leica Microsystems, Wetzlar, Germany). Orientation of cells was assessed by two independent researchers not involved in the project. All *n* values represent biological replicates.

### Widefield live cell imaging

SkMel2 cells stably expressing ezrin-GFP were grown on chamber slides (µ-Slide 8-well, ibiTreat, Ibidi) to 70–80% confluence. Versene/EDTA was added directly at the microscope and acquisition started immediately. Fluorescence images were recorded using an Axiovert 200M and a Plan-neofluar 20×/0.5 NA objective equipped with a motorised heating stage, incubator (Zeiss Microimaging) and an AxioCam MRm camera. Using the AxioVision Software (Zeiss Microimaging), images were acquired every 30 s. Movies were exported and processed using imageJ^46^.

### Confocal live cell imaging

SkMel2 cells stably expressing ezrin-GFP were detached using Versene/EDTA, resuspended and maintained in medium for 1 h, seeded directly onto imaging slides (µ-Slide VI 0.4 ibiTreat, Ibidi) or imaging slides coated with 0.1% gelatin and HUVEC grown to confluence and imaged directly after seeding. HUVEC were stimulated with 2 ng/ml tumour necrosis factor-α (Invitrogen) for 5 h before seeding of SkMel2. Stacks of 50 confocal images over 25 µm were recorded every minute using a 63× objective (HC PL APO CS2/1.40 oil) on a Leica TCS SP8 confocal microscope equipped with 405, 488, 552 and 638 nm diode lasers and a heating chamber. Top and side views of the stacks were reconstructed using the Leica LAS software and further processed using ImageJ^46^. For temporal colour coding, the Time Series Color Coder by Kota Miura (CMCI, EMBL Heidelberg) was used.

### Interference reflection microscopy

SkMel2 cells stably expressing ezrin-GFP were detached using Versene/EDTA, resuspended and maintained in DMEM for 1 h, seeded onto imaging slides (µ-Slide 8-well, glass bottom, Ibidi) coated with 2% BSA, fixed with 4% formaldehyde and carefully washed twice with PBS. Interference reflection imaging^48^ was performed on a Leica TCS SP8 confocal microscope. Reflection and fluorescence images were obtained using 488 nm excitation and 480–497 nm or 503–559 nm emission wavelengths, respectively. To produce an IRM image, the focus was adjusted near the glass surface to obtain a zero-order interference image and the detector was adjusted for best contrast. For the reflection image, the pinhole was opened to 4 airy units (AU). In addition to the reflection image, a stack of 25 confocal fluorescence images was recorded in the GFP channel with the pinhole set to 1 AU. Top and side views of the stacks were reconstructed using the Leica LAS software and further processed using ImageJ^46^.

### Fluorescence-activated cell sorting

Cells were detached with Versene/EDTA, incubated for 30 min and fixed in 4% formaldehyde immediately or 2.5 h later. Cells were washed three times with FACS buffer (PBS containing 2% FCS and 20 mM EDTA), stained with the respective antibody as listed in Supplementary Table 4 for 30 min, washed twice with FACS buffer and, if indicated, stained with secondary Alexa-488-labelled antibody. For live/dead staining using EMA (Life Technologies), cells were not fixed but incubated with 2 µg/ml EMA for 20 min under light. Measurements were performed on a BD FACS Canto II with the FACS Diva Software. Unstained cells were used as control. A single gate (R1) was used to gate out cell debris and as stopping gate. For all measurements, this gate covered at least 90% of total events. In all, 10,000–250,000 stopping gate events were recorded for each measurement. Hardware settings were maintained constant for all experiments. Analyses were performed using the FlowJo software. Sorting of GFP-positive cells was performed on a BD FACS Aria III.

### Electron microscopy

SkMel2 cells were detached using Versene/EDTA, incubated in DMEM for 30 min and fixed in 2.5% glutaraldehyde (GA, Electron Microscopy Sciences) in 0.1 M PHEM buffer. Cells were pelleted and the subsequent EM processing steps (OSO_4_, uranyl acetate (UA), dehydration, Epon embedding) were performed on the entire pellet using a PELCO Biowave Pro microwave processor containing ColdSpot (Ted Pella, Inc.). This instrument uses microwaves under vacuum condition and controlled temperature. This has proven beneficial for EM sample preparation, as it improves diffusion of the fixatives as well as the resin during infiltration, giving better morphological results in a faster way^49^. In order to achieve this, 2 min on-and-off cycles of 100 W were applied under vacuum during glutaraldehyde and osmium steps and 1 min on-and-off cycles at 150 W for UA. For each dehydration step, samples were subjected to 40 s incubation at 250 W. Finally, infiltration was obtained with 3 min at 250 W for each step. For the entire processing, the temperature was controlled and set to 21 °C. After polymerisation of the resin at 60 °C for 48 h, blocks were sectioned with a Leica Ultracut S microtome. Seventy or 300-nm-thick sections were collected on formvar-coated slot grids and imaged with a Philips Biotwin CM120 (thin sections) or a FEI Tecnai F30 (tomography) electron microscope. For EM tomography experiments, tomogram reconstruction, segmentation and 3D rendering were carried out with the IMOD software package^50^.

### Correlative light and EM

SkMel2 cells expressing ezrin-GFP were detached using Versene/EDTA, incubated in DMEM for 30 min, pelleted, resuspended in 20% BSA (Sigma 20 µl/10 cm dish) and cryo-immobilised using a high-pressure freezing machine (HPM 010, BAL-TEC). Freeze-substitution with 0.1% UA in acetone and embedding in Lowicryl resin^51^ was performed in an AFS2 machine (Leica). The samples were incubated with 0.1% UA in acetone at −90 °C for 6 h. The temperature was then raised to −45 °C (5 degrees/h) and the samples incubated for 5 more hours. The freeze-substitution solution was then washed out with dry acetone, before infiltration in Lowicryl HM20 resin (steps: 10%—25%—50%—75%, 2 h each, followed by 3× rinses in 100% resin for 10 h each, during which the temperature was slowly raised to −25 °C). Polymerization of the resin was carried out with ultraviolet light at −25 °C. Sections (300 nm) were cut from the polymerised resin block and picked up on carbon-coated mesh grids. For fluorescence imaging, the grids were sandwiched with water between two coverlips^51^ and imaged using an Olympus ScanR microscope and a 60× oil objective. Grids were then stained with UA and Reynolds lead citrate and tilt series was acquired with a FEI Tecnai F30 electron microscope. Tomograms were reconstructed using the IMOD software package^50^. Correlation between fluorescence microscopic and EM images (single tomographic slide) was performed by alignment of the grid bars and cell and nuclear outlines visible in both imaging modalities.

### Western blotting

Cells were detached from dishes using Versene/EDTA, incubated in medium for 30 min, washed with PBS and lysed in RIPA buffer (20 mM Tris, pH = 7.4, 150 mM NaCl, 3 mM EDTA, 2% Triton-X100, 1× complete protease inhibitor (Roche), 2× phosphatase inhibitor (Roche)). Protein concentrations were determined by BCA assay (Pierce). Proteins were fractionated by sodium dodecyl sulfate-polyacrylamide gel electrophoresis and transferred onto polyvinylidene difluoride membranes. Antibodies are listed below and in Supplementary Table 4. Blots were developed on a ChemiDoc system (BioRad). Uncropped blots are shown in Supplementary Figures 10, 12 and 14.

### Antibodies and reagents

Primary antibodies, suppliers, catalogue numbers and concentrations used for immunofluorescence, FACS or western blotting are shown in Supplementary Table 4. Secondary Alexa-488 −546 or −633-labelled antibodies (Thermo Fisher Sci.) for Immunofluorescence staining were used at a 1:500 dilution. Secondary HRP-labelled anti-mouse (Jackson Immuno Research) or anti-rabbit antibodies (Cell Signaling Techn.) for western blotting were used 1:10,000. DAPI (Life Technologies) was used at 1 µg/ml, ToPro3 (Life Technologies) at 1 µM for 5–15 min, Vybrant DiI (Life Technologies) at 5 µl/ml for 10 min and phalloidin-Alexa-546 or -Alexa-633 (Life Technologies) at 1:500 for 30 min. CalyculinA (Cell Signaling Techn.) was used at 50 nM or 10 nM as indicated for 1 h. (−) Blebbistatin (Merck Millipore) was used at 2.5 µM for 24 h.

### Plasmids, siRNAs and shRNAs

pEGFP-ezrin and ezrin mutants were kindly provided by Professor Richard Lamb (University of Liverpool), mCherry-ezrin and CFP-ezrin by Professor Christopher Marshall (ICR, London), GFP-MLC and GFP-dnMLC by Professor Michael Olson (Beatson Institute, Glasgow), plasmids expressing RFP-labelled PH domains by Professor Tamas Balla (NICHD, NIH, Bethesda), PKCζ-GFP by Professor Peter Parker (CRICK Institute, London), pEGFP-MCAM by Professor Natalie Ahn (Unversity of Colorado Boulder), pcDNA3-mouseMCAM by Professor Judith Johnson (University of Munich), pEPIto-deltaSMAR by Professor Manfred Ogris (University of Vienna), ICAM1-GFP by Professor Francis Luscinskas (Harvard Medical School, Boston), L1-CAM-Venus by Professor Dan Felsenfeldt (Mount Sinai School of Medicine, NY) and β1-Integrin-GFP by Professor Maddy Parsons (King’s College London). To generate pEpi-MCAM, mouse *Mcam* was amplified by PCR and inserted into the *Bgl*II/*Nhe*I sites in pEPIto-deltaSMAR (replacing GFP). To generate mCherry-MCAM, GFP in pEGFP-MCAM was replaced by mCherry using the *Bam*HI/*Not*I sites. MCAMΔKKGK constructs were created by one-step mutagenesis PCR^52^ to delete the KKGK motif in pEpi-MCAM, pmCherry-MCAM and pEGFP-MCAM using the primers ctatttcttctacctgccatgtg-gacgctcgggaaaacag and catggcaggtagaagaaatagagagcagcacccagcacag for mouse and ttcctctatctgccgtgcaggcgctcagggaagcagg and cacggcagatagaggaaatagaggacagcgcccag for human *MCAM*. SiRNA pools against human *MCAM*,* NF2*, *ezrin* and non-targeting control pool were on-target plus smart pools from GE Healthcare. Lentiviral particles containing shRNAs against mouse Nf2 (clones TRCN0000042519 and TRCN0000042520) and mouse *Mcam* (clones TRCN0000113080 and TRCN0000113081) were from Sigma (MISSION lentiviral transduction particles, SHCLNV and non-targeting control SHC002V).

### Animals

Mice were maintained under standard housing conditions and experiments were performed according to the guidelines of the Swiss Animal Protection Law and approved by the Veterinary Office of Kanton Zurich (TVA 20/2011) or according to the German Animal Protection Law and approved by the Regierungspräsidium Karlsruhe (TVA g240/11) or the Regierung Oberbayern, Bavaria, Germany (TVA 55.2-1-54-2532-196-13).

### Experimental metastasis

Female C57BL/6 mice of 8–10 weeks (Charles River Laboratories, Germany) were intravenously injected with 1.5 × 10^5^ B16-F1, B16-F1-Nf2kd (M19), B16-F1 Nf2kd (M20), B16-F1 Mcamkd (M1), B16-F1 Mcamkd (M2), B16-F1 n.t. (expressing non-targeting shRNA control), B16-F0, B16-F0 GFP, B16-F0 Mcam or B16-F0 McamΔKKGK cells in random order. Mice were sacrificed after 2 weeks. Lungs were perfused with PBS, fixed in 4% formaldehyde for 3 days, photographed and the number of metastatic foci determined blinded by visual inspection. Numbers were normalised to the mean of the untransfected control for each experiment. Lungs were embedded in paraffin blocks and 2 µm sections stained for haematoxylin/eosin or ki67. No statistical methods were used to predetermine sample sizes. Sample sizes were based on previous experience with the models. All *n* values represent biological replicates.

### Metastatic seeding to the mouse lung

Female C57BL/6 mice of 8–10 weeks (Charles River Laboratories, Germany) were intravenously injected with 5 × 10^5^ SkMel2 (ezrin-GFP), U87 (GFP) or SNU-1 (GFP) cells either polarised (30 min) or depolarised (3 h after detachment) or with 1.5 × 10^6^ B16-F1, B16-F1 Mcam kd (M1), B16-F1 Mcam kd (M2), B16-F1 n.t. (expressing non-targeting shRNA control), B16-F0, B16-F0 GFP, B16-F0 Mcam or B16-F0 McamΔKKGK cells in random order. Thirty minutes after injection, mice were sacrificed, the lungs perfused, fixed in 4% formaldehyde for 3–5 days and embedded in paraffin in 2–4 pieces. Two-µm tissue sections were stained with haematoxylin/eosin and with GFP, ki67 or gp100 antibody. All *n* values represent biological replicates.

### Quantification of metastatic seeding to the mouse lung

Slides were scanned using a Leica SCN400 slide scanner at 20× magnification. Analysis was performed using Tissue IA (Slidepath, Leica). All slides within an experiment were analysed with the same algorithm and settings. For each slide, 2–3 annotations were made to cover most of the tissue avoiding staining artefacts and merged in the results.

For generic depolarisation experiments, 2 step cuts of 100 µm, and for MCAM overexpression and kd experiments, 2–3 lung sections of each lung were analysed using an algorithm for stained area with optimised colour definition files with deconvoluted intensity threshold of 161 (generic depolarisation) or 165 (MCAM manipulation). Tissue intensity threshold was set to 211 or 215, respectively. For generic depolarisation experiments, the haematoxylin-positive area was measured using a predefined deconvolution-haematoxylin colour definition file with a deconvoluted intensity threshold of 230. All slides were analysed as GFP+ area per total tissue area, GFP+ area per haematoxylin+ area and GFP+ cells per total amount of cells. SkMel2 were in addition analysed by manual counting of GFP+ cells in 10 randomly chosen 40× view fields per slide. For MCAM overexpression or kd experiments, all slides were analysed as melanoma gp100+ area per total tissue area.

### 3D reconstruction of GFP^+^ cells seeding in the lung

3D reconstruction of an anatomical structure from 30 serial sections was generated using the Voloom software (microDimensions GmbH, München, Germany). The virtual slide data was loaded into Voloom where individual sections were automatically detected and separated. For 3D reconstruction, consecutive sections were registered to each other avoiding error propagation due to micro-artefacts like folding or tears. An initial reconstruction result on low-resolution images was propagated from low- to high-magnification levels inherently using the pyramidal structure of virtual slides. The anatomical structure was then segmented from the volumetric histology data using global thresholding and rendered with a green pseudo colour. The rendering process is shown in Supplementary Movies 17 and 18.

### Generation and analysis of CTCs from MDA-MB-231 xenografts

Six-week-old NSG immunocompromised mice (Jackson Laboratories) underwent surgery under isofluorane anestesia. A cell suspension containing 100,000 GFP-positive MDA-MB-231 cells in 50 µl PBS was mixed with one volume of growth factor-reduced Matrigel (Corning) and injected into the fourth right mammary fat pad. Mice were housed under specific pathogen-free conditions in individually ventilated cages and maintained according to the German federal law, authorization number G-240/11. Mice were monitored weekly for tumour growth by palpation, and after 5 weeks, tumours were surgically resected from mice upon anesthesia by intraperitoneal injection of 10 ml/g of a solution of 4.5 mg/kg xylazinhydrochloride and 90 mg/kg ketamine. Surgically resected mice were closely monitored until clear signs of pain appeared; hence, they were then euthanized by anesthetic overdose according to local regulations. The peripheral blood was collected by heart puncture in EDTA tubes and mononuclear cells were separated by Ficoll-Paque gradient. The lungs were washed with PBS and digested enzymatically, using type IV collagenase and dispase, and mechanically using a *Gentle*MACS device (Miltenyi). Circulating or disseminated cells were FACS-purified from these preparations as live intact GFP-positive cells that were negative for a mouse lineage antibody cocktail (including antibodies specific for mCD45, mCD31, mCD11b, Ly-6G and TER-119) using a FACSAria Fusion (BD) cytometer. Cells were immediately fixed in a 1.6% paraformaldehyde solution upon sorting.

### Patient samples

All study samples were obtained by informed consent. Human liver samples from patients were obtained from the Department of General Surgery, Klinikum rechts der Isar, Technical University Munich; experiments were authorised by the ethics committee at the Technical University Munich (ethics vote number 156/15). Analyses of human CTCs from blood and human lymph nodes with disseminated tumour cells were carried out in accordance with the ethical committee in Regensburg, Germany (ethics vote number 07/79). CTC analysis of breast cancer patients was performed within DETECT studies (EUDRA-CT number 2010-024238-46)^53^. PEs and ascites fluids from primary punctates from metastatic breast cancer patients for isolation of AE and PE cells were obtained from the University Clinic Mannheim, Department of Gynecology (Frauenklinik) and experiments were approved by the ethics committee of the University of Heidelberg-Mannheim (case number 2011-380N-MA). PEs from a pancreatic carcinoma patient for isolation of PE cells were obtained from the Department of Internal Medicine II at Klinikum rechts der Isar, Technical University Munich. Experiments were authorised by the ethics committee at the Technical University Munich (ethics vote number 5542/12). Analyses of human melanoma tissue were carried out in accordance with the ethical committee in canton Zurich (StV 16-2007; amendment 2014).

### Ex vivo human liver perfusion

Fresh surgical human liver biopsy (26 g) was perfused via portal vein branches through two G20 cannules in closed circuit at 37 °C/5% CO_2_ with perfusion medium (50 ml Leibovitz’s L15 medium without phenol red (Life Technologies) supplemented with 5% FCS, 1 µg/ml insulin (Serva), 23 mM HEPES pH 7.4, 5 mM L-glutamine, 50 IU/ml penicillin and 50 µg/ml streptomycin (Gibco), 100 µg/ml Gentamycin and 4.4 µg/ml Hydrocortisol at a flow rate of 1 ml/min/g. In all, 10^6^ SkMel2 cells stably expressing ezrin-GFP were injected via the perfusion cannules at 1 ml/min/g and the liver perfused with medium for 5 further minutes. The liver was then fixed in 4% formaldehyde for 3 days and embedded in paraffin. Liver integrity was confirmed by haematoxylin/eosin staining, and sections were stained for GFP and vimentin and imaged on a Leica SP5 confocal microscope with a 63× oil objective using the LAS software (Leica Microsystems, Wetzlar, Germany). To quantify the orientation of cell polarisation, random round cells in vessels were imaged, where not more than half of the circumference of the cell was attached to vimentin-positive, GFP-negative cells. The orientation of cells was assessed by two independent researchers not involved in this project. Only cells with consistent orientation in both assessments were included in the quantification (40 out of the 58).

### Human CTCs

CTCs of breast cancer patients were enriched and detected using the CellSearch® Epithelial Cell Test (Janssen Diagnostics, LLC). In brief, 7.5 ml of blood was collected in CellSave tubes and CTCs captured by anti-epithelial cell adhesion molecule (anti-EpCAM) antibody-bearing ferrofluid. CTCs were defined by positivity for cytokeratin, negativity for the leukocyte common antigen CD45 and DAPI positivity to ensure integrity of the nucleus. After quantification, stained cells were flushed from the cartridge and one aliquot was used to isolate single CTCs and white blood cells by DEPArray^TM^ (Menarini Silicon Biosystems) for subsequent whole-genome amplification by Ampli1^TM^ according to the manufacturer’s protocol (Menarini Silicon Biosystems)^54^. The remaining cells were stored in glycerol^55^, sedimented on adhesion slides (Thermo Fisher Scientific), stained for ezrin/Alexa 488 as described above and imaged on a Leica TCS SP8 confocal microscope.

### Single-cell array comparative hybridisation

Ampli1TM whole-genome amplification libraries were checked for genome integrity index and then reamplified^54^. Labelling of CTC DNA and corresponding reference DNA from WGA libraries of single leukocytes of a healthy female donor and hybridisation on SurePrint G3 Human CGH 4×180K microarray slides (Agilent Technologies, design code 022060) were performed according to the manufacturer’s protocol (Agilent Oligonucleotide Array-Based CGH for Genomic DNA Analysis, version 7.1, December 2011)^56^. Slides were scanned using an Agilent Microarray Scanner Type C, and images were processed with Agilent Genomic Feature Extraction Software (version 10.7) and imported and analysed with the Agilent Genomic Workbench Software (version 6.5 lite). For defining aberrant regions, we used the ADM-2 algorithm with threshold set to 7.0 and a centralisation of 6.0. To avoid false positive calls, the minimal number of probes in an aberrant interval was set to 50 probes and minimum log2 ratio to 0.25.

### Tumour cells from PEs and AEs

Fresh pleural or peritoneal punctates were processed immediately. The whole cellular fraction was frozen and stored or immediately depleted from most CD45+ cells using magnetic human CD45 microbeads (Miltenyi Biotec 130-045-801). Cells were stained with EpCAM-FITC (Miltenyi Biotec) and CD45-APC (Miltenyi Biotec) in suspension or further purified by sorting of singlet-gated EpCAM+CD45−DAPI− cells on a FACSAria^TM^ Fusion device (BD Biosciences) and fixed in 1.6% PFA. Cells were stained as indicated, attached to microscopy slides and imaged on a Leica TCS SP8 confocal microscope.

### Human disseminated cancer cells

Adhesion slides with preparations of human lymph nodes from five melanoma patients and four non-SCLC patients were prepared^57^. Lymph nodes were mechanically disintegrated using a Medimachine (Beckham Coulter) and cells were subsequently enriched using percoll 60% gradient centrifugation. A total of 2 × 10^6^ cells (at a density of 1 × 10^6^ cells/ml PBS) were transferred onto adhesion slides (Thermo Fisher Scientific). After 1 h sedimentation, PBS was removed. Slides were air-dried overnight at room temperature and fixed for 10 min with 4% formaldehyde prior to permeabilisation and staining as described above. Samples were stained for gp100 or EpCAM, ezrin and DAPI and imaged on a Leica TCS SP8 confocal microscope. In two out of the three melanoma samples and in two out of the two lung cancer samples that showed sufficiently high ezrin expression, polarisation of ezrin was observed. Control (non-cancer) nodes were obtained from patients with chronic venous insufficiency from whom a lymph node was removed during crossectomy. For each experiment, three negative controls were stained and analysed.

### Human melanoma specimens

TMAs^21^ comprised 70 primary melanomas, 216 melanoma metastases and melanoma patient’s cell lines consisting of 41 cell lines derived from primary and metastatic melanomas. Primary tumours and metastases were mostly not related. TMA specification and construction were previously performed^21^. In all, 2 µm sections from these TMAs were immunohistochemically stained for melan-A and MCAM or fluorescently for melan-A and ezrin. Ezrin polarisation was imaged on a Leica SP5 confocal microscope with a 63× oil objective.

### Evaluation of MCAM expression

To determine the expression frequencies of MCAM, a semi-quantitative scoring system was applied following the German immunohistochemical scoring (GIS) system in which the final immuno-reactive score equaled the product of the percentage of positive cells times the average staining intensity. Percentage of positive cells was graded as follows: 0 = negative, 1 = up to 10% positive cells, 2 = 11–50%, 3 = 51–90%, 4 = > 90%. Staining intensity of 0 = negative, 1 = weakly positive and 2 = moderately or strongly positive^58^. GIS score ≥4 was defined as high and <4 as low. All stainings were evaluated by an experienced pathologist (D. M.-P.). MCAM expression was compared between different patient groups using Pearson’s Chi-squared Test. *p*-values <0.05 were considered statistically significant.

### Statistical analyses

Descriptive statistics of quantitative data are mean ± standard deviation or median and other quantiles, depending on the distribution of data. Likewise, qualitative data are presented by absolute and relative frequencies. Hypothesis testing was performed in an exploratory manner on two-sided 5% significance levels. Group differences of normally distributed data were assessed by *t*-tests. One-sample *t*-tests and paired-samples *t*-tests were used for dependent data. Relations of the latter were assessed by the Pearson correlation coefficient. Mann–Whitney *U*-tests were used to infer on group differences of non-normally distributed data. Differences in the distribution of qualitative data were investigated by Pearson’s Chi-squared Test. Error bars are mean ± standard deviation. SPSS 22 software (SPSS Inc.) and GraphPad Prism were used for statistical analysis.

### Analysis of pole morphology

Sixteen-bit images of SkMel2 cells expressing ezrin-GFP were converted to 32-bit without interpolation and the background was subtracted. Yen thresholding was used to segment cellular regions of low intensity and ezrin clusters of high intensity. Object-based regions were defined and utilised to measure the area and intensity of the identified cells and ezrin clusters.

'Aggregation score' for ezrin was defined as:

$${\mathrm{Aggregation}}{\mathrm{ = }}\left[ {\frac{{I_{{\mathrm{cluster}}}}}{{I_{{\mathrm{cell}}}}} \times \frac{{A_{{\mathrm{cell}}}}}{{A_{{\mathrm{cluster}}}}}} \right]$$

where *I* is the intensity and *A* is the area of large ezrin clusters per cell. This agregation score is maximised when ezrin is strongly confined to a single cluster in a cell (spot-like) and minimised when it is dispersed over the cell (cap-like). A histogram of the aggregation scores was generated and fitted to a double modified-Gaussian peak model (Supplementary Table 5) to disentangle the two populations of cells. Image processing and calculation of aggregation score were performed using a single automated Python script provided as ‘EzrinClassifier.py’. Dependencies: tifffile, NumPy, SciPy, pandas, Pillow, and scikits-image.

### In silico modelling

Model set-up: An ODE-based compartmental model was constructed using the Simbiology toolbox in MATLAB R2016a. Three independent compartments corresponding to blood circulation, attachment/adherence substrate for cells and tissue parenchyma were defined in the model (Fig. 10a). Each compartment contains populations of polarised and depolarised cells. Transfer of cells between compartments was defined as occurring according to first-order reaction kinetics as follows:

1 $$\frac{{{\rm d}({\rm Attached}_{{\rm pol}})}}{{{\rm d}t}} = k_{{\rm pol}}^{{\rm attachment}} \cdot {\rm Circulating}_{{\rm pol}}$$

2 $$\frac{{{\rm d}({\rm Attached}_{{\rm depol}})}}{{{\rm d}t}} = k_{{\rm depol}}^{{\rm attachment}} \cdot {\rm Circulating}_{{\rm depol}}$$

3 $$\frac{{{\rm d}({\rm Adhered}_{{\rm pol}})}}{{{\rm d}t}} = k_{{\rm pol}}^{{\rm adherence}} \cdot {\rm Attached}_{{\rm pol}}$$

4 $$\frac{{{\rm d}({\rm Adhered}_{{\rm depol}})}}{{{\rm d}t}} = k_{{\rm depol}}^{{\rm adherence}} \cdot {\rm Attached}_{{\rm depol}}$$

5 $$\frac{{{\rm d}({\rm Tissue})}}{{{\rm d}t}} = k_{{\rm depol}}^{{\rm tissueresidence}} \cdot {\rm Adhered}_{{\rm depol}} + k_{{\rm pol}}^{{\rm tissueresidence}} \cdot {\rm Adhered}_{{\rm pol}}$$

The first-order reaction rates for each transfer were determined by linked-fitting the appropriate steps in the model to three independent sets of experiments,

i. in vitro experiments of attached polarised or depolarised cells to measure the adherence rate (Fig. 9d), fitted to Eqs. 3 and 4
ii. in vitro experiments of mixed populations of polarised and depolarised cells in suspension to measure their combined ‘attachment and adherence’ rates (Fig. 9e) and fitted to Eqs. 1–4
iii. in vivo experiments of a mixed population of polarised and depolarised cells injected into blood circulation to measure their combined ‘attachment, adherence and tissue residence’ (Fig. 9k and Supplementary Fig. 18a), fitted to Eqs. 1–5

These experiments allowed us to fit all the required rate parameters and obtain predictions for the attachment and tissue residence rates of polarised and depolarised cells individually.

For each compartment, the polarised and depolarised cells are represented as distinct species. The only exception is for the tissue compartment since the experimental data do not provide information regarding the previous polarisation status of cells that are already tissue resident.

Fitting procedures: The data from all three independent experiments described above was pooled into a single matrix that contained the observed circulating, adhered, attached and tissue-resident cell populations for polarised and depolarised cells. The model fitting procedure did not contain any information regarding the biological/technical nature of the experiment, effectively making the result a linked fit of all three experiments. Linked fitting constrains the parameter space to only those ranges which fit data from all three experiments simultaneously. Fitting was performed using the ‘constrained mixed effects non-linear least squares optimization’ (nlmm-lsqnonlin) algorithm^59^, which is particularly suited for data from mixed populations, coming from disparate experiments. Termination tolerances were set as ResidualSumofSquares = 1.0E-8 or MaxIterations = 400. The linked-fit-derived rates were then tested against the three experiments individually to see if they predicted the observations to within 95% confidence bounds.

Simulations: The experimental data providing the fitted first-order rate parameters typically consist of mixed but biased populations of polarised and depolarised cells. Once obtained, the rate parameters can be used to simulate the behaviour of ‘pure’ populations of polarised and depolarised cells in terms of tissue residence. Simulations were then performed using the derived rate parameters for an interval of 15,000 s (250 min), setting the populations of polarised or depolarised cells to 100% and then computing the tissue residence.

The model is provided as an SBML file, datasets are provided as annotated CSV files and the results of simulations as an OPJ file (the viewer can be obtained under //www.originlab.com/viewer/). All data are also provided as one single SBPROJ file that is readable in MATLAB with Simbiology toolbox.

Results and predictions: The compartmental model fitted the data to within 95% confidence limits for all parameters (Supplementary Fig. 19a). The obtained transfer rates are provided in Supplementary Figure 19b. In agreement with experimental indications (Fig. 5), the model predicts a three-fold higher attachment rate of polarised cells compared to depolarised cells. Similarly, in agreement with experiments (Fig. 9d), the adherence rate of polarised cells is approximately 1.3-fold the adherence rate of depolarised cells. Both cell populations are predicted to have much slower tissue invasion rates. The tissue invasion rate of polarised cells is only slightly enhanced compared to depolarised cells. These rates were used to simulate the entire chain of events from circulation to tissue residence for pure unmixed populations of polarised and depolarised cells (Fig. 10b–e). Such unmixed populations cannot be obtained experimentally, justifying the development of the aforementioned compartmental model. The changes in the circulating, attached, adhered and tissue-resident populations are shown in Supplementary Fig. 19c. Direct comparisons for all rates between polarised and depolarised cells are shown in Fig. 10b–e.

### Code availability

Custom code used for analysis and simulations are available as Supplementary Information.

### Data availability

The authors declare that the data supporting the findings of this study are available within the paper and its Supplementary Information files or available from the authors upon request.

## Electronic supplementary material

Supplementary Information

Peer Review File

Description of Additional Supplementary Files

Supplementary Movie 1

Supplementary Movie 2

Supplementary Movie 3

Supplementary Movie 4

Supplementary Movie 5

Supplementary Movie 6

Supplementary Movie 7

Supplementary Movie 8

Supplementary Movie 9

Supplementary Movie 10

Supplementary Movie 11

Supplementary Movie 12

Supplementary Movie 13

Supplementary Movie 14

Supplementary Movie 15

Supplementary Movie 16

Supplementary Movie 17

Supplementary Movie 18

Supplementary Software

Supplementary Data 1

Supplementary Data 2

Supplementary Data 3

Supplementary Data 4

Supplementary Data 5
