# Supplementary material for: Single cell polarity in liquid phase facilitates tumour metastasis
Source: Nat Commun. 2018 Feb 28;9:887. doi: 10.1038/s41467-018-03139-6 (PMC5830403; doi:10.1038/s41467-018-03139-6)
Supplement: Supplementary file 1 — Supplementary Information [file 41467_2018_3139_MOESM1_ESM.pdf]

Lorentzen *et. al.*

Single cell polarity in liquid phase facilitates tumour metastasis

Supplementary Information

Supplementary Figure 1

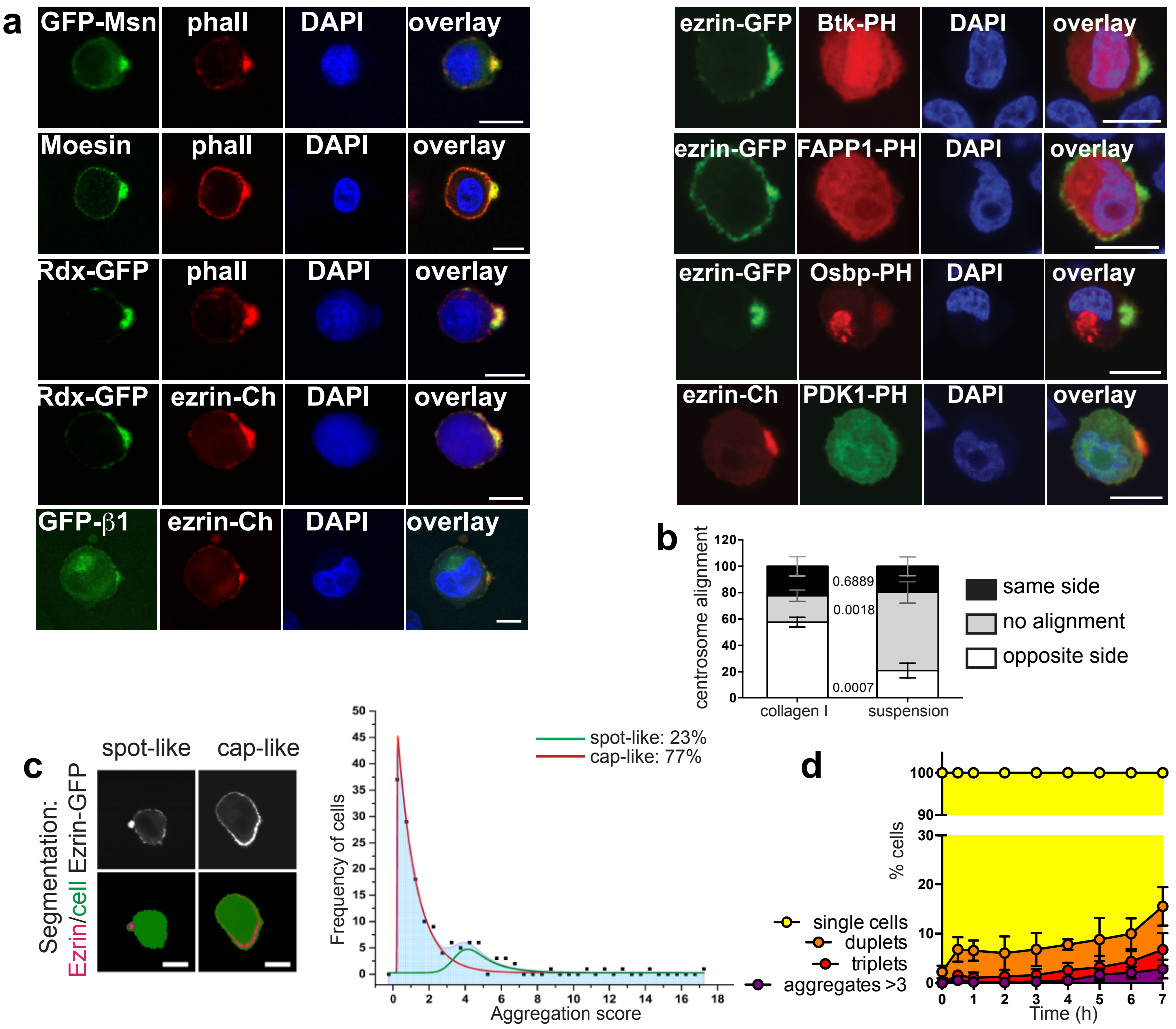

Characterisation of sc polarity.
(a) Supplementary Data to Fig.1.Confocal images of SkMel2 cells in suspension expressing the indicated proteins (Msn: moesin, Rdx: Radixin, β1: β1-Integrin, Ch: mCherry; PH: RFP-labelled PH domains of Btk1, FAPP1, Osbp or PDK1; PKCζ-GFP: GFP-labelled protein kinase C ζ) or stained for moesin or actin (phall) and DAPI. Scale bars: 10µm.
(b) Alignment of centrosome, nucleus and sc pole in GFP-expressing SkMel2 cells during amoeboid migration on top of collagen I or 15 minutes in suspension. Centrosome positioning at the same side of the nucleus as the sc pole (black), not aligned with nucleus and pole (grey) or located at the opposite side of the nucleus as compared to the sc pole (white) was assessed by pericentrin staining. 25-29 single cells were analysed per measurement (n=3, mean ± SD, unpaired t-tests).
(c) Representative cells showing the two localisation patterns of Ezrin-GFP, aggregated spot-like and less aggregated (but still asymmetrical) cap-like and automated segmentation (below). Right: Histogram of SkMel2 cellular ezrin aggregation score in 143 cells from 3 independent experiments after 1 hour in suspension based on intensity and spread of ezrin clustering, showing two populations corresponding to spot-like (23%) and cap-like (78%) poles. Scale bars: 10 µm.
(d) Quantification of the number of singe cells, cell duplets, cell triplets and aggregates (>3 cells) of SkMel2 cells detached from substrate and maintained in suspension for the indicated time. Between 110 and 450 cells were analysed for each measurement and each time point (n=6, mean ± SD). Cells start to form triplets and aggregates after 4 hours.

Supplementary Figure 2

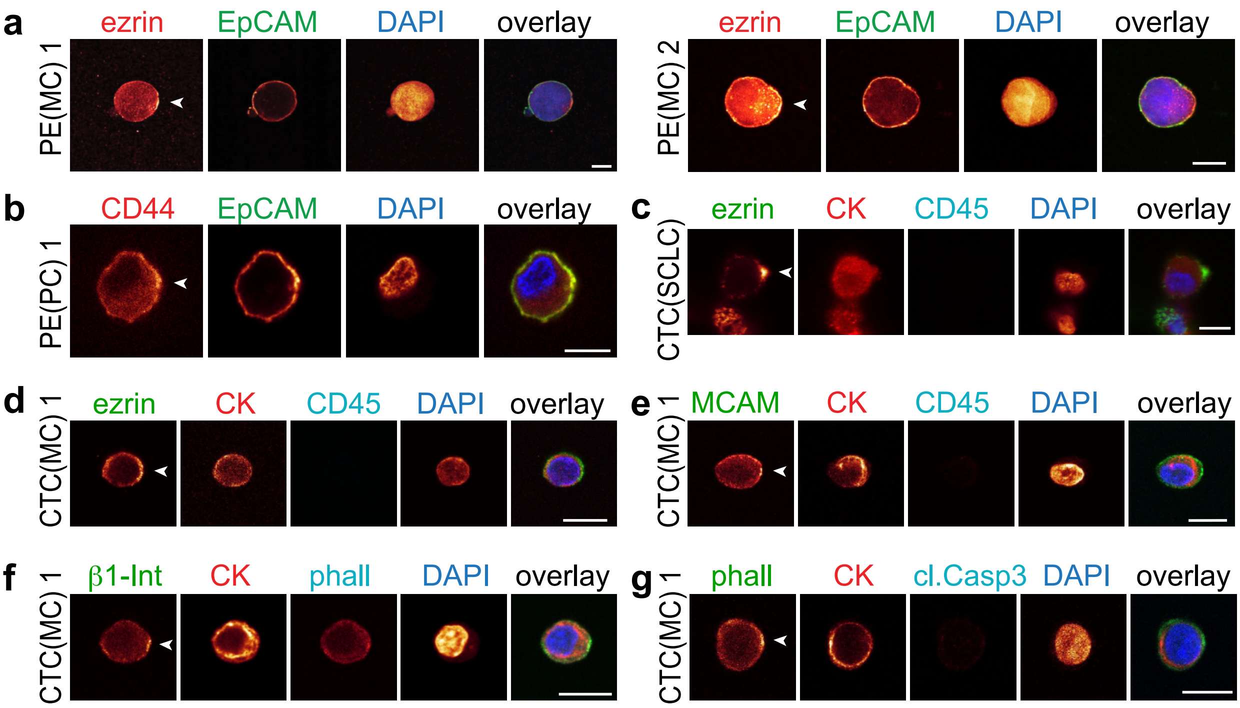

Polarised PE cells and CTCs.
(a) Polarised ezrin localisation in PE cells from two breast cancer patients.
(b) Polarised CD44 localisation in a PE cell from patient sample PE(PC) 1 shown in Fig. 3b.
(c) Polarised ezrin localisation in a CTC from a cluster of 4 CTCs from an SCLC patient.
(d-f) Further examples of polarised CTCs from patient sample CTC(MC) 1 shown in Fig. 3c stained for cytokeratin (CK), CD45, DAPI and ezrin **(c)**, MCAM **(d)** β1-Integrin (β1-Int, **e)** or actin by phalloidin (phall, **f).**

Supplementary Figure 3

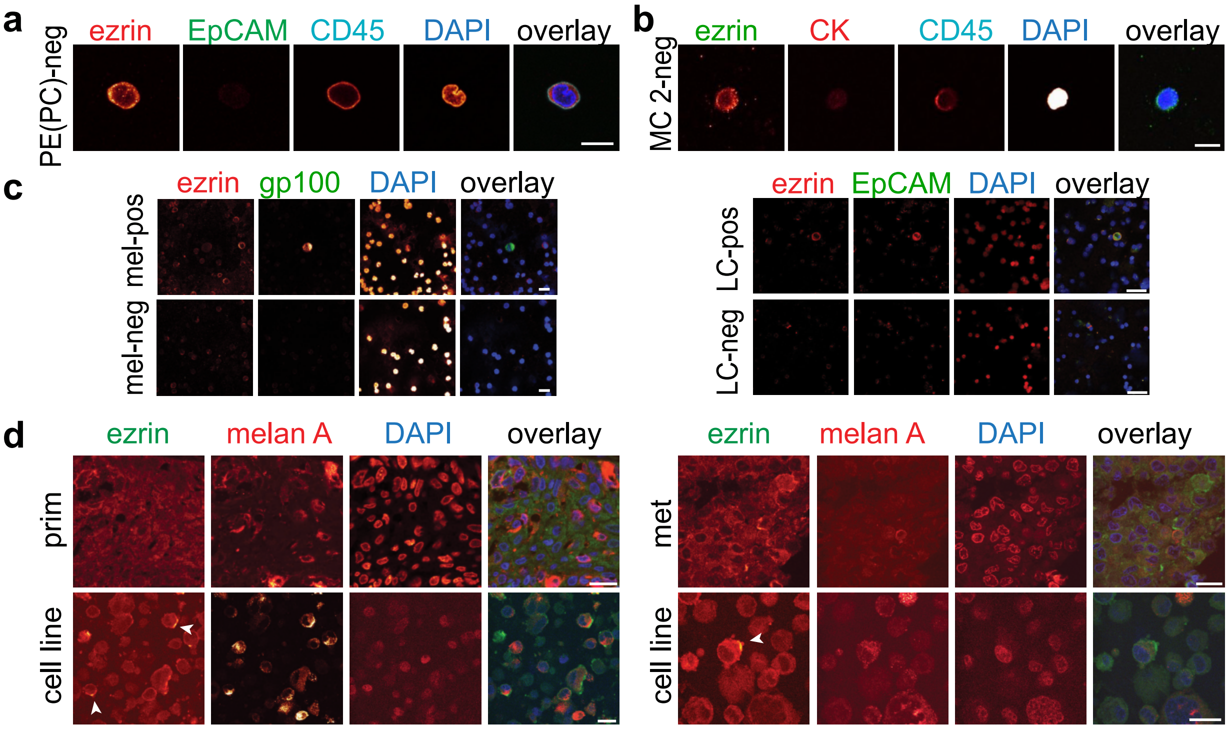

Sc polarity in patient samples.
(a) Negative (EpCAM-CD45+) cell in the preparation of PE(PC) 1 shown in Fig. 3b. Scale bar: 10µm.
(b) Negative (CK-CD45+) cell in the preparation of circulating breast cancer cells (CTC(MC) 2) shown in Fig. 3c. Scale bar: 10µm.
(c) Controls to Fig. 3d. Disseminated tumour cells from lymph nodes of melanoma (left) or lung cancer (ight) patients, stained for gp100 or EpCAM, ezrin, and DAPI. Scale bars: 20µm.
(d) Paraffin samples of human primary (left) or metastatic (right) melanoma (top panel) and the respective cell lines (bottom panel) derived from these tumours in suspension stained for ezrin, melan A and DAPI. Scale bars: 10µm. Arrowheads indicate sc poles.

Supplementary Figure 4

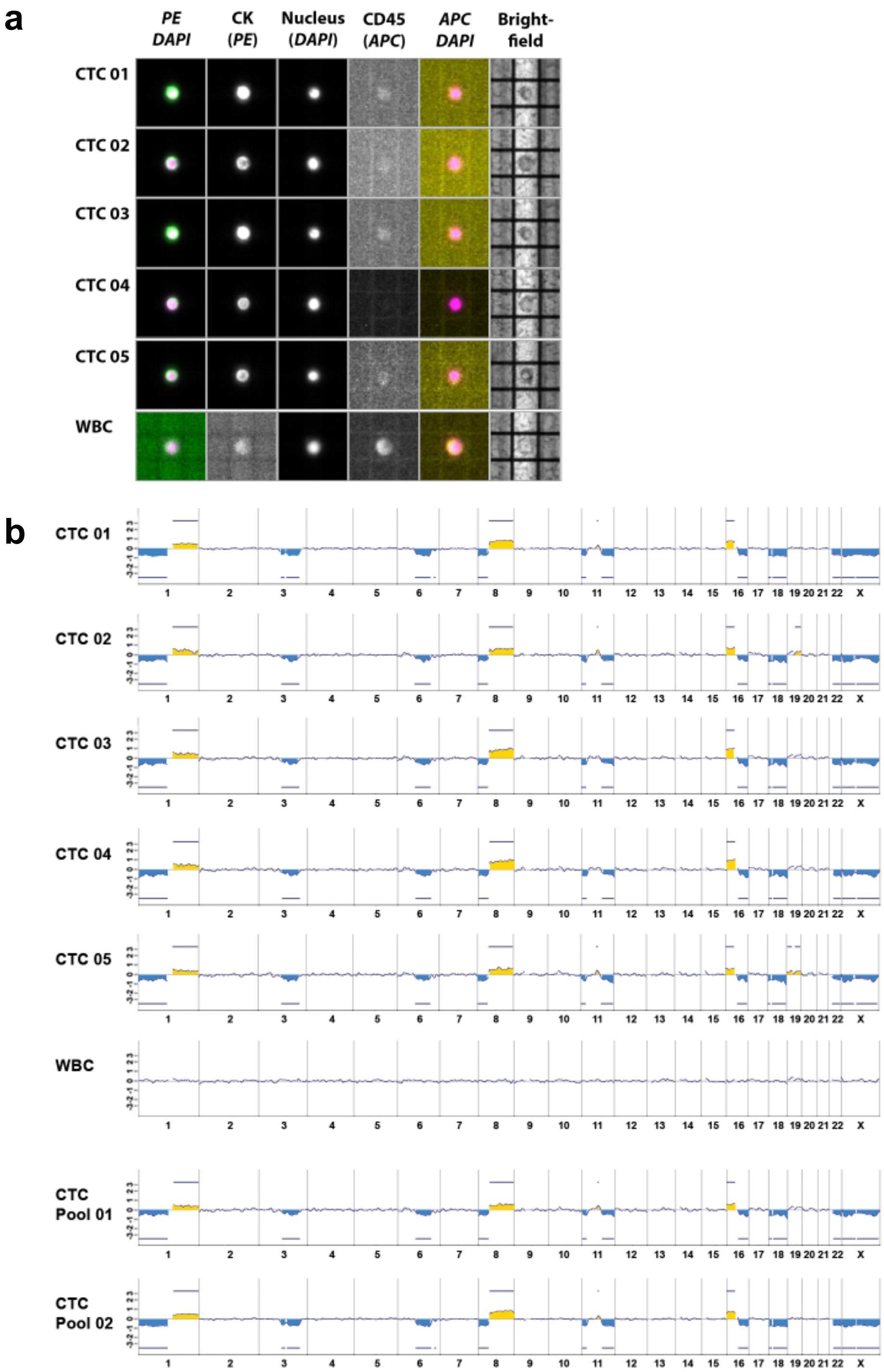

Isolation and aCGH of human CTCs.
(a) Images of CTCs (MC) 1 isolated using the CellSearch system shown in Fig. 3c. WBC: white blood cell.
(b) aCGH profiles of single CTCs (01-05) and CTC pools from sample (MC) 1 isolated using the CellSearch system shown in Fig. 3c. WBC: white blood cell.

Supplementary Figure 5

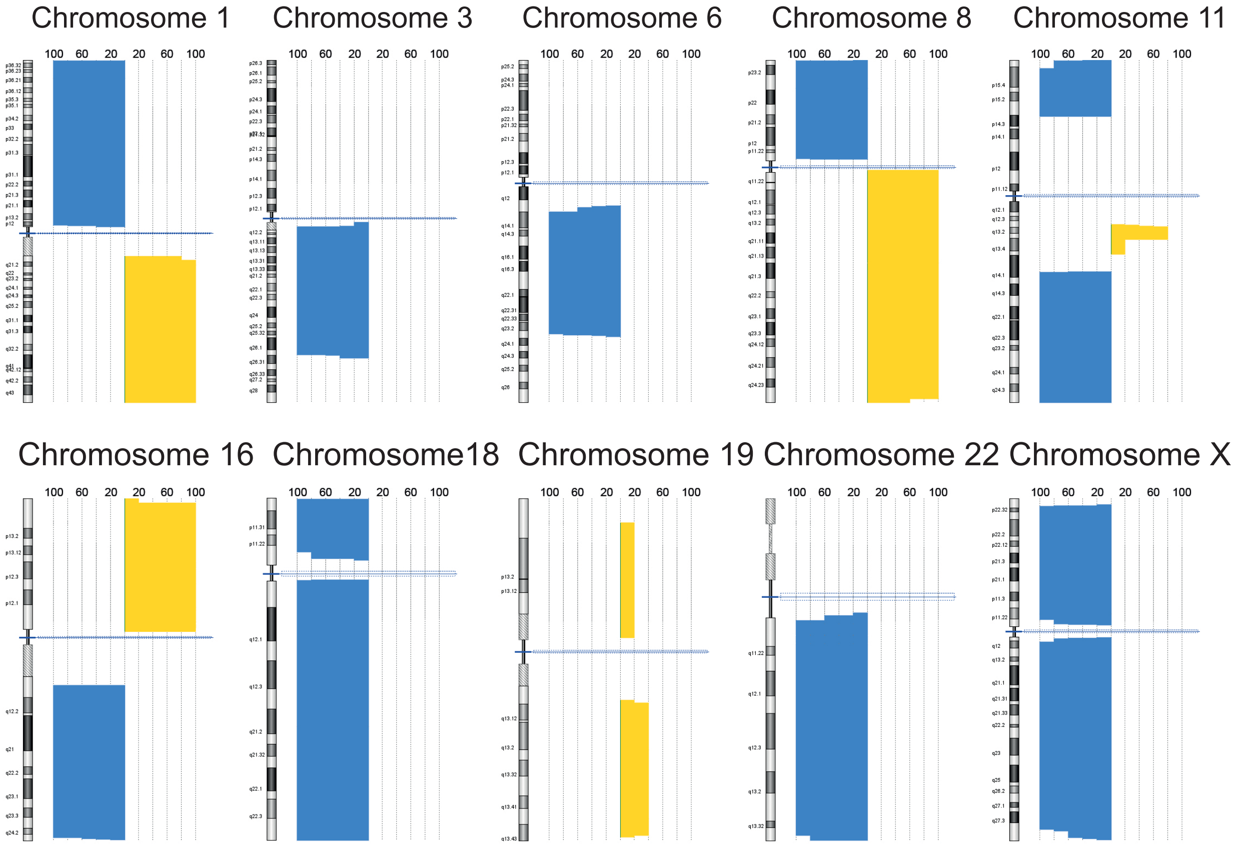

Identification of human CTCs.
Cumulative histograms of genomic aberrations for selected chromosomnes as determined by single cell array comparative hybridisation (aCGH) of five single CK+CD45-DAPI+ cells isolated from the sample CTC(MC) 1 shown in Fig. 3c.

Supplementary Figure 6

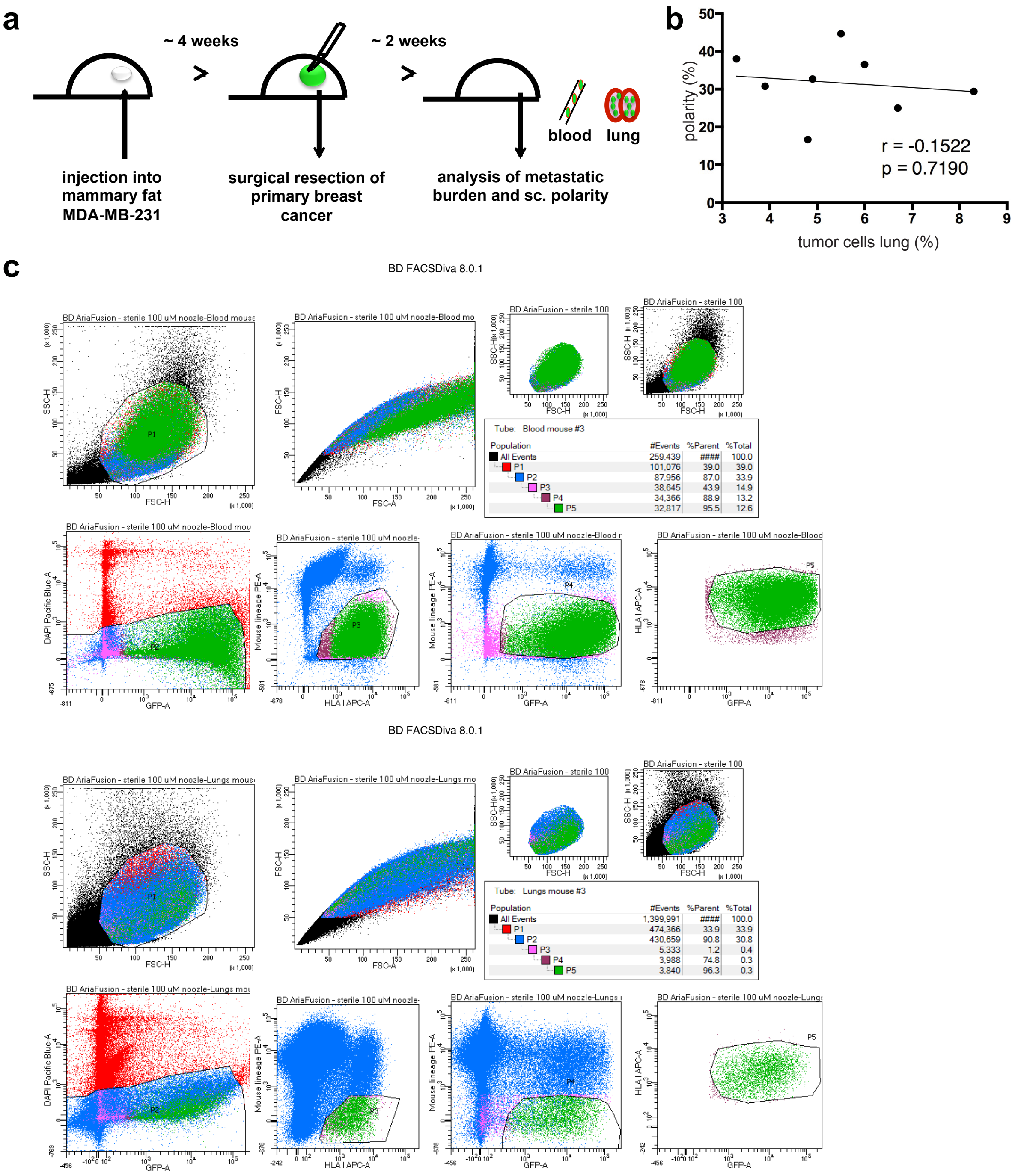

Orthotopic mouse model for CTCs.
(a) Sketch of the experimental setup for the experiment shown in Fig. 4a.
(b) Tumour cells in the lung plotted against sc polarity from the mouse experiment shown in Fig. 4a
(c) Gating for blood (top) and lung(bottom) preparations of one representative mouse.

Supplementary Figure 7

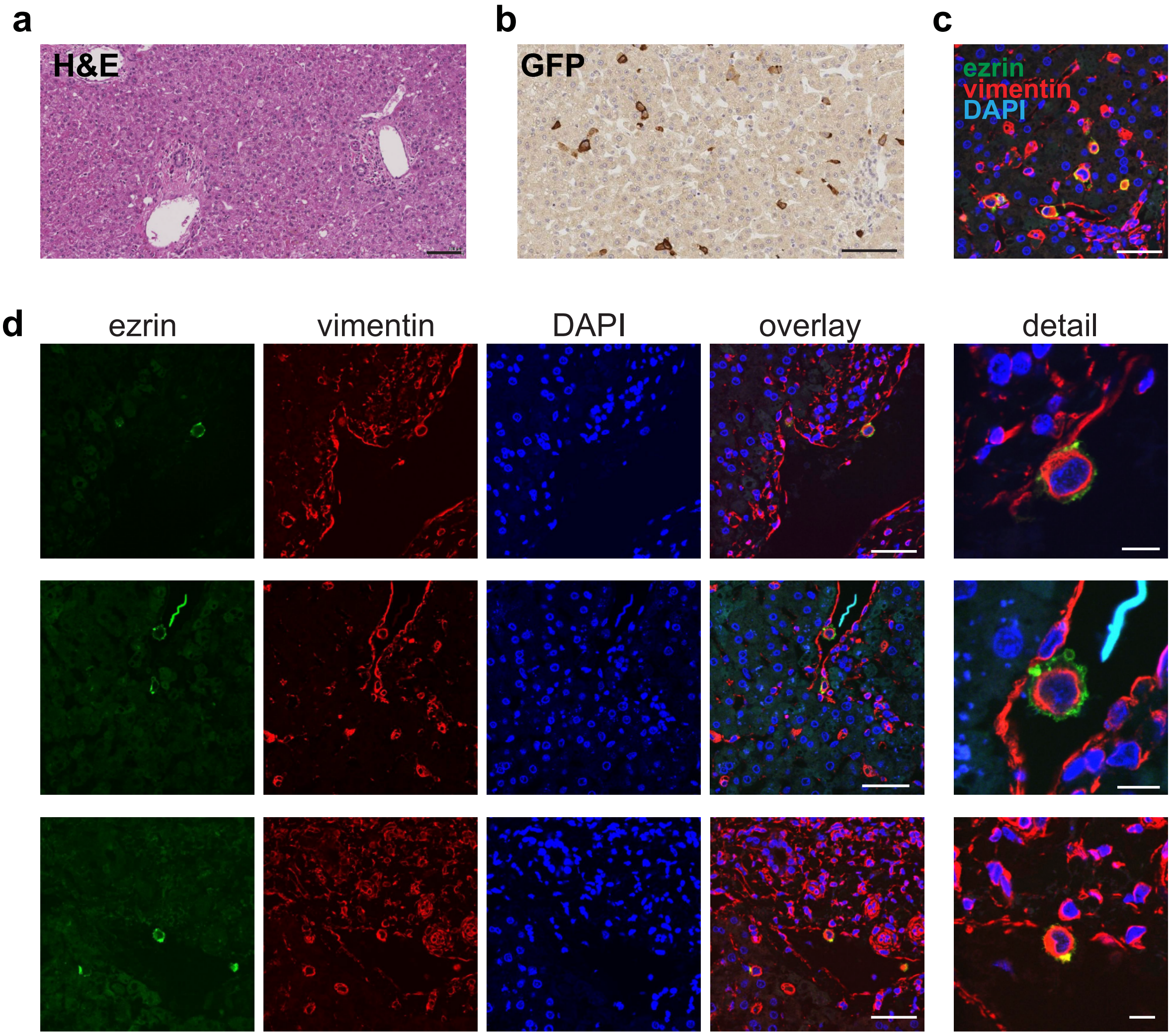

Seeding of tumour cells in human liver *ex vivo*.
(a-c) Stainings of human liver sections from the experiment shown in Fig. 5f. **(a)** Hematoxylin and eosin (H&E) staining. Scale bars: 100 µm. **(b)** Immunohistochemical staining for GFP. Scale bars: 100 µm. **(c)** Confocal images of tumour cells mechanically arrested in liver sinusoids, stained for GFP (ezrin, green), vimentin (red) and DAPI (blue). Scale bar:50 µm. **(d)** Confocal images of tumour cells attached to liver vessel walls, stained for GFP (ezrin, green), vimentin (red) and DAPI (blue) showing the varying orientations towards the attachment site (top and middle panel) or to the distal side (lower panel), as quantified in Figure 5f. Scale bars: 50 µm (overview images) or 10 µm (detail).

Supplementary Figure 8

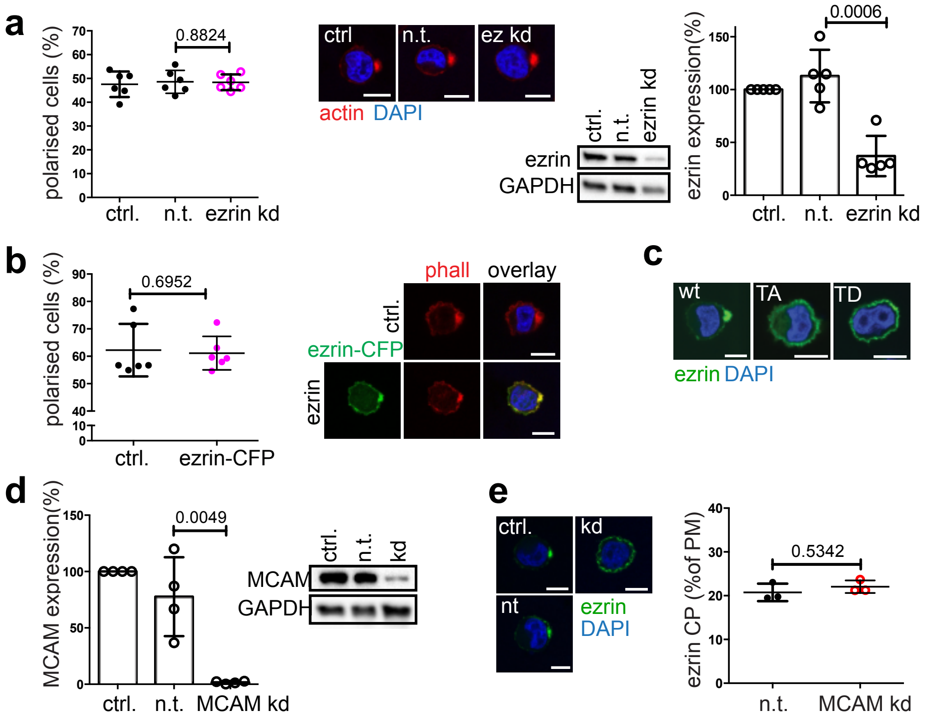

Effect of MCAM expresion levels on sc polarity.
(a) Polarity assays and representative images of SkMel2 cells expressing non-targeting (n.t.) or ezrin-targeting (ezrin kd) siRNAs stained with phalloidin showing the fraction of actin polarised cells (n=6, mean ± SD, paired t-tests). Western blot of ezrin expression (n=5, mean ± SD, unpaired t-test).
(b) Polarity assays and representative images of SkMel2 cells expressing CFP (ctrl.) or ezrin-CFP stained with phalloidin showing the fraction of actin polarised cells (n=6, mean ± SD, paired t-test). Ezrin expression was evaluated by visual inspection of CFP fluorescence.
(c) Representative confocal images of SkMel2 cells expressing GFP-labelled wildtype (wt)ezrin, ezrin-TA or ezrin-TD.
(d) Western blot of MCAM expression in MCAM kd SkMel2 cells (n=4, mean ± SD, unpaired t-test).
(e) Representative confocal images of SkMel2 MCAM kd cells expressing ezrin-GFP. The average fluorescence intensity was quantified (right) in the cytoplasma and at the PM. Average ezrin intensity in the CP per ezrin intensity at the PM of 25-30 cells is shown for each independent experiment (n= 3, mean ± SD, unpaired t-test). Scale bars:10µm.

Supplementary Figure 9

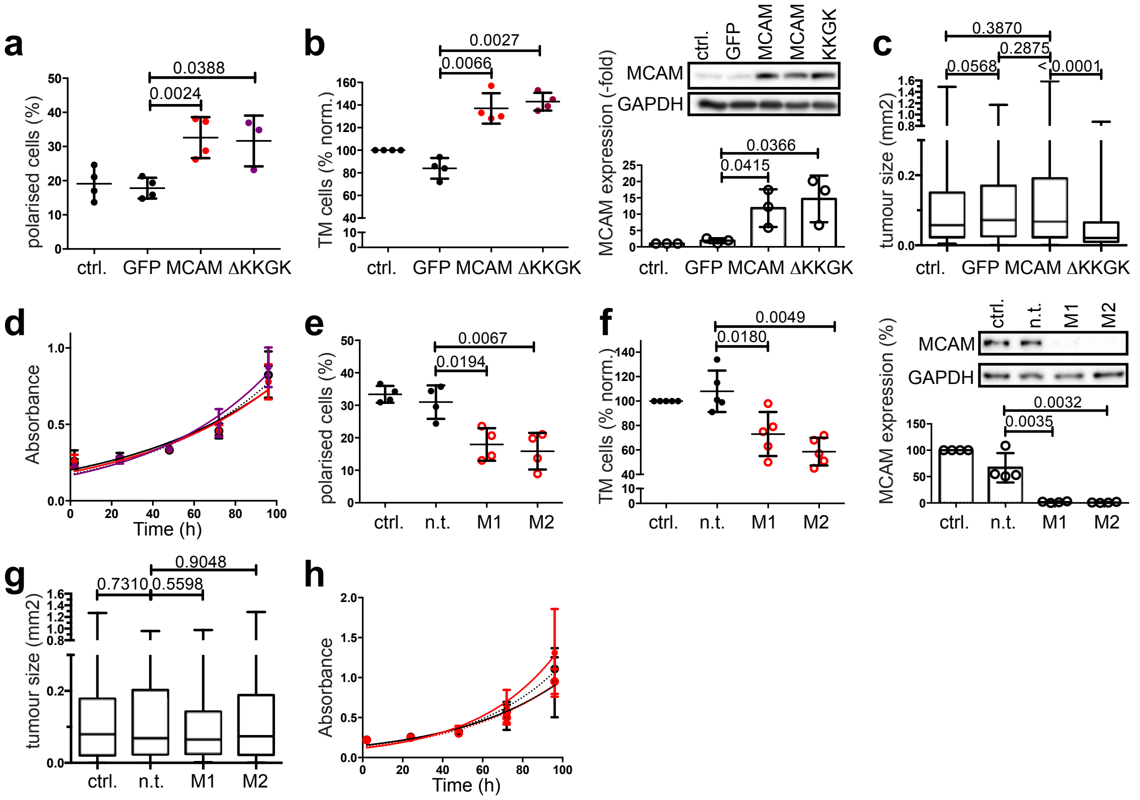

Effect of MCAM expresion levels *in vivo*.
(a) Polarity assays (fraction of polarised cells) of B16-F0 cells used in the experiments shown in Fig. 7a and 7b transfected with ezrin-mCherry (n=4, mean ± SD, paired t-test).
(b) Transmigration assays of B16-F0 cells used in the in vivo experiments shown in Fig. 7a and 7b showing the number of transmigrated cells after 24 hours normalized to the untransfected control (n=4, mean ± SD, paired t-tests). Western blot of Mcam expression (mean ± SD, unpaired t-tests, n=3).
(c) Size of tumour nodules on mouse lungs from the experiment shown in Fig. 7a. (n > 180, represented as box-and-whisker plots, Mann-Whitney tests).
(d) In vitro cell Proliferation assay (xtt) of the cells used for the in vivo experiments shown in Fig. 7a and 7b. (n=3, mean ± SD). The lines show fitted exponential growth curves for B16-F0 cells expressing no construct (ctrl., black), GFP (black, dotted), Mcam (red) or McamΔ KKGK (ΔKKGK, purple).
(e) Polarity assays (fraction of polarised cells) of B16-F1 cells used in the experiments shown in Fig. 7c and 7d transfected with ezrin-mCherry (n=4, mean ± SD, paired t-tests).
(f) Transmigration assays of B16-F1 cells used in the in vivo experiments shown in Fig. 7c and 7d showing the number of transmigrated cells after 24 hours normalized to the untransfected control (n=5, mean ± SD, paired t-tests). Western blot of Mcam expression (mean ± SD, unpaired t-tests, n=4).
(g) Size of tumour nodules on mouse lungs from the experiment shown in Fig. 7c (n > 180, represented as box-and-whisker plots, Mann-Whitney tests).
(h) In vitro cell Proliferation assay (xtt) of the cells used for the experiments shown in Fig. 7c and 7d. (n=3, mean ± SD). The lines in proliferation assays show fitted exponential growth curves for B16-F1 cells expressing no shRNA (ctrl., black), non-targeting (n.t., black, dotted) or Mcam-targeting (MCAM kd M1, red, dotted and MCAM kd M2, red, solid) shRNAs.

Supplementary Figure 10

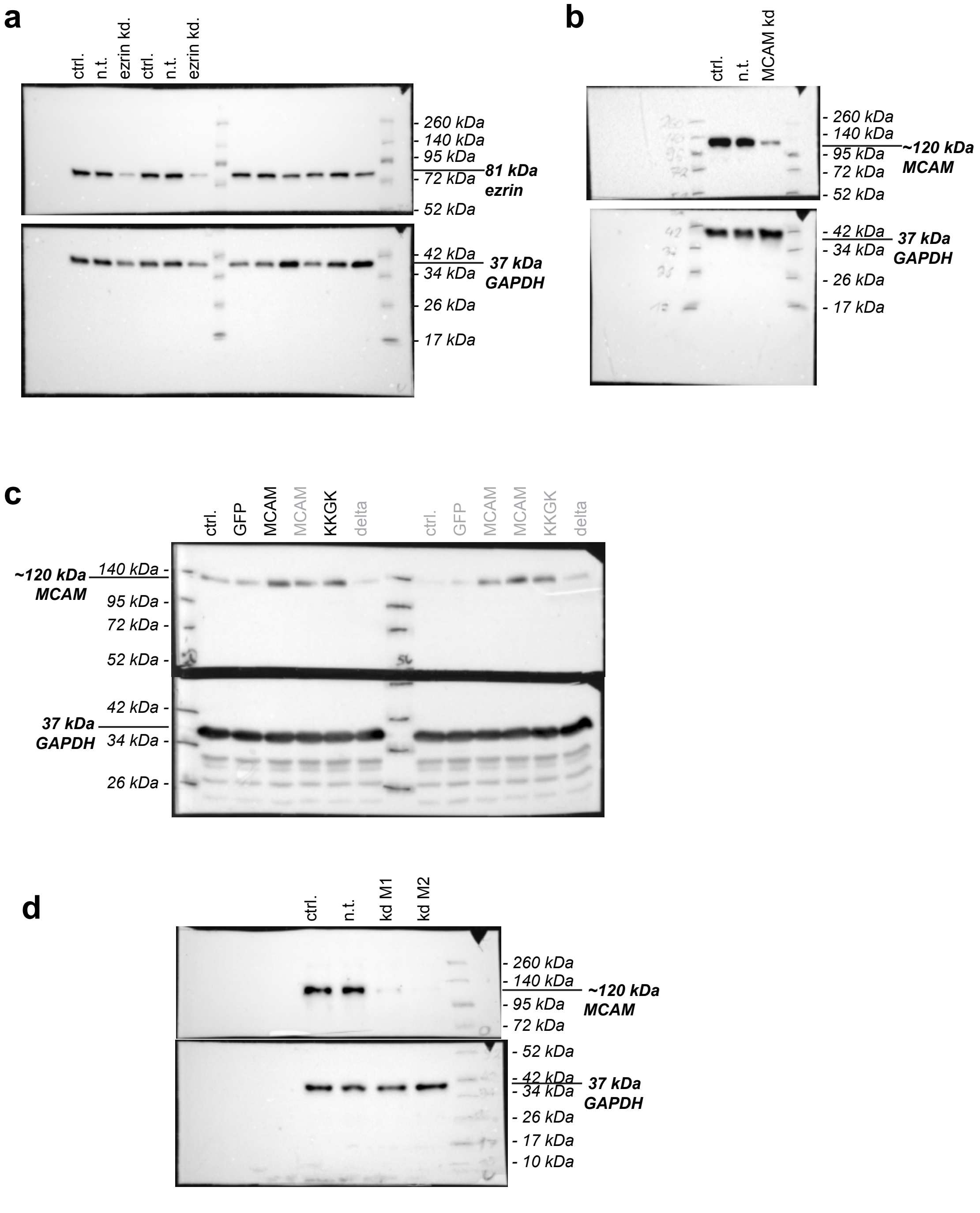

Western Blots shown in Supplementary Fig. 8 and 9.
(a) Original Western Blots shown in Supplementary Figure 8a.
(b) Original Western Blots shown in Supplementary Figure 8d.
(c) Original Western Blots shown in Supplementary Figure 9b.
(d) Original Western Blots shown in Supplementary Figure 9f.

Supplementary Figure 11

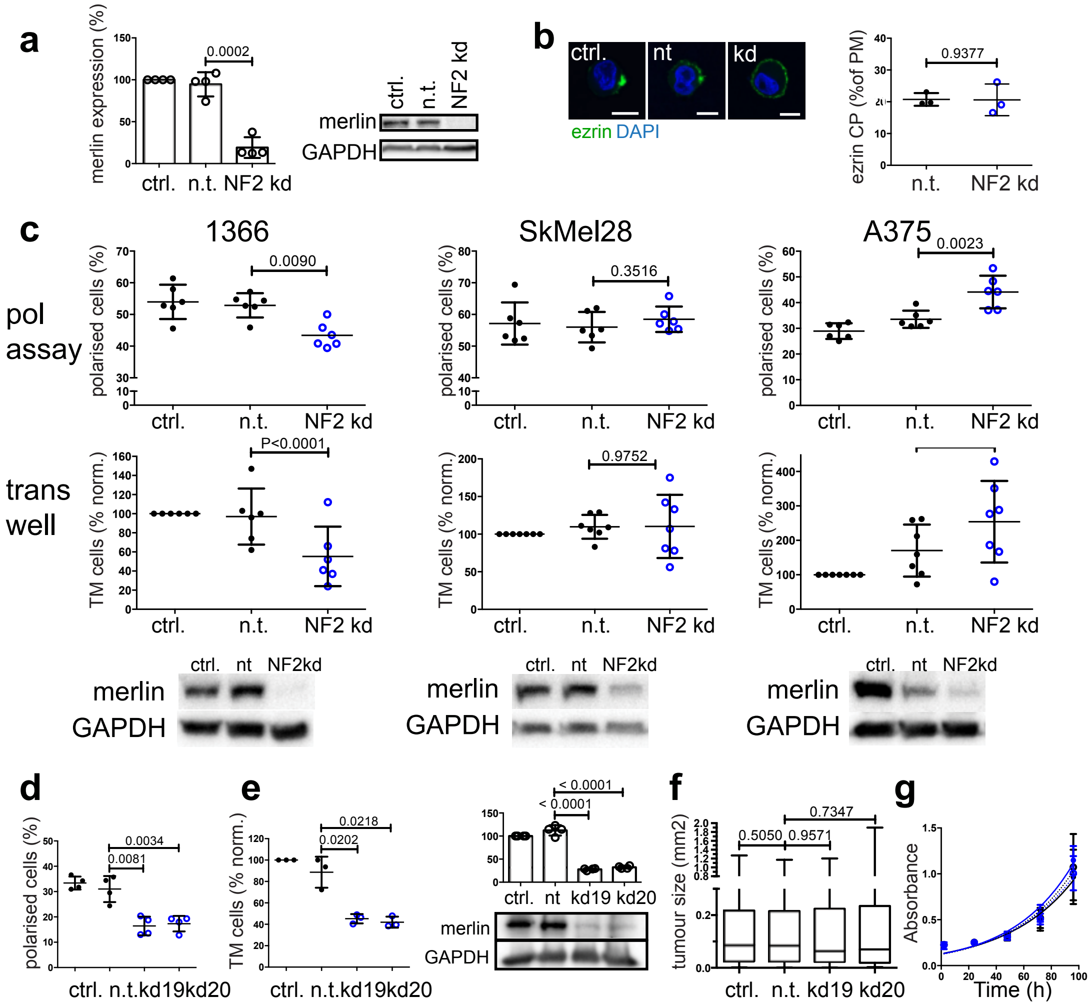

Effect of merlin expresion levels.

(a) Merlin Western blot of SkMel2 NF2 kd cells (mean ± SD, unpaired t-test, n=4). (b) Representative confocal images of SkMel2 NF2 kd cells expressing ezrin-GFP. Scale bars:10µm. The average fluorescence intensity was quantified (right) in the cytoplasma and at the PM. Average ezrin intensity in the CP per ezrin intensity at the PM of 25-30 cells is shown for each independent experiment (n=3, mean ± SD, unpaired t-test). (c) Polarity assays (top row), transmigration assays (middle row) and merlin Western Blots (bottom row) of WM1366 (left), SkMel28 (middle) and A375 (right) human melanoma cells untransfected (ctrl.), expressing non-targeting (n.t.) or NF2-targeting (NF2 kd) siRNA pools (n=6-7, mean ± SD, paired t-test for polarity assays, 1-sample t-test to 100 for transmigration assays .

(d) Polarity assays (fraction of polarised cells) of B16-F1 cells used in the in vivo experiment shown in Fig. 8e transfected with ezrin-mCherry. (n=4, mean ± SD, paired t-test).

(e) Transmigration assays of B16-F1 cells used in the in vivo experiment shown in Fig. 8e showing number of transmigrated cells after 24 hours normalised to untransfected control. (n=3, mean ± SD, 1-sample t-test to 100). Western blot of merlin expression (mean ± SD, unpaired t-tests, n=4).

(f) Size of tumour nodules on mouse lungs from the experiment shown in Fig. 8e. (n>180, represented as box-and-whisker plots, Mann-Whitney tests).

(g) In vitro cell Proliferation assay (xtt) of the cells used for the experiment shown in Fig. 8e. (n=3, mean ± SD). The lines show fitted exponential growth curves for B16-F1 cells expressing no shRNA (ctrl., black), non-targeting (n.t., black, dotted) or Nf2-targeting (NF2 kd 19, blue, dotted and NF2 kd 20, blue, solid) shRNAs.

Supplementary Figure 12

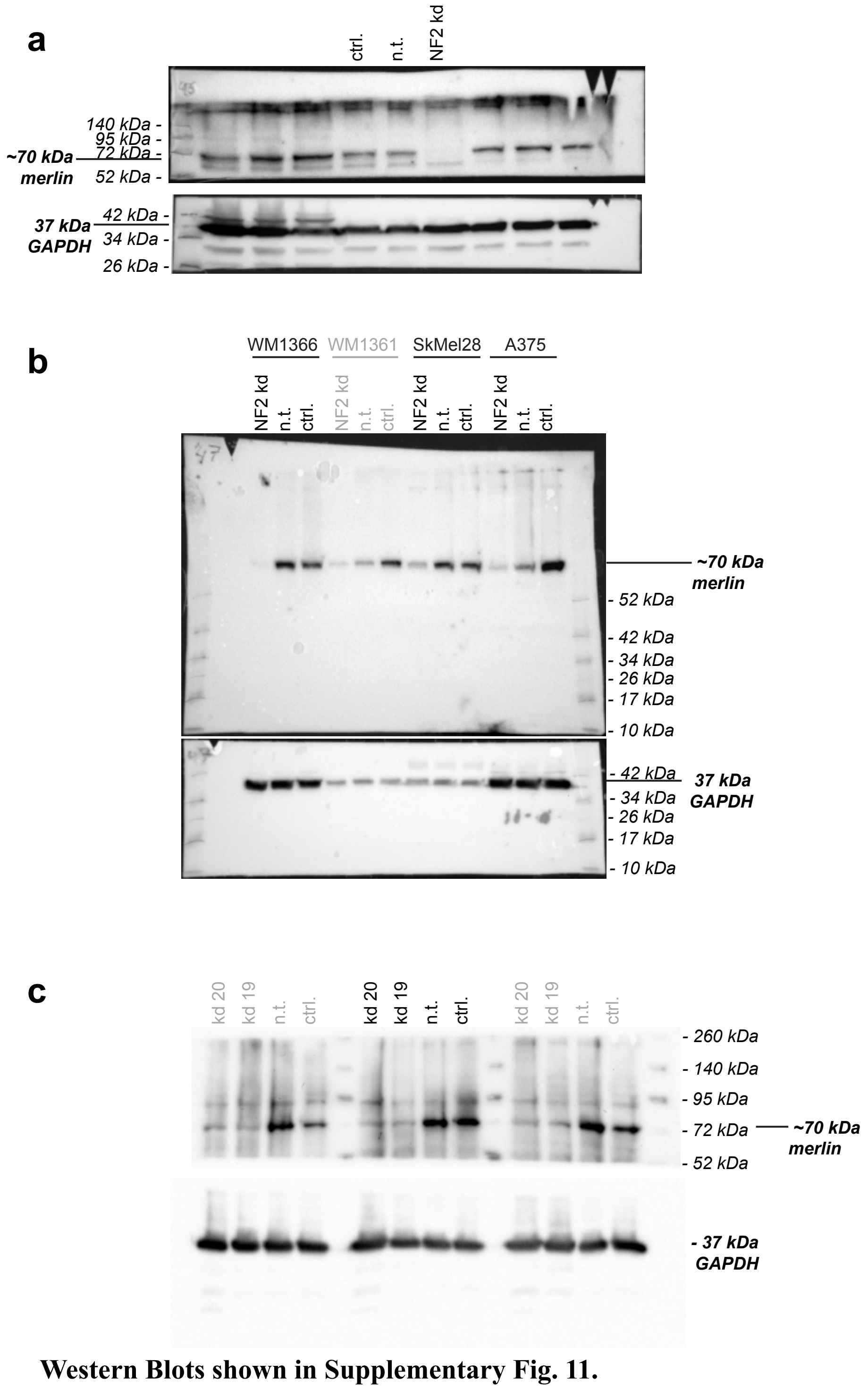

Western Blots shown in Supplementary Fig. 11.
(a) Original Western Blots shown in Supplementry Figure 11a.
(b) Original Western Blots shown in Supplementry Figure 11c.
(c) Original Western Blots shown in Supplementry Figure 11e.

Supplementary Figure 13

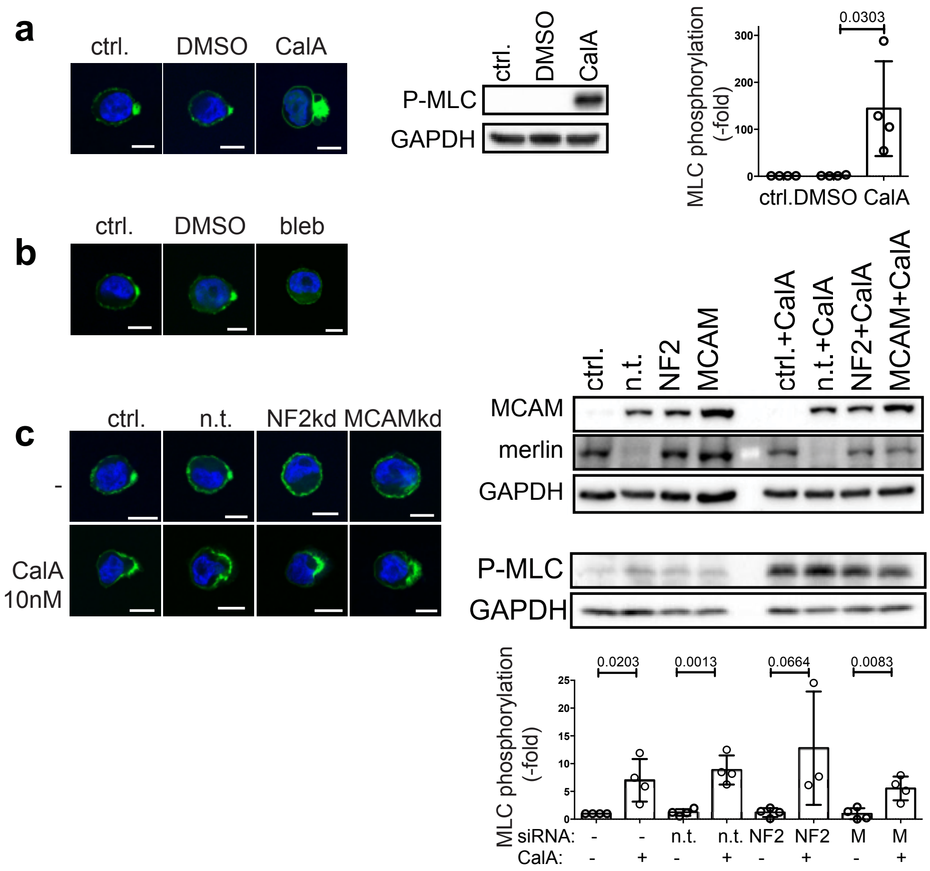

Effect of MLC phosphorylation on sc polarity.

(a) Representative confocal images of ezrin-GFP and Western blots of MLC phosphorylation of cells used in Fig. 8h (mean ± SD, unpaired t-test, n=4).

(b) Representative confocal images of ezrin-GFP in cells used in Fig. 8j.

(c) Representative confocal images of ezrin-GFP and Western blots of MLC phosphorylation (mean ± SD, unpaired t-test, n=4) of cells untreated or treated with 10nM calicylin A used in Fig. 8m. Western blots of MLC phosphorylation, MCAM (M) and Merlin expression (mean ± SD, unpaired t-tests, n=4).

Supplementary Figure 14

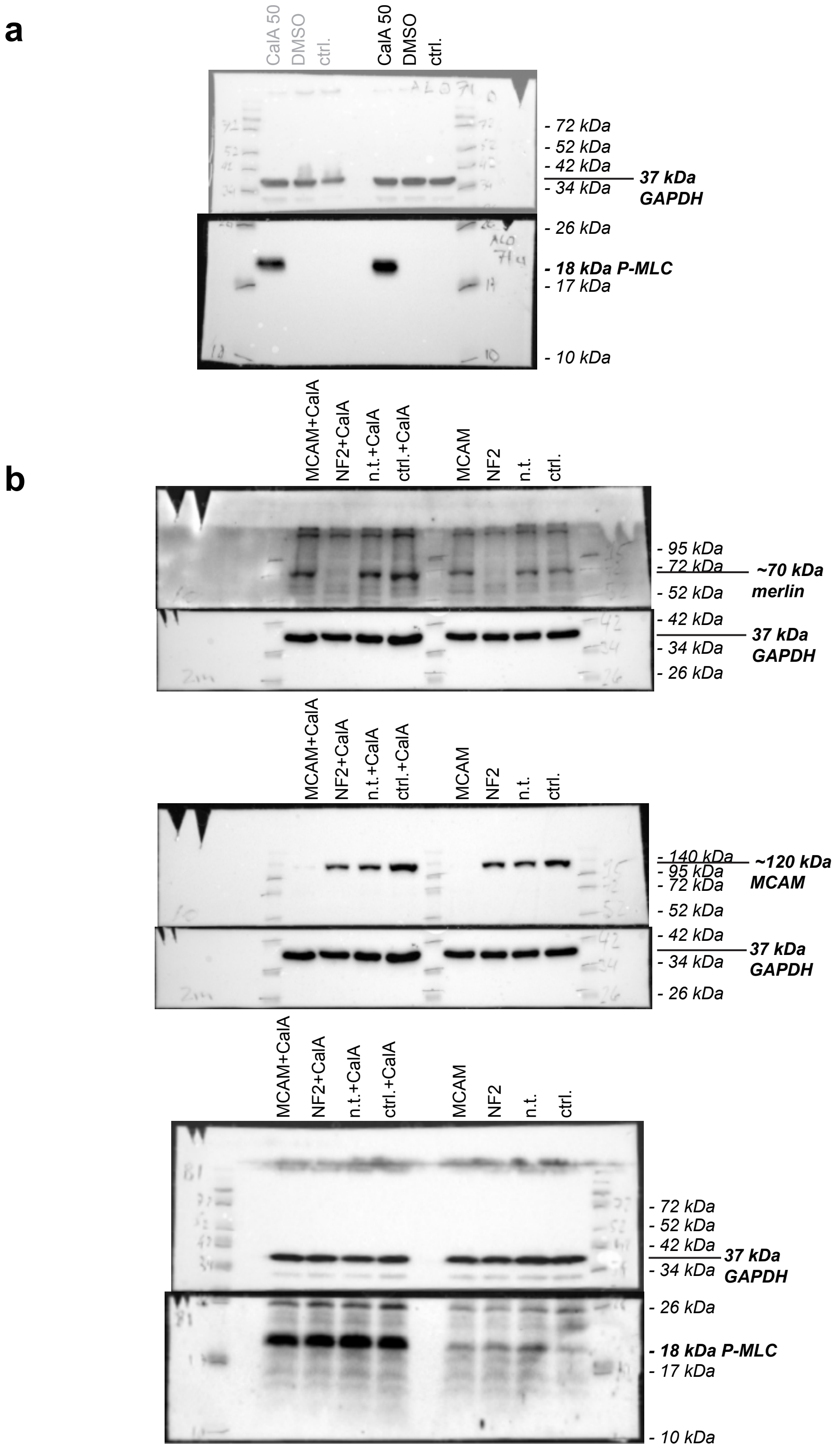

Western Blots shown in Supplementary Fig. 13.

(a) Original Western Blots shown in Supplementary Figure 13a.

(b) Original Western Blots shown in Supplementary Figure 13c.

Supplementary Figure 15

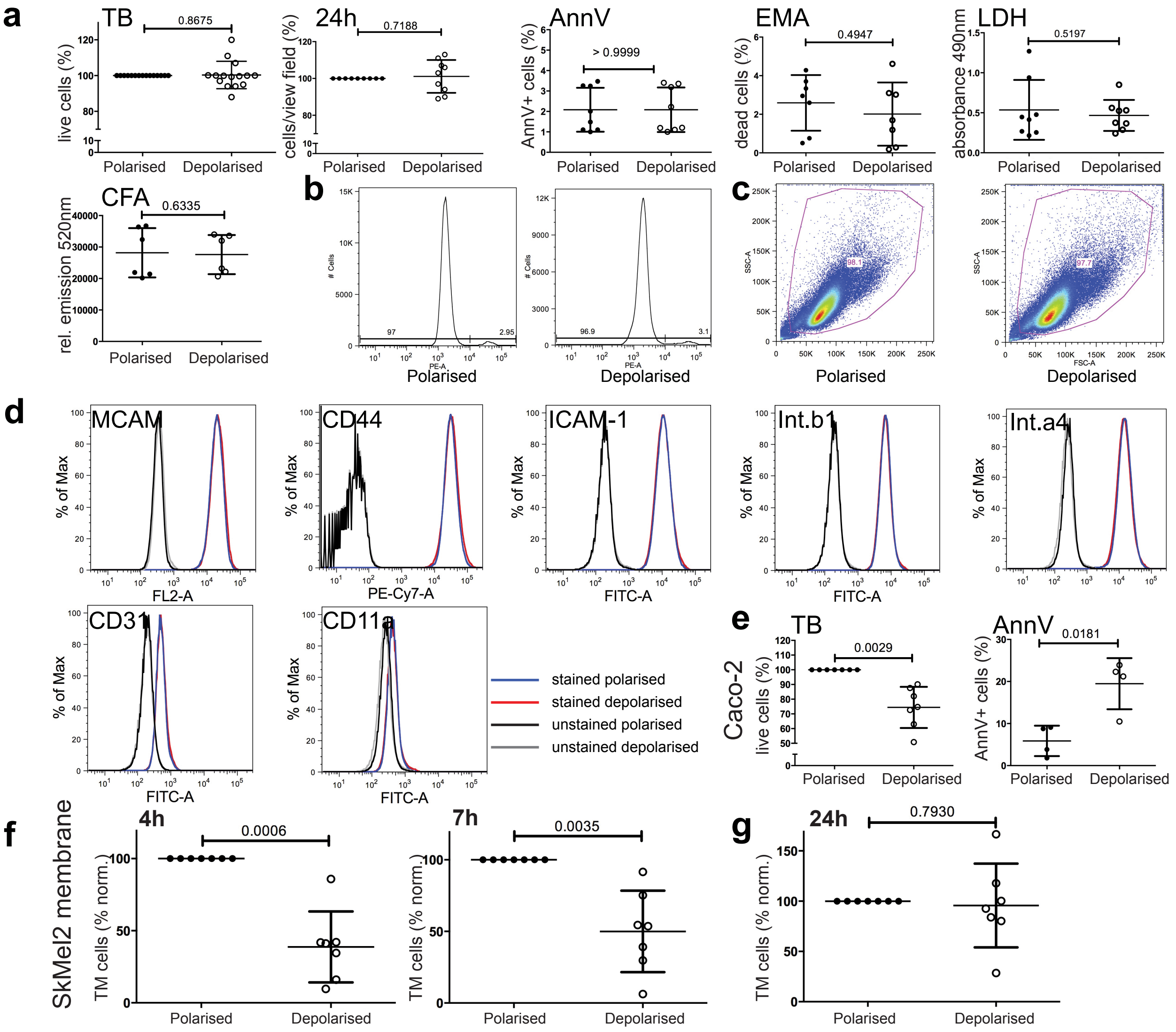

Generic depolarisation of SkMel2 cells.

(a) Cell viability of polarised (30min) and depolarised (3h) SkMel2 cells measured by (left to right) number of viable cells after Trypan blue staining (TB, n=15), number of cells 24h after plating (24h, n=9), FACS analysis of AnnexinV staining (AnnV, n=8), FACS analysis of EMA live-dead staining (EMA, n=7), cell death by LDH assay (LDH, n=8) and anchorage-inpendent growth in soft agar colony-formation assay (CFA, n=6). (mean ± SD, 1-sample t-test to 100 (TB, 24h) or unpaired t-test (AnnV, EMA, LDH, CFA)).

(b) Histograms of a representative EMA live-dead measurement for polarised and depolarised SkMel2 cells from the experiment shown in (a).

(c) Density plots of a representative EMA live-dead measurement for polarised and depolarised SkMel2 cells from the experiment shown in (a).

(d) Histograms of FACS analysis of expression of MCAM, CD44, ICAM-1, β1-Integrin (Int. β1), α4-Integrin (Int.α4), CD31 and CD11a (not expressed) on polarised (30min, blue) and depolarised (3h, red) SkMel2 cells. Unstained controls are shown in black (polarised) and grey (depolarised). One representative histogram of at least three independent measurements is shown. The same gating strategy was used for all measurements and is shown in Supplementary Fig. 16.

(e) Control for (a) in detachment-sensitive Caco-2 cells. Viable cells after Trypan blue staining (TB, n=7) and FACS analysis of AnnexinV staining (AnnV, n=4). (mean ± SD, 1-sample t-test to 100 (TB) or unpaired t-test (AnnV)).

(f, g) Transmigration assays of polarised (30min) and depolarised (3h) SkMel2 cells through transwell mebrane showing the fraction of transmigrated cells after 4 hours (f, left), 7 hours (f, right) or 24 hours (g) (n=7, mean ± SD, 1-sample t-tests compared to 100).

Supplementary Figure 16

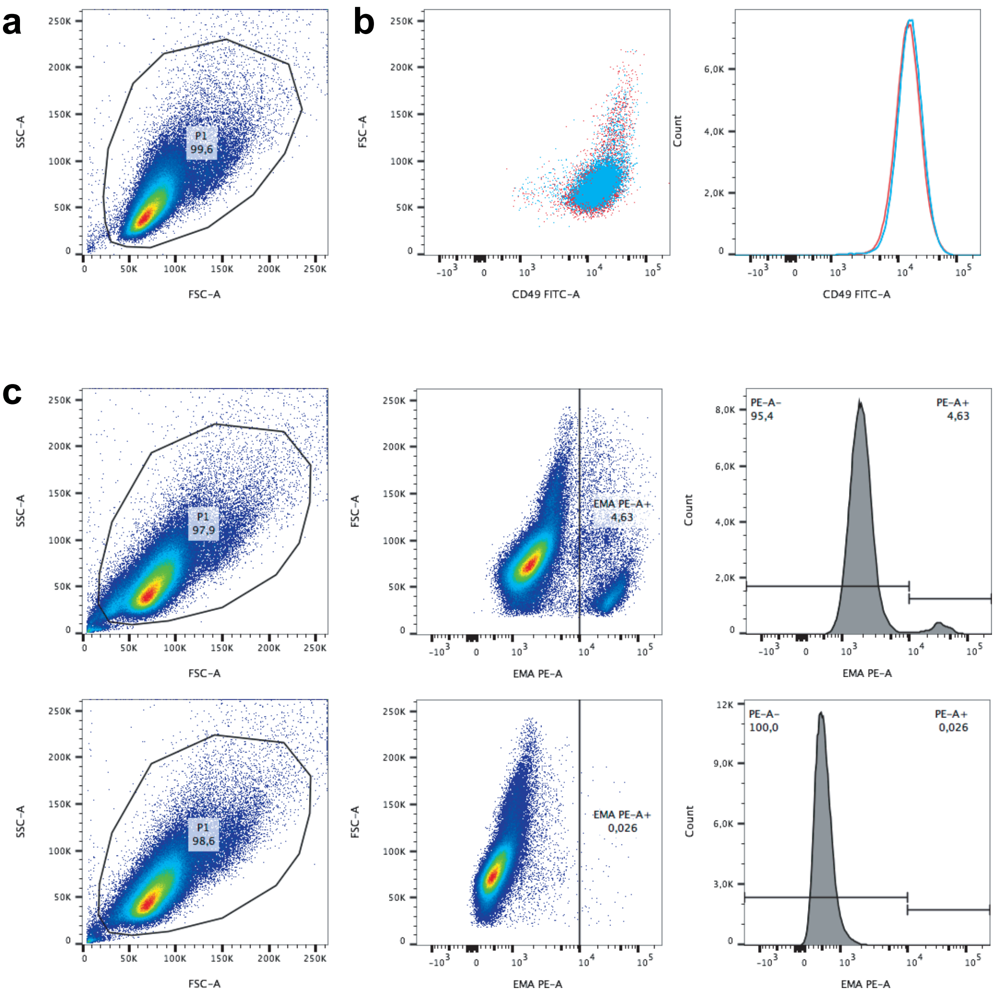

Gating strategies for SkMel2 FACS experiments shown in Supplementary Fig. 15.

(a) Gating strategy for comparative surface marker analysis of polarised or depolarised cells. One representative sample of SkMel2 cells is shown.
(b) Representative CD49d surface marker comparison of polarised (blue) and depolarised (red) cells shown as dot-plot (left) or histogram (right). One representative sample of SkMel2 cells is shown.
(c) Gating strategy for live/dead (EMA) and AnnexinV staining. One representative sample of SkMel2 cells is shown. Shown are plots of stained cells (top row) as well as unstained control (bottom row). Left plots show an ungated plot with gate P1 used to gate out cell debris. Cells were then distinguished according to their EMA staining as PE-A+ (EMA+) or PE-A- (EMA-) shown here as dot-plot (middle column) or histogram (right column).

Supplementary Figure 17

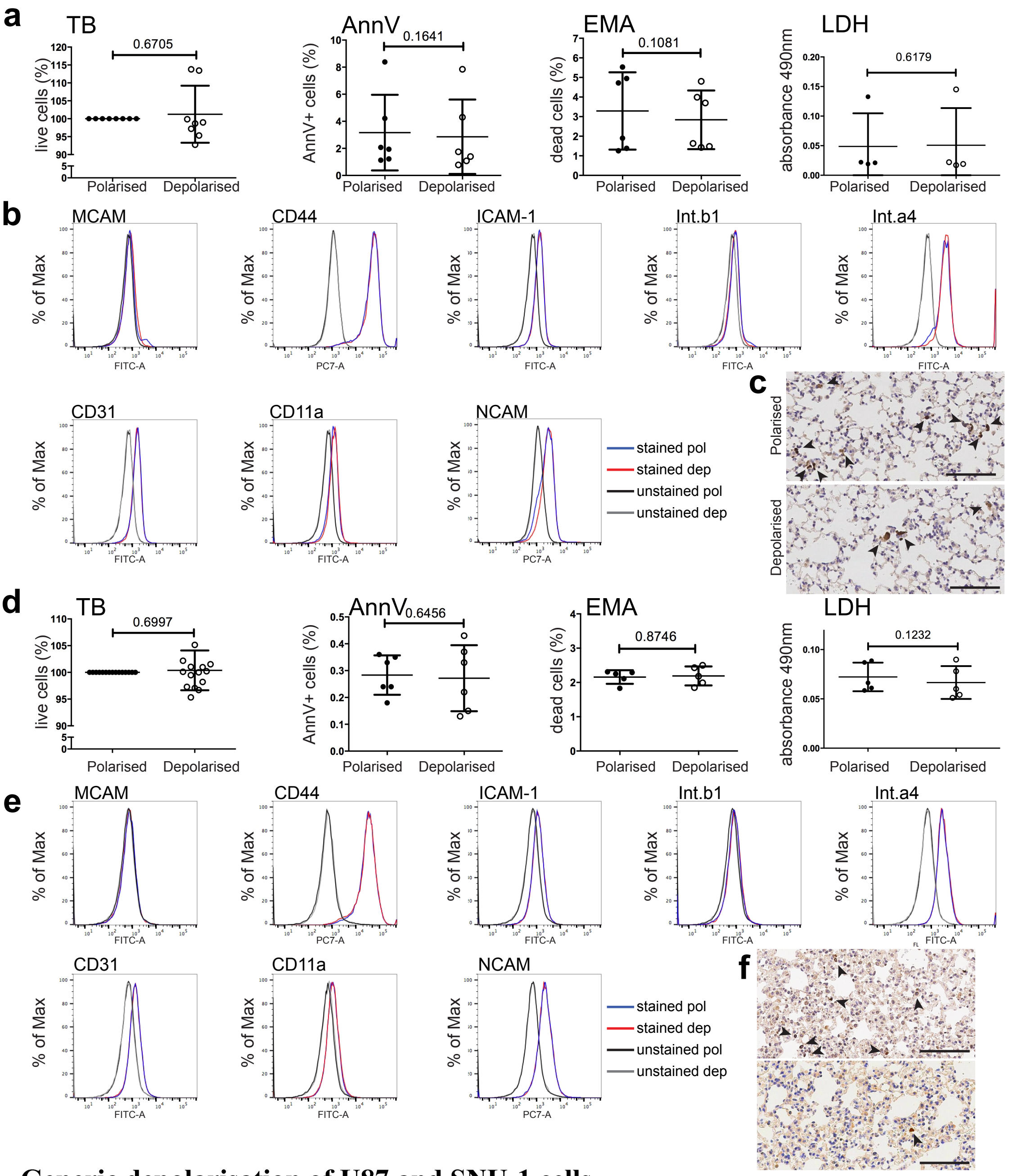

Generic depolarisation of U87 and SNU-1 cells.

(a, d) Cell viability of polarised (30min) and depolarised (3h) U87 **(a)** and SNU-1 **(d)** cells measured by (from left to right) number of viable cells after Trypan blue staining (TB), FACS analysis of AnnexinV staining (AnnV), FACS analysis of EMA live-dead staining (EMA) and cell death by LDH assay (LDH). (n=4-14, mean ± SD, 1-sample t-test compared to 100 (TB) or unpaired t-test (AnnV, EMA, LDH)).

(b, e) Histograms of FACS analyses of expression of MCAM, CD44, ICAM-1, β 1-Integrin (Int. β1), α4-Integrin (Int.α4), CD31, CD11a and NCAM on polarised (30min, blue) and depolarised (3h, red) U87 **(b)** and SNU-1 **(e)** cells. Unstained controls are shown in black (polarised) and grey (depolarised). One representative histogram of at least three independent measurements is shown. The gating strategy was the same as for SkMel2 cells and is shown in Supplementary Fig. 16.

(c, f) Representative images of GFP-stained 2μm sections of mouse lungs 30 minutes after injection with polarised (30min) or depolarised (3h) U87 **(c)** and SNU-1 **(f)** cells expressing GFP. Scale bars: 100μm.

Supplementary Figure 18

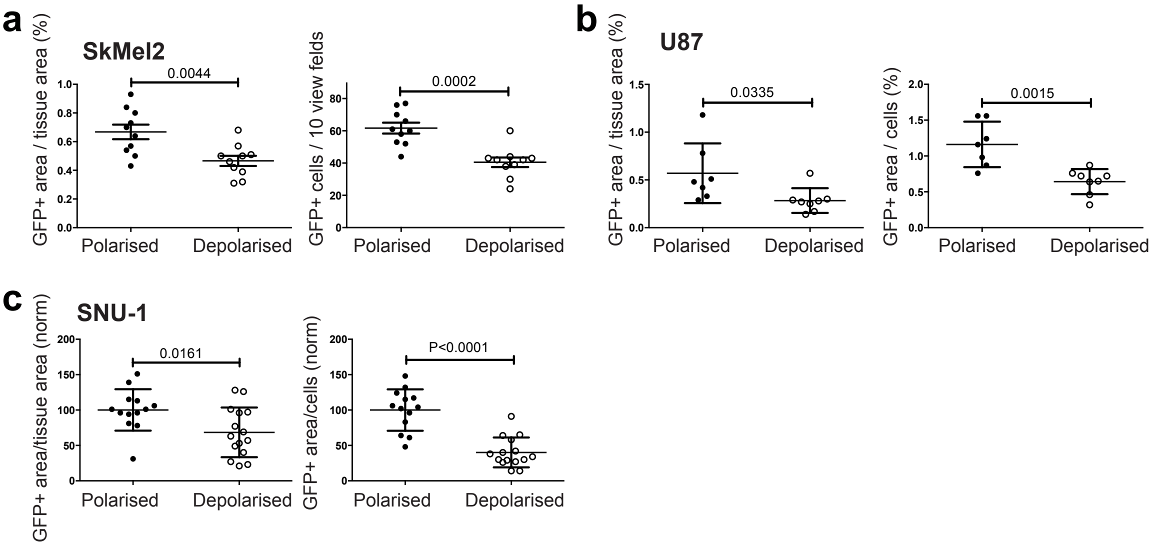

Seeding after generic depolarisation *in vivo.*
(a) Quantification of seeding of GFP-positive SkMel2 cells from histological slides as shown in Fig. 9j by GFP-positive area per tissue area or by GFP-positive cells per 10 random view fields. (n=10, mean ± SD, unpaired t-tests). Sections from the same mice as in Fig. 9k were quantified.
(b) Quantification of seeding of GFP-positive U87 cells from histological slides as shown in Supplementary Data Fig. 17c by GFP-positive area per tissue area or by GFP-positive cells per total number of cells. (n=7-8, mean ± SD, unpaired t-tests). Sections from the same mice as in Fig. 9l were quantified.
(c) Quantification of seeding of GFP-positive SNU-1 cells from histological slides as shown in Supplementary Data Fig. 17f by GFP-positive area per tissue area or by GFP-positive cells per total number of cells. (n=13-15, mean ± SD, unpaired t-tests). Sections from the same mice as in Fig. 9m were quantified.

Supplementary Figure 19

a

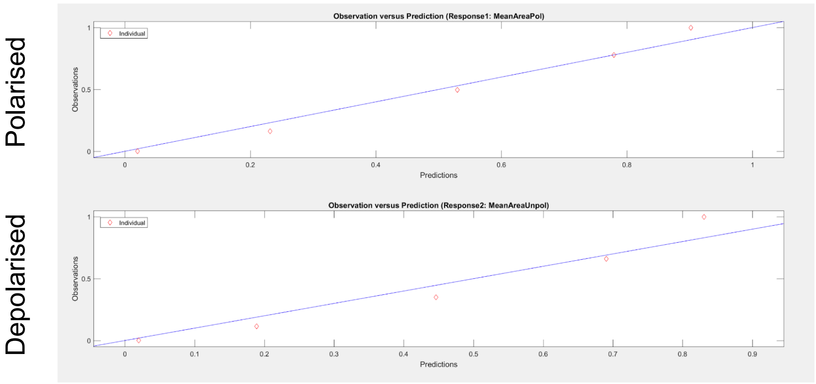

b

	Polarised	Depolarised
Attachment Rate	10.74 $\%.\ s^{-1}$	3.81 $\%.\ s^{-1}$
Adherence Rate	3.95 $\%.\ s^{-1}$	3.02 $\%.\ s^{-1}$
Tissue Residence Ra	0.06 $\%.\ s^{-1}$	0.05 $\%.\ s^{-1}$

c

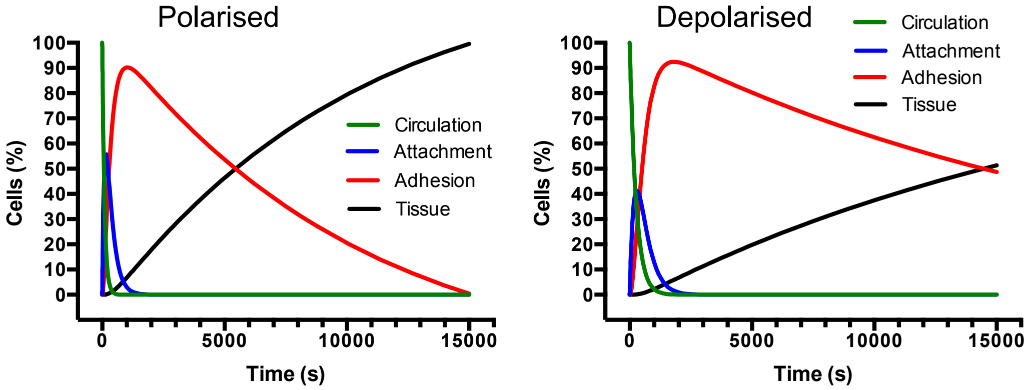

In silico modelling of attachment, adhesion and tissue residence.
(a) Comparison of observation versus prediction. Fitting of the compartmental model to the data lies within 95% confidence limits for all parameters.
(b) Transfer rates between compartments obtained from the compartmental model shown in Figure 10a.
(c) Changes in the circulating, attached, adhered and tissue-resident populations of polarised (left) and depolarised (rigt) cells. Comparisons for attachment, adhesion and tisue residence between polarised and depolarised cells are shown in Figure10b-e.

Supplementary Table 1

cell line	polarisation 10 min (%)	polarisation 6h (%)	polarisation 10 d (%) [1]
SkMel2	72.8 (+/-5.6)	38.4 (+/-2.8)	23.4 (+/-5.6)
A375	51.2 (+/-3.8)	44.1 (+/-2.8)	49.9 (+/-1.5)
SkMel28	84.1 (+/-3.2)	22.7 (+/-6.0)	16.3 (+/-1.0)
WM1361	1.3 (+/-1.2)	3.2 (+/-1.0)	n.d. [2]
WM1366	59.9 (+/-2.9)	29.5 (+/-8.1)	12.2 (+/-4.9)
Caco-2	36.0 (+/-8.2)	16.4 (+/-2.8)	n.d. [2]
HeLa	31.0 (+/-4.6)	7.1 (+/-2.2)	25.3 (+/-2.3)
HepG2	42.7 (+/-7.2)	11.9 (+/-3.1)	70.1 (+/-7.5)
MCF7	14.5 (+/-8.2)	5.8 (+/-1.7)	7.7 (+/-1.9)
DLD1	9.4 (+/-2.7)	6.1 (+/-5.4)	13.9 (+/-2.3)
MDA-MB-231	24.1 (+/-7.3)	10.1 (+/-4.1)	n.d. [2]

[1] Cells singularized by trypsinisation
[2] no growth on poly-HEMA coated dishes

Ezrin polarisation in tumour cell lines
Polarisation of ezrin-GFP in cell lines shown in Figure 3a measured at 10 minutes, 6 hours and 10 days after detachment.

Supplementary Table 2

name. entity Figure	isolated from	pos. cells markers:number	pol. cells	neg. cells markers: number	CD45+ cells	isolation identification	cells before staining
CTC(MC) 1 Fig.3 Sup.Fig.2	mammary carcinoma blood	CK+CD45-ezrin+: 66	15	CK-CD45-: 33	0	CellSearch, staining, aCGH	~ 500
CTC(MC) 1 Sup.Fig.2	mammary carcinoma blood	CK+CD45-MCAM+: 4	2	CK-CD45-: 0	0	CellSearch, staining, aCGH	~ 60
CTC(MC) 1 Sup.Fig.2	mammary carcinoma blood	CK+phall+clCasp.3-:15	5	CK-CD45-: 0	0	CellSearch, staining, aCGH	~ 60
CTC(MC) 1 Sup.Fig.2	mammary carcinoma blood	CK+CD45-β1-Integrin+: 21	8	CK-CD45-: 0	0	CellSearch, staining, aCGH	~ 200
CTC(MC) 2 Fig.2 Sup.Fig.3	mammary carcinoma blood	CK+CD45-ezrin+: 11	4	CK-CD45-: n.d.	1	CellSearch, staining	~135
CTC(SCLC) Sup. Fig.2	small cell lung cancer blood	CK+CD45-ezrin+: 4 (cluster of 4 cells)	2	CK-CD45-: 0	0	CellSearch, staining	~ 200
PE(PC) 1 Fig.2 Sup.Fig.2	pancreatic carcinoma pleural effusion	EpCAM+CD45-ezrin+: 11	3	EpCAM-CD45-: 25	1	CD45 depletion	>500
PE(PC) 1 Sup.Fig.2	pancreatic carcinoma pleural effusion	EpCAM+CD45-CD44+: 4	2	EpCAM-CD45-: n.d.	0	CD45 depletion	>500
PE(MC) 1 Sup.Fig.2	mammary carcinoma pleural effusion	EpCAM+CD45-ezrin+: 59	7	EpCAM-CD45-: n.d.	0	CD45 depletion, FACS	>500
PE(MC) 2 Sup.Fig.2	mammary carcinoma pleural effusion	EpCAM+CD45-ezrin+: 8	2	EpCAM-CD45-: n.d.	0	CD45 depletion, FACS	>500
AE(MC) 1 Fig.3	mammary carcinoma ascites	EpCAM+CD45-ezrin+: 18	5	EpCAM-CD45-: n.d.	0	CD45 depletion, FACS	>500

Liquid phase samples used in this study

Liquid phase samples isolated from patients, showing tumour entity, liquid phase, number of cells positive for positive markers, number of polarised cells out of positive cells, number of cells negative for positive markers, number of CD45+ cells, methods of isolation and identification and number of cells per sample prior to staining and processing for microscopy.

Supplementary Table 3

prim or met	localisation of metastases	MCAM intensity	MCAM % of pos. tumour cells	MCAM Gish score	MCAM high/low
prim		2	4	8	2
prim		0	0	0	1
prim		0	0	0	1
prim		2	4	8	2
prim		1	1	1	1
prim		1	1	1	1
prim		1	1	1	1
prim		0	0	0	1
prim		0	0	0	1
prim		2	4	8	2
prim		1	4	4	2
prim		2	3	6	2
prim		1	3	3	1
prim		0	0	0	1
prim		0	0	0	1
prim		0	0	0	1
prim		0	0	0	1
prim		0	0	0	1
prim		0	0	0	1
prim		2	3	6	2
prim		0	0	0	1
prim		2	3	6	2
prim		0	0	0	1
prim		0	0	0	1
prim		0	0	0	1
prim		2	4	8	2
prim		2	4	8	2
prim		1	1	1	1
prim		0	0	0	1
prim		0	0	0	1
prim		0	0	0	1
prim		2	4	8	2
prim		0	0	0	1
prim		0	0	0	1
prim		1	1	1	1
prim		0	0	0	1
prim		0	0	0	1
prim		2	2	4	2
prim		0	0	0	1
prim		0	0	0	1
prim		0	0	0	1
prim		2	3	6	2
prim		0	0	0	1
prim		0	0	0	1
prim		2	4	8	2
prim		2	4	8	2
prim		0	0	0	1
prim		2	2	4	2
prim		2	4	8	2
prim		0	0	0	1
prim		2	4	8	2
prim		0	0	0	1
prim		2	3	6	2
prim		2	3	6	2
prim		1	3	3	1
prim		2	3	6	2
prim		2	3	6	2
prim		2	4	8	2
prim		1	3	3	1
prim		0	0	0	1
prim		0	0	0	1
prim		2	3	6	2
prim		0	0	0	1
prim		0	0	0	1

prim or met	localisation of metastases	MCAM intensity	MCAM % of pos. tumour cells	MCAM Gish score	MCAM high/low
prim		0	0	0	1
prim		0	0	0	1
prim		2	3	6	2
prim		0	0	0	1
prim		2	1	2	1
prim		0	0	0	1
met	Adrenal gland	2	3	6	2
met	Bone	2	4	8	2
met	Brain	1	2	2	1
met	Brain	2	3	6	2
met	Brain	2	4	8	2
met	Brain	2	4	8	2
met	Brain	2	4	8	2
met	Brain	2	4	8	2
met	Brain	2	3	6	2
met	Brain	2	4	8	2
met	Brain	2	4	8	2
met	Brain	2	4	8	2
met	Brain	2	2	4	2
met	Brain	2	4	8	2
met	Brain	0	0	0	1
met	Brain	2	1	2	2
met	Brain	0	0	0	1
met	Brain	1	1	1	1
met	Brain	0	0	0	1
met	Brain	2	2	4	2
met	Brain	2	3	6	2
met	Brain	2	3	6	2
met	Brain	2	4	8	2
met	Brain	0	0	0	1
met	Brain	0	0	0	1
met	Brain	2	4	8	2
met	Brain	0	0	0	1
met	Brain	2	2	4	2
met	Brain	2	3	6	2
met	Brain	2	4	8	2
met	Brain	2	3	6	2
met	Brain	2	4	8	2
met	Brain	2	1	2	1
met	Brain	0	0	0	1
met	Brain	2	3	6	2
met	Brain	0	0	0	1
met	Brain	2	3	6	2
met	Brain	2	4	8	2
met	Brain	2	3	6	2
met	Brain	0	0	0	1
met	Brain	2	2	4	2
met	Brain	2	3	6	2
met	Brain	0	0	0	1
met	Brain	2	4	8	2
met	Brain	2	4	8	2
met	Brain	0	0	0	1
met	Brain	2	4	8	2
met	Brain	2	4	8	2
met	Brain	2	3	6	2
met	Brain	0	0	0	1
met	Brain	0	0	0	1
met	Brain	0	0	0	1
met	Brain	2	3	6	2
met	Brain	2	4	8	2
met	Brain	2	4	8	2
met	Brain	0	0	0	1
met	Brain	2	4	8	2
met	Brain	2	4	8	2

met	Brain	2	3	6	2
met	Brain	0	0	0	1
met	Brain	0	0	0	1
met	Brain	0	0	0	1
met	Brain	2	4	8	2
met	Brain	2	2	4	2
met	Brain	0	0	0	1
met	Brain	2	3	6	2
met	Brain	2	2	4	2
met	Brain	2	4	8	2
met	Brain	1	1	1	1
met	Brain	2	3	6	2
met	Brain	2	2	4	2
met	Brain	0	0	0	1
met	Brain	1	3	3	1
met	Brain	1	1	1	1
met	Brain	0	0	0	1
met	Brain	2	3	6	2
met	Brain	2	4	8	2
met	Brain	2	3	6	2
met	Brain	2	2	4	2
met	Brain	2	4	8	2
met	Brain	2	4	8	2
met	Brain	1	2	2	1
met	Brain	2	3	6	2
met	Brain	2	4	8	2
met	Brain	1	2	2	1
met	Brain	1	3	3	1
met	Brain	1	1	1	1
met	Brain	2	3	6	2
met	Brain	2	4	8	2
met	Brain	2	2	4	2
met	Brain	2	4	8	2
met	Brain	2	4	8	2
met	Brain	1	2	2	1
met	Brain	2	3	6	2
met	Brain	2	2	4	2
met	Brain	2	2	4	2
met	Brain	2	4	8	2
met	Brain	2	4	8	2
met	Brain	2	3	6	2
met	Brain	2	3	6	2
met	Brain	2	2	4	2
met	Brain	1	1	1	1
met	Brain	2	4	8	2
met	Brain	1	2	2	1
met	Brain	1	2	2	1
met	Brain	2	4	8	2
met	Brain	1	4	4	2
met	Brain	2	3	6	2
met	Brain	1	3	3	1
met	Brain	0	0	0	1
met	Brain	2	3	6	2
met	Brain	2	4	8	2
met	Brain	1	2	2	1
met	Brain	2	4	8	2
met	Brain	2	3	6	2
met	Brain	2	3	6	2
met	Brain	0	0	0	1
met	Brain	0	0	0	1
met	Brain	0	0	0	1
met	Breast	2	3	6	2
met	Intestine	2	2	4	2
met	Liver	0	0	0	1
met	Liver	0	0	0	1
met	Lung	2	4	8	2
met	Lung	2	4	8	2
met	Lung	0	0	0	1
met	Lung	0	0	0	1
met	Lung	2	4	8	2
met	Lung	2	4	8	2
met	Lymph node	2	2	4	2
met	Lymph node	1	2	2	1
met	Lymph node	2	4	8	2
met	Lymph node	2	4	8	2
met	Lymph node	1	2	2	1
met	Lymph node	2	3	6	2
met	Lymph node	1	2	2	1
met	Lymph node	2	2	4	2
met	Lymph node	2	4	8	2
met	Lymph node	2	2	4	2
met	Lymph node	2	3	6	2
met	Lymph node	2	4	8	2
met	Lymph node	0	0	0	1
met	Lymph node	2	4	8	2
met	Lymph node	2	4	8	2
met	Lymph node	2	4	8	2
met	Lymph node	2	3	6	2
met	Lymph node	2	4	8	2
met	Lymph node	2	1	2	1
met	Lymph node	2	2	4	2
met	Lymph node	0	0	0	1
met	Lymph node	2	2	4	2
met	Lymph node	0	0	0	1
met	Lymph node	2	4	8	2
met	Lymph node	2	4	8	2
met	Lymph node	2	3	6	2
met	Lymph node	2	1	2	1
met	Lymph node	2	3	6	2
met	Lymph node	0	0	0	1
met	Lymph node	0	0	0	1
met	Lymph node	2	3	6	2
met	Lymph node	2	3	6	2
met	Lymph node	0	0	0	1
met	Lymph node	2	1	2	1
met	Omentum majus	2	2	4	2
met	Parotis	1	3	3	1
met	Skeletal muscles	2	2	4	2
met	Skin	2	4	8	2
met	Skin	2	3	6	2
met	Skin	2	4	8	2
met	Skin	0	0	0	1
met	Skin	0	0	0	1
met	Skin	2	2	4	2
met	Skin	2	4	8	2
met	Skin	2	4	8	2
met	Skin	2	1	2	1
met	Skin	2	4	8	2
met	Skin	0	0	0	1
met	Skin	0	0	0	1
met	Skin	0	1	1	1
met	Skin	2	3	6	2
met	Skin	2	3	6	2
met	Skin	2	2	4	2
met	Skin	2	4	8	2
met	Skin	0	0	0	1
met	Skin	1	1	1	1
met	Skin	0	0	0	1
met	Skin	2	3	6	2
met	Skin	0	0	0	1
met	Skin	2	4	8	2
met	Skin	2	3	6	2
met	Skin	2	4	8	2
met	Skin	1	3	3	1
met	Skin	2	3	6	2
met	Skin	2	4	8	2
met	Skin	2	4	8	2
met	Skin	0	0	0	1
met	Skin	0	0	0	1
met	Skin	0	0	0	1
met	Skin	2	3	6	2
met	Skin	0	0	0	1
met	Skin	2	2	4	2
met	Small intestine	2	3	6	2
met	Small intestine	2	3	6	2
met	Small intestine	2	2	4	2
met	Small intestine	2	4	8	2
met	Soft tissue	2	2	4	2

met	Lymph node	0	0	0	1	met	Soft tissue	2	1	2	1
met	Lymph node	2	3	6	2	met	Soft tissue	0	0	0	1
met	Lymph node	2	3	6	2	met	Soft tissue	2	4	8	2
met	Lymph node	2	1	2	1	met	Soft tissue	2	4	8	2
met	Lymph node	2	4	8	2	met	Stomach	2	1	2	1

Melanoma TMAs

Human melanoma specimens on tissue microarrays analysed in Fig. 7e and 7f. Expression frequencies of MCAM were scored following the German Immunohistochemical scoring (GIS) system as described in Materials and Methods. The final immuno-reactive score equaled the product of the percentage of positive cells times the average staining intensity. Percentage of MCAM-positive cells was graded as follows: 0 = negative, 1 = up to 10% positive cells, 2 = 11 to 50%, 3 = 51 to 90%, 4 = >90%. Staining intensity of 0 = negative, 1 = weakly positive, 2 = moderately or strongly positive. GIS score ≥ 4 was defined as high and < 4 as low. Prim: primary skin melanoma, met: metastases.

Supplementary Table 4

antibody	manufacturer	catalogue number	dilution	method
ezrin	Cell Signaling Technology	3145	1:50 - 1:100	Immunofluorescence
P-ERM	Cell Signaling Technology	3141	1:50	Immunofluorescence
P-MLC	Cell Signaling Technology	3674	1:25	Immunofluorescence
GFP	Fitzgerald Industries International	20R-GR011	1:500	Immunofluorescence Immunehistochemistry
CD31	Dianova	DIA310	1:40	Immunofluorescence
vimentin	Abcam	ab92547	1:250	Immunofluorescence
MCAM	Abcam	ab75769	1:400	Immunofluorescence
melan A	dako	M7196	1:15	Immunofluorescence
podocalyxin	R&D Systems	MAB1658	10µg/ml	Immunofluorescence
ki67	Thermo Scientific	RM-9106-S0	1:200	Immunofluorescence
NF2	Santa Cruz Biotechnology	sc-55575	1:50	Immunofluorescence
CD49d	BD	340976	1:20	Immunofluorescence
CD44-PE-Cy7	abcam	ab46793	1:20	Immunofluorescence
Integrin-ß1-FITC	Santa Cruz Biotechnology	sc13590	1:50	Immunofluorescence
CD54-FITC	life technologies	MHCD5401	1:10	Immunofluorescence
EpCAM-FITC	dako	F086001F	1: 100	Immunofluorescence
EpCAM-FITC	Miltenyi Biotech	130-080-301	1:10	Immunofluorescence
CD45-APC	Miltenyi Biotech	130-091-230	1:10	Immunofluorescence
gp100-DyLight488	Novus Biologicals	NBP2-34638G	1:50	Immunofluorescence
pericentrin	abcam	ab4448	1:500	Immunofluorescence
cleaved Caspase 3	Cell Signaling Technology	9661	1:300	Immunofluorescence
cl. Casp. 3-Alexa 647	Cell Signaling Technology	9602	1:50	Immunofluorescence
Pan-Keratin-Alexa555	Cell Signaling Technology	3478	1:50	Immunofluorescence
moesin	Cell Signaling Technology	3150	1:300	Immunofluorescence
ki67	NeoMarkers/Lab Vision Corporation	RM-9106-S0	1:200	Immunofluorescence
melanoma gp100	abcam	ab137078	1:500	Immunehistochemistry
CD11a-FITC	life technologies	MHCD11a01	1:50	FACS
CD31-FITC	life technologies	MHCD3101	1:10	FACS
CD44-PE-Cy7	abcam	ab46793	1:20	FACS
CD49d	BD	340976	1:20	FACS
CD54-FITC	life technologies	MHCD5401	1:10	FACS
CD56-PE-Cy7	Beckman Coulter	A21692	1:20	FACS
CD146-FITC	BioLegend	361012	1:20	FACS
Integrin-ß1-FITC	Santa Cruz Biotechnology	sc13590	1:10	FACS
MCAM	Abcam	ab75769	1:20.000	Western Blotting
ezrin	Cell Signaling Technology	3145	1:1.000	Western Blotting
P-MLC	Cell Signaling Technology	3674	1:1.000	Western Blotting
NF2	Abcam	ab88957	1:2.000	Western Blotting
GAPDH	Cell Signaling Technology	2118	1:5.000	Western Blotting

Antibodies

Primary antibodies used for immunohistochemical and immunefluorescence staining and Western Blotting.

Supplementary Table 5

Model	GaussMod	
Equation	double z = (x-xc)/w - w/t0;<br><br>y = y0 + A/t0 * exp(0.5*(w/t0)^2-(x-xc)/t0)*(erf(z/sqrt(2))+1)/2;	
Plot	Peak1(Count)	Peak2(Count)
y0	0.27167 ± 0.25401	0.27167 ± 0.25401
A	48.67995 ± 3.852146	11.29276 ± 2.70959
xc	0.23758 ± 0.08362376803	3.52299 ± 0.28191
w	0.01494 ± 0.0102494	0.58253 ± 0.29832
t0	1.0387 ± 0.08556	1.22951 ± 0.69966
Reduced Chi-Sqr	1.08048601	
R-Square(COD)	0.987403661	
Adj. R-Square	0.983671412	

Fitting parameters
Fitting parameters for the histogram of the Aggregation score shown in Supplementary Figure 1c.
