# Supplementary material for: Single cell polarity in liquid phase facilitates tumour metastasis
Source: Nat Commun. 2018 Feb 28;9:887. doi: 10.1038/s41467-018-03139-6 (PMC5830403; doi:10.1038/s41467-018-03139-6)
Supplement: Supplementary file 3 — Description of Additional Supplementary Files [file 41467_2018_3139_MOESM3_ESM.pdf]

Description of Additional Supplementary Files

File Name: Supplementary Movie 1
Description: Reconstruction from tomograms from 9 consecutive sections through the pole of an SkMel2 cell, generated as described in Methods.

File Name: Supplementary Movie 2
Description: 3D visualisation of a confocal stack of one SkMel cell expressing ezrin-GFP, displaying a cap-like pole.

File Name: Supplementary Movie 3
Description: 3D visualisation of a confocal stack of one SkMel cell expressing ezrin-GFP, displaying a spot-like pole.

File Name: Supplementary Movie 4
Description: Longterm (5h) confocal time-lapse imaging of SkMel2 cells expressing ezrin-GFP on non-adhesive, poly-HEMA-coated substrate. 2D projections of stacks are displayed. The first frame starts 3 hours after detachment of cells. Time format hh:mm. Scale bar: 100µm.

File Name: Supplementary Movie 5
Description: Widefield imaging of one SkMel2 cell expressing ezrin-GFP (shown in Fig. 2f) during detachment. Images were acquired every 30 seconds. Time format mm:ss Scale bar: 10µm.

File Name: Supplementary Movie 6
Description: Widefield timelapse imaging of A375 melanoma cells expressing ezrin-mCherry embedded in a collagen I matrix switching between mesenchymal and amoeboid morphologies, shown as brightfield (left), mCherry (middle) and mCherry in blue/white/red lookup table.

File Name: Supplementary Movie 7
Description: Widefield imaging of one SkMel2 cell expressing ezrin-GFP during detachment. Images were acquired every 30 seconds. Time format mm:ss Scale bar: 10µm.

File Name: Supplementary Movie 8
Description: Widefield imaging of one SkMel2 cell expressing ezrin-GFP during detachment. Images were acquired every 30 seconds. Time format mm:ss Scale bar: 10µm.

File Name: Supplementary Movie 9
Description: Confocal timelapse imaging of a dividing SkMel2 cell expressing ezrin-GFP on non-adhesive, poly-HEMAcoated substrate. Stacks were acquired every 5 minutes. 2D projections of stacks are displayed. The first frame starts 1 hour after release from nocodazole block. Time format hh:mm. Scale bar: 10µm.

File Name: Supplementary Movie 10

Description: 3D visualisation of a confocal stack of one AE cell (from patient sample AE1) stained for ezrin and DAPI.

File Name: Supplementary Movie 11
Description: 3D visualisation of a confocal stack of one CTC (from patient sample CTC(MC)1) stained for ezrin and DAPI.

File Name: Supplementary Movie 12
Description: Confocal imaging of one SkMel2 cell expressing ezrin-GFP, settling onto a plastic surface showing attachment with the pole. Reconstructions of 25 sections are shown in side view. Stacks were acquired every 10 seconds.

File Name: Supplementary Movie 13
Description: Confocal imaging of one SkMel2 cell expressing ezrin-GFP, settling onto a plastic surface showing reorientation of the pole. Reconstructions of 50 sections are shown in top view (left) and side view (right). Stacks were acquired every minute.

File Name: Supplementary Movie 14
Description: Confocal imaging of one SkMel2 cell expressing ezrin-GFP, settling onto a layer of HUVECs, showing reorientation of the pole showing reorientation of the pole. Reconstructions of 50 sections are shown in top view (left) and side view (right). Stacks were acquired every minute.

File Name: Supplementary Movie 15
Description: Confocal imaging of aone unpolarised SkMel2 cell expressing ezrin-GFP, settling onto a plastic surface, showing generation of the pole. Reconstructions of 20 sections are shown in top view (left) and side view (right). Stacks were acquired every minute.

File Name: Supplementary Movie 16
Description: Confocal timelapse imaging of one polarised and one unpolarised SkMel2 cell expressing ezrin-GFP settling onto a plastic surface. Stacks were acquired every minute. 2D projections of stacks are displayed. Time in minutes. Scale bar: 10µm.

File Name: Supplementary Movie 17
Description: Rendering process of the 3D reconstruction shown in Figure 9j ("Polarised", left) of mouse lungs 30 minutes after injection with polarised (30min) SkMel2 cells expressing ezrin-GFP. The first part of the movie shows the original 30 serial 2µm sections stained for GFP (brown), showing seeding of individual cells, the second part of the movie shows the 3D rendering and focal accumulation of GFP+ cells (green). Gridlength: 100µm.

File Name: Supplementary Movie 18
Description: Rendering process of the 3D reconstruction shown in Figure 9j ("Depolarised", right) mouse lungs 30 minutes after injection with depolarised (3h) SkMel2 cells expressing ezrin-GFP. The first part of the movie shows the original 30 serial 2µm sections stained for GFP (brown), showing seeding of individual cells, the second part of the movie shows the 3D rendering and focal accumulation of GFP+ cells (green). Gridlength: 100µm.

File Name: Supplementary Data 1
Description: AttachmenttoAdherenceInVitro.csv is a dataset used for in silico modelling.

File Name: Supplementary Data 2
Description: CellPolarityTissueExtravasation.sbproj is the in silico modelling data provided as one single SBPROJ file that is readable in MATLAB with Simbiology toolbox.

File Name: Supplementary Data 3
Description: CellPolarityTissueExtravasation.xml is the in silico model description in standard SBML format.

File Name: Supplementary Data 4
Description: CirculationToInvitroAdherenceToTissueInVivo.csv is a dataset used for in silico modelling.

File Name: Supplementary Data 5
Description: polarityextravasation_final.opj contains the results of the in silico simulations.

File Name: Supplementary Software 1
Description: EzrinClassifer.py is a single automated Python script for analysis of pole morphology (Supplementary Fig. 1c and Supplementary Table 5, referred to in Methods / Analysis of pole morphology).
