# Supplementary material for: Single cell polarity in liquid phase facilitates tumour metastasis
Source: Nat Commun. 2018 Feb 28;9:887. doi: 10.1038/s41467-018-03139-6 (PMC5830403; doi:10.1038/s41467-018-03139-6)
Supplement: Supplementary file 2 — Peer Review File [file 41467_2018_3139_MOESM2_ESM.pdf]

Editorial Note: this manuscript has been previously reviewed at another journal that is not operating a transparent peer review scheme. This document only contains reviewer comments and rebuttal letters for versions considered at Nature Communications.
